# The Barley (*Hordeum vulgare* ssp. *vulgare*) Respiratory Burst Oxidase Homolog (HvRBOH) Gene Family and Their Plausible Role on Malting Quality

**DOI:** 10.3389/fpls.2021.608541

**Published:** 2021-02-19

**Authors:** Ramamurthy Mahalingam, Danielle Graham, Jason G. Walling

**Affiliations:** United States Department of Agriculture-Agricultural Research Service (USDA-ARS), Cereal Crops Research Unit, Madison, WI, United States

**Keywords:** barley, carbonylation, germination, malting, RBOH, reactive oxygen species, seeds, stress

## Abstract

Controlled generation of reactive oxygen species (ROS) is pivotal for normal plant development and adaptation to changes in the external milieu. One of the major enzymatic sources of ROS in plants are the plasma-membrane localized NADPH oxidases, also called as Respiratory Burst Oxidase Homologs (RBOH). In addition to the six previously reported, seven new members of RBOH gene family were identified in barley using *in silico* analysis. Conservation of genomic structure and key residues important for catalytic activity and co-factor binding was observed in barley RBOH genes. Phylogenetic analysis of plant RBOHs revealed distinct clades for monocot and dicot RBOH proteins. Hence, we propose to use the rice nomenclature for naming barley RBOH genes. Temporal changes in ROS profiles were observed during barley malting and was accompanied by changes in protein carbonylation, lipid peroxidation, and antioxidant capacity. Among the nine differentially expressed HvRBOHs during various malting stages, HvRBOHA and HvRBOHC showed most significant sustained changes in expression. RNAi knockdown lines with reduced expression of HvRBOHA/C gene exhibited genetic compensation via inducible expression of other gene family members during malting. However, the physiological consequence of reduced expression of HvRBOHA/C manifested as a poor malting quality profile attributable to low alpha-amylase activity and high levels of beta-glucan. We propose that the HvRBOHs play a critical role in modulating the redox milieu during the early stages of malting, which in turn can significantly impact carbohydrate metabolism.

## Introduction

Being sessile by nature, plants have evolved mechanisms that usurp their primary metabolic processes to sense and respond to their environment. Reactive Oxygen Species (ROS) are one such metabolic product that has been exploited by plants to regulate their growth and development (Mittler, 2017; Mhamdi and Van Breusegem, 2018). Maintaining a basal concentration of ROS in plants enables proper redox biology reactions and the organization of numerous processes essential for life such as metabolic regulation, cell differentiation and growth, defense against biotic and abiotic stresses, stress acclimation, and signal transduction (Mittler, 2017). However, at higher concentrations, ROS are cytotoxic and can damage cellular macromolecules, such as lipids, proteins and DNA.

One of the sources of ROS in plants is the NADPH oxidase catalyzed conversion of dioxygen (O_2_) to the superoxide radical (O^2–^), which is spontaneously disproportionated to hydrogen peroxide (H_2_O_2_) or by the action of superoxide dismutase enzyme. The plant plasma membrane-localized NADPH oxidases (NOXs) are homologous to the catalytic subunit (gp91phox) of mammalian phagocyte NOXs (Sagi and Fluhr, 2001). Plant NADPH oxidases are known as Respiratory Burst Oxidase Homologs (RBOHs). RBOHs are transmembrane proteins composed of six transmembrane domains supporting two heme groups, FAD and NADPH hydrophilic domains in the C-terminal region and two calcium-binding domains (EF-hand) in the N terminal region. NADPH acts as a cytosolic electron donor to the extracellular O_2_ electron acceptor, which is reduced to O^2–^ via FAD and two independent hemes (Sagi and Fluhr, 2006).

Plant RBOHs constitute a multigene family (Suzuki et al., 2011). One of the earliest characterizations of the RBOH gene family comprising six members was in the model plant Arabidopsis (Torres et al., 1998). With the subsequent completion of the genome sequence, a few more were identified bringing the total to 10 RBOH homologs (Torres and Dangl, 2005). Similarly, in the rice genome, nine RBOH members were reported (Wong et al., 2007) and later two more were identified bringing the total to 11 (Wang et al., 2013). In soybeans, 17 RBOH genes were identified (Liu et al., 2019) while seven were reported in Medicago (Marino et al., 2011) and grapes (Cheng et al., 2013), and four in maize (Lin et al., 2009a). Identification and preliminary characterization of six barley RBOH gene family members indicated conservation with Arabidopsis and rice gene family members (Lightfoot et al., 2008). In this study we took an *in silico* approach to identify seven other members of the HvRBOH gene family.

RBOH-dependent ROS production has been linked to diffuse growth, such as the modulation of cell wall loosening during seed germination and hypocotyl elongation (Muller et al., 2009b). Mutations in the Arabidopsis AtRBOHB gene expressed principally in the seeds has been shown to alter seed germination (Muller et al., 2009a). It has been suggested that the *atrbohB* mutant phenotype reflects the role of ROS in protein oxidation and in the mechanical rupturing of the testa and endosperm through cell wall loosening. One of the pleiotropic phenotypes in the RNAi lines of the barley HvRBOHF was no seed set or reduced number of spikelets per spike (Proels et al., 2010). ROS produced by the NADPH oxidases have been shown to regulate barley seed germination and seedling growth (Ishibashi et al., 2010). The regulation of seed germination by ROS generated via NADPH oxidase was later shown to be mediated through GA/ABA metabolism and signaling in embryo and aleurone cells (Ishibashi et al., 2015). Based on the above-mentioned studies we hypothesized that the RBOH gene family and ROS may play a role during the barley malting process. One of the major industrial uses of barley in the US and Europe is in the production of malt for brewing beer (Xie et al., 2018). Malting is seed germination under controlled conditions and the malting process involves three stages—steeping, germination, and kilning (Mahalingam, 2018). In this study we examined the temporal changes in the ROS accumulation and gene expression changes in the HvRBOH gene family during various stages of malting. RNAi transgenic line with reduced expression of HvRBOH genes exhibited poor malt characteristics suggesting a key biochemical role for members of this gene family in modulating malting quality.

## Materials and Methods

### Sequence Analysis

A search for NADPH oxidase in the PFAM database was used to retrieve all the protein sequences containing this domain. From this set of sequences, proteins that were present in barley were identified for further analysis. The barley sequences were carefully analyzed to identify other domains associated with the well characterized Arabidopsis RBOH gene family.

The 14 sequences from barley along with rice OsRBOHA and Arabidopsis AtRBOHF sequence were used for multiple sequence alignments using the T-Coffee program (Notredame et al., 2000).

### Phylogenetic Analysis

Plant RBOH sequences were retrieved from Phytozome and Ensembl databases. This analysis involved 55 protein sequences: 17 from soybeans, 13 from barley, 10 from Arabidopsis, 9 from rice, 4 from maize, 1 from the moss Psycomitrella patens, and 1 from lycophyte Selaginella. The full-length coding sequences of these RBOH genes were aligned by the Muscle program with default parameters (Edgar, 2004). Phylogenetic trees were constructed using the neighbor-joining (NJ) method with a bootstrap analysis of 1,000 replicates (Stecher et al., 2020). The evolutionary distances were computed using the p-distance method (Nei and Kumar, 2000) and are in the units of the number of amino acid differences per site. All ambiguous positions were removed for each sequence pair (pairwise deletion option). There were a total of 1,417 positions in the final dataset. For the 13 HvRBOH protein sequences, initial tree(s) for the heuristic search were obtained automatically by applying Neighbor-Join and BioNJ algorithms to a matrix of pairwise distances estimated using the Jones-Taylor-Thornton model, and then selecting the topology with superior log likelihood value. Evolutionary analyses were conducted in MEGA X (Kumar et al., 2018; Stecher et al., 2020).

### Plant Growing Conditions

Barley varieties Conrad, Golden Promise and the transgenic RNAi line with reduced expression of HvRBOHA/C in the Golden Promise background (Proels et al., 2010) were grown in greenhouse with 16 h light (400 μmol m^–2^ s^–1^) at 22°C and 8 h of darkness at 18°C. The dry mature spikes or heads from each plant was collected and were later threshed using the bench top thresher (Model LT15; Haldrup, Poneto, IN).

### Micromalting

One hundred grams of seeds of variety Conrad, Golden Promise, and RNAi line for HvRBOHA/C were subjected to the standard micromalting procedure established in the Malt Quality Lab at the USDA Cereal Crops Research Unit, as described earlier (Mahalingam, 2018). Briefly this includes the following steps

#### Steeping

Seeds were placed in cuboidal stainless−steel steeping cans with screen mesh bottom. The regimen included cycles of 4 h immersion (16°C) in water and 4 h air rest (18°C) during a 28 h steep period. The targeted steep-out moisture content was 45%.

#### Germination

Steeped samples were placed in stainless steel cylindrical cans and samples were germinated for 120 h at 17°C and >98% humidity, with turning every 0.5 h.

#### Kilning

Germinated seeds were placed in kilning cylindrical steel cans with mesh bottoms. During kilning, hot air was blown through the samples in a slow, controlled manner. The program lowers finished malt moisture to approximately 4.0%, over 24 h, and consists of the following stages: 49°C for 10 h, 54°C for 4 h, 60°C for 3 h, 68°C for 2 h, and 85°C for 3 h.

Seed samples were collected at end of steep, 1, 2, 3, 4, 5 days after germination, and end of kilning stage, immediately frozen in liquid nitrogen and stored in –80 C until further processing. Two independent malting runs were conducted for each of the three barley lines used in this study.

### qPCR Analysis of HvRBOH Gene Family

Total RNA was isolated from the following tissues of Conrad: root, stem, and leaf tissue from 3 week old seedlings and flowers from older plants. RNA of barley variety Golden Promise, RNAi line with reduced expression of HvRBOHA/C, along with standard malting check variety Conrad were isolated from seeds and different stages of malting (end of steeping, 1, 2, 3, 4, and 5 days after germination). Two independent RNA isolations were done for each tissue and malting time point using the RNeasy plant mini kit (Qiagen). One microgram of RNA was used for cDNA synthesis. Two independent cDNA synthesis reactions were set up from each RNA sample. Freshly synthesized cDNA was used for qPCR analysis as described earlier (Walling et al., 2018). The primers for the newly identified HvRBOH genes are given in Supplementary Table 1. The primer sequences for the six earlier identified HvRBOHs were from Lightfoot et al. (2008). Two endogenous genes—HSP70, and GAPDH stably expressed during various malting stages (Walling et al., 2018) were used for normalization in this study. For determining the differences in the expression of HvRBOHs in different tissues the median cT value for each tissue was subtracted from the gene-specific cT values. Heat map showing the relative differential expression of the HvRBOHs in different tissues was generated using the Heatmapper online tool^1^. Relative expression ratio of HvRBOHs during the various malting stages were calculated based on 2^–δ*Ct*^, where δCt = Ct_*HvRBOH*_ – Ct_control gene_ and using the expression in the dry seeds as the calibrator.

### Measuring ROS Levels During Malting

Four seeds of Conrad from each of the different stages of malting were ground to a fine powder in liquid nitrogen and 100 mg was resuspended in 500 μl of 50 mM sodium phosphate buffer (pH 7.4) and their weights were recorded. The samples were centrifuged at 10,000*g* for 5 min and the supernatant transferred to a new tube. Samples were aliquoted in triplicate (50 μl each) in a 96-well plate. A master mix containing 50 μl of the Amplexred reagent in DMSO, 100 μl of horseradish peroxidase and 4.85 mL of the phosphate buffer was prepared and 50 μl of this mix was added to each well in the 96-well plate. Hydrogen peroxide dilution series ranging between 0.1 and 10 μM was prepared simultaneously. The 96-well plate was placed on a plate mixer (Eppendorf) to mix for 30 s at 300 rpm. Plate was placed in the plate reader (Synergy H1, Biotek, Winooski, VT, United States) and the absorbance at 570 nm was measured immediately and at 10 min intervals for 30 min. The experiments were repeated five times using seeds from two different malting runs. In one of these replications, catalase (300 Units/mL) was added to all the sample wells.

### Histochemical Staining of ROS During Malting

Barley seeds of Conrad, Golden Promise and the HvRBOHA/C knock-down were collected from different stages of malting and were incubated for 30 min in 0.1% (w/v) nitroblue tetrazolium (NBT) in 10 mM sodium azide and 50 mM potassium phosphate buffer (pH 6.4) to identify superoxide anion. To detect hydrogen peroxide, seeds were incubated in 4.7 mM 3,3′ diaminobenzidine (DAB) (prepared by dissolving DAB in water and pH lowered to 3.8 using 10N HCl) under a vacuum for 30 min. Seeds were hand-cut longitudinally using a razor blade. A few seeds from each variety were soaked for a period of time in MilliQ water and were cut longitudinally and imaged as a negative control. Imaging was done using a Zeiss microscope and photographed using a Nikon camera attached to the scope.

### Analyzing Protein Oxidation During Malting

Protein extractions from the various stages of malting barley were conducted using the procedures described previously (Job et al., 2005; Zhang et al., 2016). Approximately 150 mg of seed was ground with a mortar and pestle and liquid nitrogen and the resulting powder was homogenized in 1 ml buffer containing 250 mM sucrose, 10 mM EGTA, 1 mM Protease inhibitor cocktail, 10 mM Tris-HCl, pH 7.5, 1% (v/v) Triton X-100, 1 mM PMSF and 1 mM DTT and then centrifuged at 15,000*g* for 20 min at 4°C. The supernatant was submitted to a second clarifying centrifugation as above. The final supernatant corresponded to the total protein extract. Supernatant thus obtained was mixed with three volumes of acetone and left overnight at −20°C. Following centrifugation, the protein pellets were washed with acetone three times and allowed to air dry. The pellet was dissolved by vortexing in lysis buffer containing 0.1%SDS, 1%CHAPS, 0.1M NaCl, 0.1M sodium phosphate, 50 mM DTT and 1 mM EDTA (pH 7.5). Protein concentrations in the various extracts were measured according to Bradford (1976) using bovine serum albumin as a standard and protein quality was assessed by 1D-SDS PAGE (Supplementary Figure 1).

About 50 μg of the proteins were used for derivatization with 2,4-Dinitrophenylhydrazine (DNPH) using the procedure outlined in the Oxyblot Kit manual (Millipore). Equal amounts of protein without the DNPH were subjected to the same procedure and was used as non-derivatized control. Proteins were separated on a 12% protean gel and transferred to PVDF membrane (Bio-Rad, Marnes La Coquette, France) using turbo-mini TGX gel procedures. Blots were rinsed twice for 5 min in 50 mM Tris-HCl, 150 mM NaCl, pH 7.5 (TBS), and then incubated overnight in blocking solution containing 1X TBS, 0.05% tween and 1% BSA. Blots were incubated at room temperature for 2 h in 15 ml fresh blocking solution containing 100 μl of primary DNPH antibodies. Membrane was washed twice in PBST for 5 min and then incubated for 1 h at room temperature with 15 ml blocking solution containing secondary antibody (1:300). After rinsing the membrane twice for 5 min in PBST, 5 ml of luminol was added and incubated for 2–3 min and the chemiluminescent signal were captured using an UVP Autochemi system (Upland, CA). Relative protein carbonyl levels were quantitated by densitometric analyses of the western blots.

### Measuring Lipid Peroxidation During Malting

The lipid peroxidation protocol previously described was modified for this study (Jambunathan, 2010). Barley seeds (approx. 300 mg) from each stage of malting was ground to fine powder with pestle and mortar using liquid nitrogen. Finely ground powder was transferred to a tube containing 1.0 mL 0.1% TCA, vortexed, and allowed to sit at room temperature for 10 min. The extracts were centrifuged at 16,000*g* for 10 min. The supernatant was collected, and 1 mL supernatant was mixed with 500 μl of 20% TCA and 500 μl of 0.5% TBA and vortexed. Malondialdehyde tetrabutylammonium salt (MDA) (Sigma) was used for preparing the standards. The 2 mL tubes were sealed with no-pop tops before placing in water bath at 85°C for 1 h. Tubes were then cooled on ice. Following centrifugation at 16,000*g* for 5 min, 200 μl of sample/well were transferred to 96-well plate. The plate was placed on a shaker at 1,000 rpm for 1 min. The absorbance at 532 and 600 nm against a blank solution (1 ml of 0.1%TCA + 500 μl of 20%TCA and 500 μl of 0.5% TBA) was read using a plate reader (Synergy H1, Biotek, Winooski, VT, United States). *A_600_*, the non-specific absorbance was subtracted from the values for *A_532_.* The concentration of MDA in the seed samples were estimated using the standard curve.

### Measuring Antioxidant Capacity and Tocols During Malting

Oxygen radical scavenging capacity (ORAC) was used to measure the antioxidant capacity of the samples during various stages of the malting process as described (Gillespie et al., 2007). Trolox was used as a standard and the assay was set up in a 96 well plate and was placed in a plate reader and was set to run a fluorescence kinetic read with an excitation wavelength of 485 nm and an emission wavelength of 530 nm at 37°C for 1 h. Fluorescence activity of a sample extract and a blank was recorded. The oxidation of the fluorescent compound was determined by the antioxidants present in the sample. The ORAC activity of a sample is calculated by subtracting the area under the blank curve from the area under the sample curve to obtain the net area under the curve. The samples were analyzed in triplicates and the whole assay was repeated three times.

All the eight different isoforms of tocochromanols were analyzed using HPLC at the various stages of malting using a procedure described earlier (Mahalingam et al., 2020). This analysis was repeated twice and the average of the two runs are presented.

### Malt Quality Analysis

The key malting quality parameters—total protein content, soluble/total protein, malt extractability, free amino nitrogen (FAN), beta-glucan content, alpha amylase activity, and diastatic power in these malted samples were analyzed as described earlier (Mahalingam, 2017). From each replication of the micromalted samples, the various parameters listed above were measured twice (*n* = 4).

### Statistical Analysis

Student’s *t*-tests were used to determine the statistically different differences in the various parameters/traits among the different stages of malting barley. All the comparisons were done with reference to the values in the dry seeds. For the malt quality data, statistically different traits were determined for the HvROHA/C knock-down line by comparisons with values observed in the non-transformed varietal version of Golden Promise.

## Results

### Thirteen HvRBOH Genes Are in the Barley Genome

A search for the NADPH oxidase domain in the PFAM database retrieved 1,032 sequences represented in 80 different species. Among these, 98 sequences were identified in the barley genome (Supplementary Figure 2). A careful examination of these 98 barley protein sequences indicated many of them were alternative splice variants of a few genes and the number of unique gene identifiers associated with these sequences was only 14 (Supplementary Text 1). Among these, 13 protein sequences were found to have the characteristic calcium binding EF hand motif in their N-terminus and hence were considered as putative members of the barley HvRBOH gene family (Table 1). This set included the HvRBOHA, first identified in response to powdery mildew infection in barley (Trujillo et al., 2006) and six proteins later identified based on genome walking and BAC library screening (Lightfoot et al., 2008). It has been reported that the RBOH originally identified as HvRBOHA and the later identified HvRBOHF2 represented the same protein (Proels et al., 2010). Thus, based on this *in silico* analysis, seven new putative RBOH members were identified in the barley genome.

**TABLE 1:** Details of the barley HvRBOH gene family members.

	Ensembl gene ID	Proposed gene symbol	GenBank accession	ORF (bp)	Protein (amino acids)	Splice variants	No. of exons	Other identifiers
1	HORVU1Hr1G071340	HvRBOHD	MW520099	3,036	1,011	40	14	MLOC_11469
2	HORVU1Hr1G072140	HvRBOHI1	MW520100	2,790	929	3	10	MLOC_69263
3	HORVU1Hr1G072160	HvRBOHI2	MW520101	2,808	935	12	10	MLOC_53927 RBOHB
4	HORVU1Hr1G081950	HvRBOHC	MW520102	2,907	968	13	14	HvRBOHA/F2
5	HORVU3Hr1G037600	HvRBOHB2	MW520103	2,658	885/459	21	12/6	HvRBOHB2
6	HORVU3Hr1G069780	HvRBOHA	MW520104	2,814	937	22	14	HvRBOHF1
7	HORVU3Hr1G087210	HvRBOHE	MW520105	2,601	866	27	13	HvRBOHJ MLOC_63739
8	HORVU4Hr1G081670	HvRBOHI3	MW520106	2,934	977	6	9	MLOC_55429
9	HORVU4Hr1G086500	HvRBOHB1	MW520107	2,538	845	22	13/12	HvRBOHB1
10	HORVU5Hr1G062490	HvRBOHG	MW520108	2,964	987	17	13	HvRBOHE; MLOC_17615
11	HORVU5Hr1G024550	HvRBOHH	MW520109	2,322	773	13	10	MLOC_5633
12	HORVU5Hr1G078630	HvRBOHI4	MW520110	2,889	962	3	1	MLOC_73746
13	HORVU6Hr1G035970	HvRBOHF	MW520111	2,880	959	28	12	MLOC_77866

*GenBank accession numbers are for the longest open reading frame of HvRBOH genes that was used in the phylogenetic analysis.*

### Monocot and Dicot RBOHs Group Into Distinct Phylogenetic Clades

**FIGURE 1:**
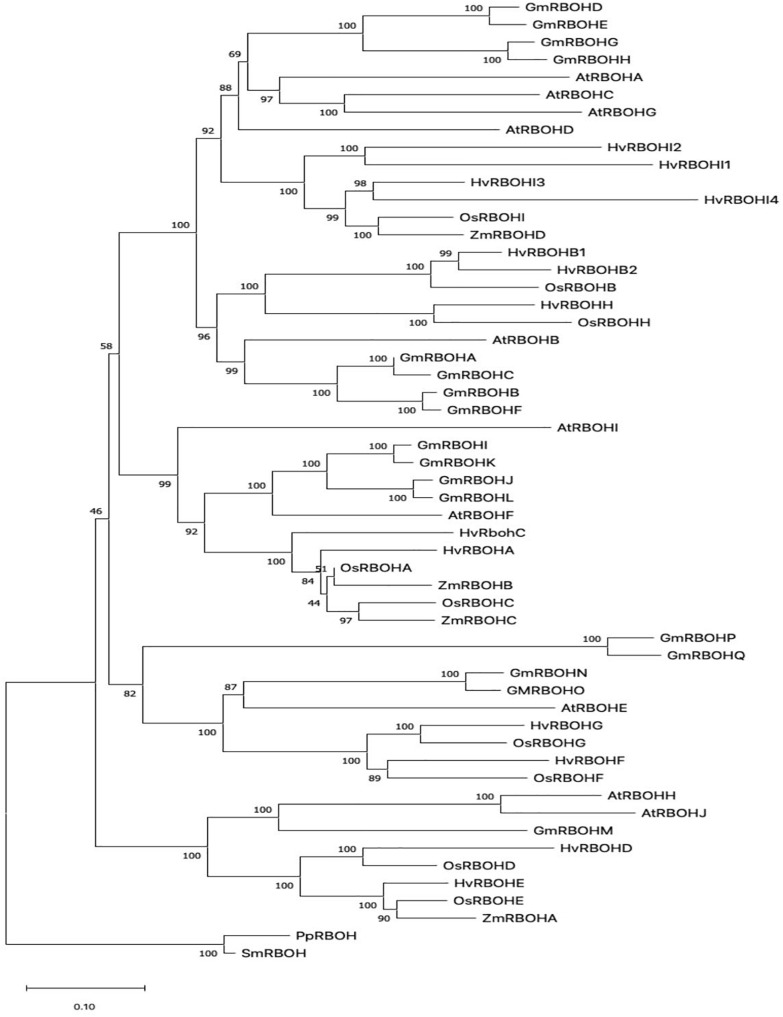
The evolutionary history of plant RBOH protein sequences inferred using the Neighbor-Joining method. The percentage of replicate trees in which the associated taxa clustered together in the bootstrap test (1,000 replicates) are shown next to the branches (Felsenstein, 1985). The tree is drawn to scale, with branch lengths in the same units as those of the evolutionary distances used to infer the phylogenetic tree.

In order to infer the evolutionary relationships among plant RBOHs, the HvRBOH amino acid sequences were compared with each other and with RBOH protein sequences from two other monocots namely rice, and maize, and two dicots—Arabidopsis and soybeans. The RBOH sequences from moss and lycophyte formed a monophyletic clade. RBOH proteins from monocot and dicot groups formed distinct clades (Figure 1). This suggests specialized roles for the members of this gene family that are unique to monocots or dicots. Based on these results, we name the newly identified HvRBOH gene family according to their orthologous rice genes. We also propose to rename the six previously identified HvRBOHs (named based on Arabidopsis genes) using their phylogenetic affiliations with rice RBOHs. Of the seven newly identified HvRBOH genes, four belong to same clade as the rice OsRBOHI gene while the others were closely related to the rice OsRBOHD, OsRBOHE, and OsRBOHH genes.

### Chromosomal Distribution of RBOHs in Barley Genome

Four HvRBOH genes (HvRBOHD, HvRBOHI1, HvRBOHI2 and HvRBOHC) were identified on the bottom of chromosome 1 and two on the bottom of chromosome 4 (HvRBOHI3 and HvRBOHB1) (Supplementary Figure 3 and Table 1). Chromosome 3 (HvRBOHB2, HvRBOHA and HvRBOHE) and 5 (HvRBOHG, HvRBOHH, HvRBOHI4) each had three HvRBOH genes, while one HvRBOH gene was located on chromosome 6 (HvRBOHF). The gene sizes ranged between 2.9 and 4.2 kb while the protein lengths were between 845 amino acids and 1,011 amino acids. *In silico* prediction of localization indicated that the HvRBOH proteins were in the plasma membrane and is further supported by 1–6 transmembrane domains identified in these proteins. Another interesting feature of the HvRBOH gene family was that all the members have *in silico* predicted alternate splice variants. The least number of predicted splice variants was three and the highest number of variants was 40 (Table 1).

### Domain Organization of HvRBOHs Is Similar to Other Plant RBOH Proteins

**FIGURE 2:**
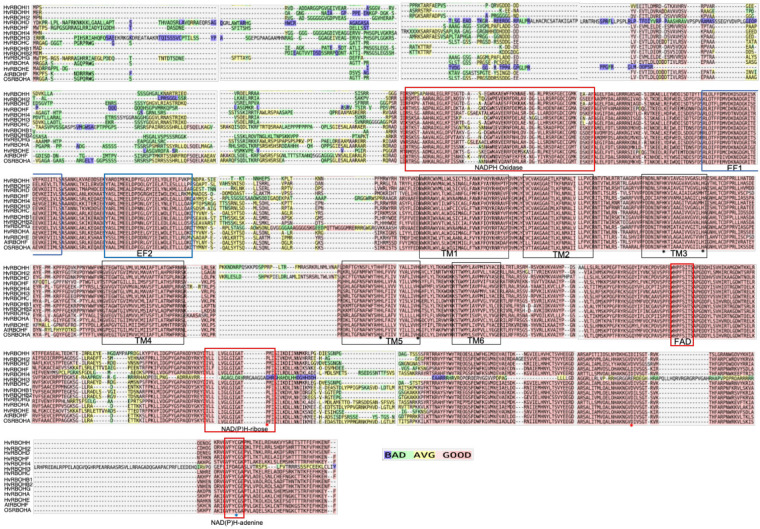
T-Coffee based alignment of HvRBOH protein sequences. The Arabidopsis AtRBOHF and the rice OsRBOHA sequences are also included in this analysis. The color scale represents good, average and poor alignment among these sequences. EF hand domains are indicated by blue boxes. The NADPH oxidase and other co-factor binding domains are marked with red boxes. The five transmembrane domains are enclosed in black boxes. Histidine residues important for heme binding are shown with black asterisk, conserved proline and aspartate residues for enzyme activity are marked by red asterisks and the conserved cysteine residue for nitrosylation is indicated by a blue asterisk.

The amino acid sequences of the barley RBOH homologs were aligned with Arabidopsis AtRBOHF and rice RBOHA proteins to identify conserved domains (Figure 2). Juxtaposed to the conserved NADPH oxidase domain, the N-terminal regions of the 13 HvRBOH proteins contain two putative Ca^2+^-binding EF-hand. In the middle region of the HvRBOH proteins six transmembrane domains (TM1-6) were identified and corresponds to those identified in plant RBOHs from Arabidopsis, rice, maize, potato, tobacco, as well as the mammalian gp91^*phox*^. TM3 and TM5 also bears a pair of conserved histidine residues important for heme binding. In the C-terminus of these proteins there are conserved motifs associated with binding of three metabolites namely, FAD, NADPH-ribose, and NADPH-adenine. The conserved proline and aspartate residues in the C-terminus important for the catalytic activity of the enzyme and the redox sensitive cystine residue (position 890) was conserved among 12 HvRBOH proteins except for HvRBOHI4 (Figure 2).

### Exon-Intron Structure of HvRBOH Genes

**FIGURE 3:**
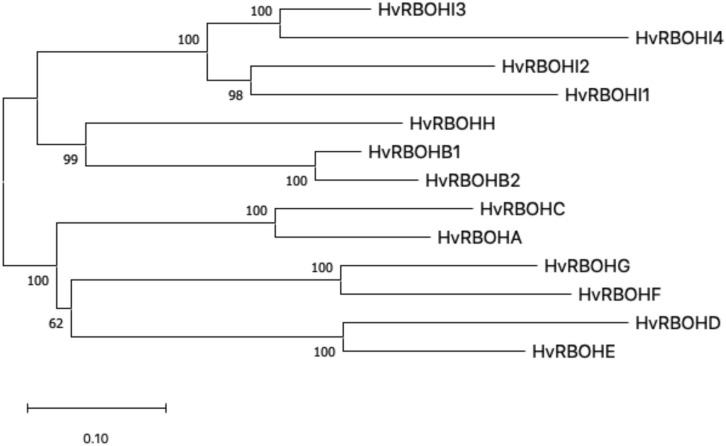
The evolutionary history of HvRBOH protein sequences inferred using the Maximum Likelihood method and JTT matrix-based model. The tree is drawn to scale, with branch lengths measured in the number of substitutions per site.

**FIGURE 4:**
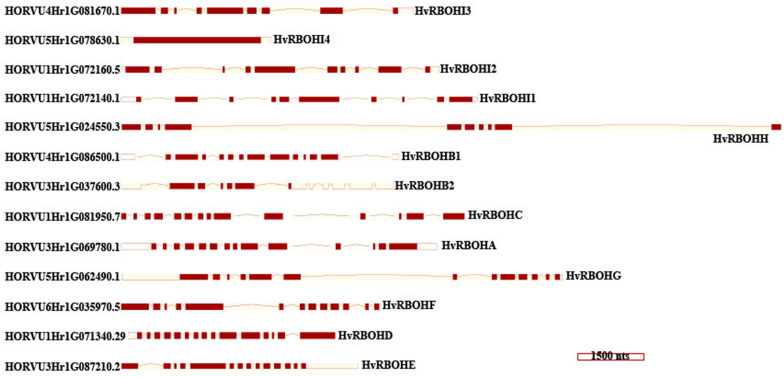
HvRBOH gene models retrieved from the Ensembl database. Longest coding isoforms for each of the barley gene models are shown and their corresponding gene identifiers are on the left. The newly proposed gene names for the HvRBOH genes based on their groupings with the corresponding rice genes are shown on the right. The brown colored boxes represent the exons, the connecting lines represent the introns. The models are drawn to scale shown at the bottom and arranged to show the similarity with the phylogenetic groupings in Figure 3.

An unrooted phylogenetic tree was constructed using the longest coding sequences of each of the 13 HvRBOH proteins (Figure 3). Exon-intron structures were retrieved from the barley Ensembl database for these variants (Figure 4). The number of exons ranged from 9 to14 with three sets of HvRBOH genes each bearing 10 or 13 or 14 exons, two genes with 12 exons, one gene with 9 exons. In general, the C-terminus of the gene had a number of smaller sized exons. HvRBOHB1 and B2 were annotated to have 6 or 12 coding exons while the actual number of identified exons was 12 or 13, respectively. The increase in exon number seen in several of the barley genes appears to be a result of insertions of introns into the exonic regions, rather than from acquisition of additional exons. The order and approximate size of exons among the barley genes was relatively conserved, while intron size was more variable. The phylogenetic tree groupings based on protein sequences was consistent with the intron-exon distribution patterns among the HvRBOH gene sequences (Figures 3, 4). The long branch length of HvRBOHI4, being intronless and lacking several crucial amino acid residues described above for binding and catalytic activity, strongly suggested that this gene may not be a *bonafide* RBOH.

### Different Barley Tissues Exhibit Unique Transcriptional Profile for HvRBOHs

**FIGURE 5:**
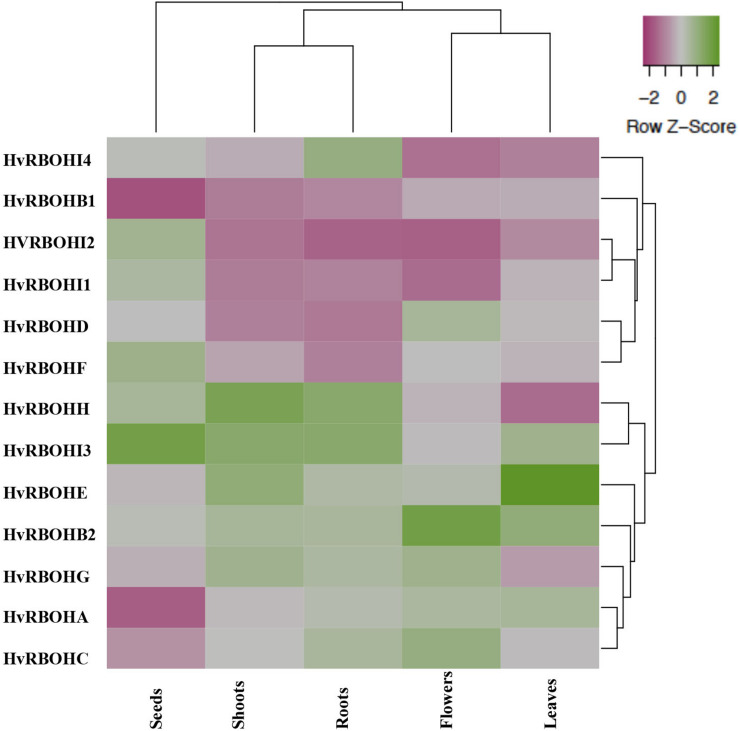
Expression of the HvRBOH genes in different barley tissues. The relative expression of each HvRBOH gene was used for plotting the heat map. Average linkage clustering using Euclidean distances was used for clustering the HvRBOH genes and tissues. Green shade represents relative expression values greater than the median and pink shades represent expression values lower than the median across each tissue.

Expression of the HvRBOH genes were examined in five different barley tissues, namely, seeds, young roots, leaves, shoots, and flowers (Figure 5). The expression of the HvRBOHs were stronger in roots and were weaker in the seeds (Supplementary Table 2). Based on the phylogenetic grouping of the qPCR data, expression patterns of HvRBOH genes in roots and shoots were similar as were the expression in leaves and flowers. In the seeds, expression of six HvRBOHs were lower than the median (HvRBOHA, HvRBOHB1, HvRBOHC, HvRBOHD, HvRBOHE, and HvRBOHG), five were higher than median (HvRBOHI1, HvRBOHH, HvRBOHI2, HvRBOHF, and HvRBOHI3). Closely related HvRBOH pairs (Figure 3) exhibited similar expression patterns in various tissues as observed for HvRBOHA and C, HvRBOHI1 and I2. On the contrary, a few phylogenetically closely related HvRBOHs also showed very different expression patterns as was observed for HvRBOHG and HvRBOHF, and HvRBOHD and HvRBOHE.

### HvRBOH Genes Are Differential Expressed During Barley Malting

**FIGURE 6:**
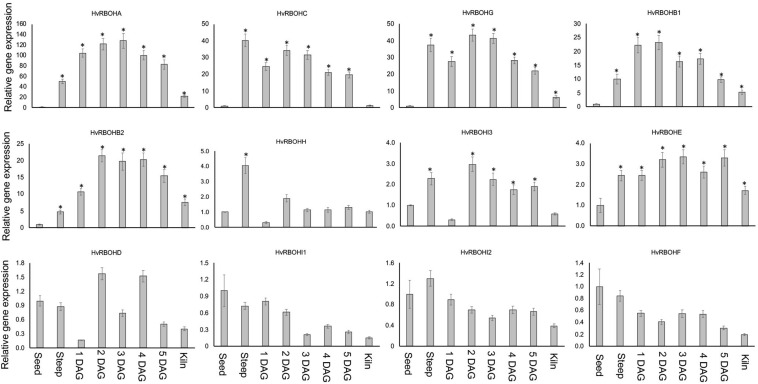
Expression of the HvRBOH genes during different stages of malting in variety Conrad. Relative expression of each gene in various malting stages was with reference to their expression in dry seeds. Error bars represent standard error of the average cT values (*n* = 4). ^∗^Represent significantly different changes (>log_2_-fold) in the HvRBOH expression when compared to their expression levels in the dry seeds (*p* < 0.05). HSP70 was used for normalization.

Expression of the HvRBOH genes were examined at seven different stages of the malting process—steep, 1, 2, 3, 4, 5 days after germination and end of kilning stage. The expression of HvRBOHs in the dry seeds were also analyzed. It is important to point out that at the end of steep regime the physiological *sensu stricto* germination is completed as tiny radicles become visible and in malting jargon is referred to as chitting. In the malting literature, the term “days after germination” refers to a expression of the time period after transferring the steeped seeds into the germination chambers and thus should not be interpreted as the precise time interval after canonical germination, which is defined at the physiological level and would have occurred sometime during the steep stage. The most dynamic changes in expression were observed for HvRBOHA that showed a 50-fold increase at steep, and more than 100-fold increase during most stages of germination (Figure 6). Four genes (HvRBOHB1, HvRBOHB2, HvRBOHC, and HvRBOHG) show very high expression during most of the seven stages tested (Figure 6). Of these, HvRBOHC showed highest expression during steeping, maintained a high level of expression during germination and dropped to lowest level during kilning compared to its expression in seeds. HvRBOHG also showed bi-phasic induction pattern with a 37-fold increase in steep when compared to its expression in dry seeds, followed by a reduction to 27-fold by 1 DAG, and then increased to more than 40-fold during days 2 and 3 of germination. HvRBOHH exhibited a fourfold increase at steep stage while HvRBOHI3 and HvRBOHI4 showed a nearly threefold increase in their expression at 2 DAG stage (Figure 6). HvRBOHE showed a sustained 2 to 3-fold increase in its expression during steeping and all stages of germination. Four other HvRBOH genes (HvRBOHD, HvRBOHI1, HvRBOHI2, and HvRBOHF) did not show significant changes in their expression during the various stages of malting (Figure 6).

### Significant Accumulation of ROS Occurs During Early Stages of Malting

**FIGURE 7:**
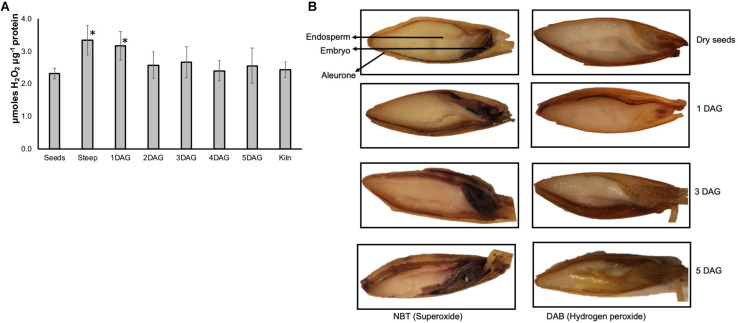
Temporal and spatial variations in ROS during barley malting. **(A)** Changes in the ROS profile determined using Amplexred during various stages of barley malting. Bars represent standard error (*n* = 5). ^∗^Represent significantly different ROS levels in the sample when compared to the ROS levels in the dry seeds (*p* < 0.05). **(B)** Histochemical localization of ROS during barley malting. The blue staining of the embryos and aleurone layer indicates presence of superoxide and the brownish stain in these tissues shows the presence of hydrogen peroxide at different stages of germination during malting of barley variety Conrad.

The amount of hydrogen peroxide increased by more than 60% at the end of steep and 1 day after germination compared to their amounts in the dry seed sample (Figure 7A). The levels of H_2_O_2_ remained slightly higher at other time points during the malting process compared to dry seeds, however this increase was not statistically significant. Addition of catalase to the extracts reduced the detected signal to background levels confirming that the assay was quantifying the H_2_O_2_ present in these samples (data not shown).

### ROS During Malting Is Localized to Embryo and Aleurone Layers

Barley seeds collected from various stages of malting process were histologically assayed for two major types of ROS. To assay for superoxide, seeds were stained with NBT, and for detecting H_2_O_2_ accumulation DAB staining was performed. Accumulation of both superoxide and H_2_O_2_ was observed in the dry seeds (Figure 7B). Conrad seeds, at end of steep, 1, 3, and 5 days after germination showed increasing intensity of staining for both of these ROS in the embryo and aleurone layer but was distinctively absent in the endosperm tissue.

### Oxidation of Proteins and Lipids Occur During Malting

**FIGURE 8:**
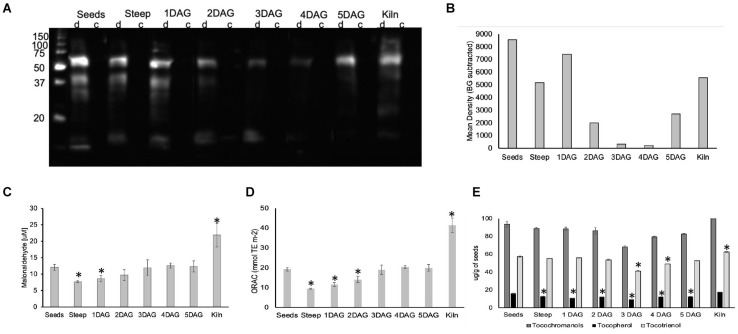
Impact of ROS generated during barley malting on various biomolecules. **(A)**. An image of the western blot of the anti-DNP immunoassay for detecting oxidatively modified proteins of barley seeds during malting. **(B)** Membrane was scanned, and the mean background was calculated from the control (c) lane for each sample. The background subtracted signal intensity for each derivitazed (d) sample was used for plotting the graph. Representative results are shown. **(C)** Changes in lipid peroxidation during barley malting stages. ^∗^Represent significantly different MDA levels in the sample when compared to their levels in the dry seeds (*p* < 0.05). Error bars represent standard error (*n* = 5). **(D)** Changes in the total antioxidant capacity during various stages of barley malting. Error bar represents the standard error (*n* = 5). ^∗^Represents statistically significant changes in the antioxidant capacity when compared to dry seeds (*p* < 0.05). **(E)** Changes in the total tocochromanols, tocopherols, and tocotrienols during various stages of barley malting. Error bar represents the standard error (*n* = 4). ^∗^Represents statistically significant changes in these metabolites when compared to their measured quantities in the dry seed sample (*p* < 0.05).

To determine whether ROS during malting impacted protein oxidation (carbonylation), one-dimensional (1D) PAGE of seed protein extracts were performed, and the presence of carbonyl groups was detected by Western blotting using the 2,4-dinitrophenylhydrazine (DNPH) immunoassay (Job et al., 2005). Soluble proteins from seeds exhibited three carbonylated bands of approximately 60, 40, and 30 kDa and a faint band of about 28 kDa (Figure 8A). In the steep sample, these major bands were visible, however, their intensity was nearly 50% lower compared to the seed sample. In the 1 DAG sample the pattern was similar to the steep however there was a smear in the lower molecular weight range (20–35 kDa). The extent of carbonylation in the other stages of germination (2–5 DAG) was significantly lower compared to the dry seeds (Figure 8B). In the kilned seeds, the carbonylation levels increased to levels comparable to the steep sample for the 60 kDa band, but also exhibited two other fainter bands that was approximately 45 kDa and a smaller band that was slightly more than 20 kDa in size.

The changes in the total lipid oxidation determined based on malonaldehyde concentration was monitored during the malting process (Figure 8C). A significant reduction in the concentration of oxidized lipids were observed at the end of steeping cycle and the earliest stage of germination. However, the levels of the oxidized lipids at later stages of germination were comparable to those in dry barley seeds. The amount of oxidized lipids was nearly twofold higher at the end of kilning stage.

### Antioxidant Capacity Is Lowered During Early Stages of Malting

There was a significant reduction of nearly 50% in the antioxidant capacity at the end of steep and the early stages of germination (Figure 8D). The antioxidant capacity was restored to the levels observed in the dry seeds during the later stages of germination. It was interesting to note that the antioxidant capacity in the kilned seeds almost doubled when compared with the dry seeds.

In cereal seeds, tocotrienols are more abundant compared to tocopherols. In micromalted barley, we observed that the tocopherol levels were slightly reduced throughout the various stages of malting when compared to their amounts in the dry seeds (Figure 8E). The tocotrienols were stable during steep and early stages of germination but showed a significant reduction at later stages of germination. Significant increases in tocotrienols were observed in the seeds after the kilning cycle (Figure 8E). Alpha tocotrienol levels were much higher compared to the other vitamin E isoforms and showed a significant increase in the kilned seeds (Supplementary Figure 4).

### Expression of RBOH Family Members in the HvRBOHA/C Knock-Down Line During Malting

**FIGURE 9:**
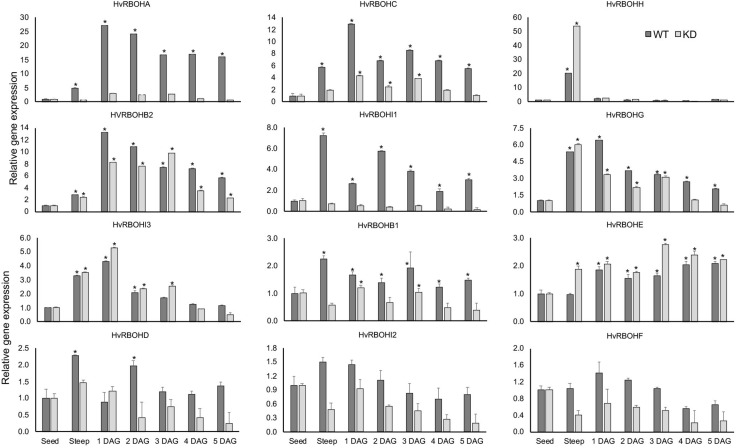
Expression of the HvRBOH genes during different stages of malting in the HvRBOHA/C knockdown line and wildtype Golden Promise variety. Relative expression of each gene in various malting stages was with reference to their expression in dry seeds. Error bars represent standard error of the average cT values (*n* = 4). ^∗^Represent significantly different changes (>log_2_-fold) in the HvRBOH expression when compared to their expression levels in the dry seeds (*p* < 0.05). GAPDH was used for normalization.

As expected, the expression of HvRBOHA and HvRBOHC genes in the knock-down line were greatly reduced when compared to their expression in the wildtype Golden Promise (Figure 9). However, it was observed that the differences in the inducibility of these two genes in Golden Promise were lower compared to Conrad. At the end of steep stage, the expression of HvRBOHH gene was induced 50-fold in the knock-down line compared to the 20-fold increase observed in the wildtype. In the knockdown line HvRBOHI3 showed increased expression at 1 and 3 DAG, and HvRBOHE at steep and 3 DAG when compared to their expression in wildtype. Downregulating HvRBOHA/C also seemed to negatively impact the expression of HvRBOHI1 and HvRBOHI2 whose expression showed a modest increase during various stages of malting in the wildtype. Thus, down regulating HvRBOHA/C overall leads to changes in expression of other gene family members during various stages of the malting process (Figure 9). ROS staining patterns in the HvRBOHA/C knock-down mutant and Golden Promise were similar to that in Conrad (Supplementary Figure 5).

### Malting Quality of the HvRBOHA/C Knockdown Transgenic Line Is Altered

We used eight malting traits to assess the overall malt quality of the lines and found the HvRBOHA/C knockdown lines would not qualify using standard malting industry recommended cutoffs for seven of traits measured (Table 2). Golden Promise also did not fare well when compared to the standard malting variety Conrad. However, for six of the eight traits analyzed, the performance of the HvRBOHA/C knockdown was negatively impacted compared to their values in Golden Promise. Total protein content, percentage of soluble to total protein, diastatic power and beta-glucans increased while malt extractability and alpha-amylase content were lowered in the knock-down line compared to their values in the Golden Promise.

**TABLE 2:** Malting quality of Conrad, *HvRBOHA/C* knock-down line and WT Golden Promise.

	Malt extract	Barley protein	Wort protein	S/T	DP	AA	Beta glucan	FAN
Recommended	>81	<13.0	4.8–5.6	40–47	>140	>50	<100	>210
Conrad	81.2 ± 0.3	12.2 ± 0.1	5.6 ± 0.4	46.5 ± 1.0	154 ± 10	92.1 ± 3.5	172 ± 26	277 ± 12
HvRBOHA/C KD	**71.6 ± 0.2**	**18.7 ± 0.1**	4.7 ± 0.4	**32.9 ± 1.4**	**224 ± 21**	**36.7 ± 3.0**	**818 ± 44**	146 ± 15
Golden promise WT	**72.8 ± 0.1**	**17.6 ± 0.1**	4.5 ± 0.4	**26.1 ± 1.2**	**168 ± 12**	**45.7 ± 2.8**	**716 ± 28**	147 ± 13

*Recommended: Values recommended by the American Malting Barley Association for two-row malting barley. Conrad is a commercial malting barley variety that is routinely included as a standard check during micromalting. S/T, Ratio of soluble to total protein; DP, Diastatic power; AA, Alpha-amylase; WT, Wildtype; FAN, Free Amino Nitrogen. Bolded numbers indicate statistically different values between the mutant and the wildtype (p < 0.05; n = 4).*

## Discussion

Over the past two decades the concept of ROS as important signaling molecules in plants has gained traction (Miller et al., 2018). The identification of the plant NADPH oxidases, homologs of the well characterized mammalian gp91phox, was a crucial discovery in recognizing that deliberate generation of ROS plays a pivotal role during plant development and stress adaptation (Mahalingam and Fedoroff, 2003). In the model plant Arabidopsis, 10 genes constitute the RBOH gene family (Torres and Dangl, 2005) and similar numbers of RBOH genes were reported in several other plants including rice, brassica and soybeans (Torres and Dangl, 2005; Wang et al., 2013; Li et al., 2019; Liu et al., 2019). Using the domain-based search of protein sequences in the PFAM database we have identified seven novel genes in addition to the six previously reported (Lightfoot et al., 2008), bringing the repertoire of RBOH genes to 13 in the barley genome.

Phylogenetic analysis of plant RBOHs (Figure 1) showing distinct grouping of monocot and dicot clades is consistent with a larger study using 127 RBOH sequences from 26 different plant species (Kaur et al., 2018). The first identified barley RBOH, HvRBOHA was found to be closely related to the rice OsRBOHA (Trujillo et al., 2006). Based on these earlier studies and the current phylogenetic tree, we propose that the naming convention for the barley RBOH genes and other monocot RBOHs should be based on the rice RBOH genes. Thus, the HvRBOHs were renamed with suffixes A-I corresponding to their rice orthologs (Table 1). HvRBOHB closely related to OsRBOHB and HvRBOHI co-orthologous to OsRBOHI, contained two and four closely related genes, respectively, based on their phylogenetic groupings (Figure 1).

In most plant species including barley (Supplementary Figure 3) the distribution of the RBOH gene family members showed some clustering on particular chromosomes, as well as individual gene family members that were localized independently (Marino et al., 2011; Cheng et al., 2013; Wang et al., 2013; Li et al., 2019; Liu et al., 2019). These observations suggest that RBOH gene family members probably underwent duplications in most plant genomes even before the separation into eudicots and monocots. Whole genome duplication events have been reported in both the monocot and dicot lineages (Barker et al., 2009; Jiao et al., 2014). The clustered as well as dispersed chromosomal localization patterns of HvRBOH genes suggest that apart from ancient duplication events several members of this gene family in barley have also continued to evolve independently.

Another important mechanism that may be operative in the functional divergence of HvRBOHs is through alternative splicing. The 13 HvRBOH genes can theoretically produce more than 200 different proteins based on predicted alternate splice variants (Table 1). In Arabidopsis, AtRBOHB gene has been shown to impact after-ripening and germination via alternatively spliced regulation of this gene by hormones and environment (Muller et al., 2009a). In maize, ZmRBOHB produces two splice variants that show differential accumulation in response to various abiotic stresses (Lin et al., 2009b). However, expression of splice variant isoforms has not yet been demonstrated for any of the barley RBOH genes and warrants further analysis.

Plant RBOH proteins including the barley sequences identified in this study contain the characteristic calcium binding EF-hand motif in their N terminal region which are known to play a key role in their regulation (Sagi and Fluhr, 2001; Wong et al., 2007; Takeda et al., 2008; Figure 2). In fact, of the 14 barley genes identified in the *in silico* analysis with NADPH oxidase domain (Supplementary Text 1), one gene did not have the EF-hand motif and hence was not considered for further analysis. As a plasma membrane localized protein, RBOHs have six transmembrane domains (Keller et al., 1998; Cheng et al., 2013), a pair of conserved histidine residues in TM3 and TM5 crucial for heme-binding during the electron transfer process (Finegold et al., 1996), a conserved histidine in the FAD binding domain that affect expression (Yoshida et al., 1998; Figure 2). A proline residue in the NAD(P)H-ribose binding domain, an aspartate residue flanking the NAD(P)H-ribose and NAD(P)H-adenine crucial for catalytic activity of the gp91Phox (Segal et al., 1992; Vignais, 2002), a redox-sensitive cysteine in the C-terminus in the NAD(P)H adenine binding domain (Yun et al., 2011) were found to be conserved in 12 of the 13 barley RBOH sequences.

Interestingly, the pattern of the intron and exons were consistent with the phylogenetic groupings of the barley RBOHs constructed using protein sequences (Figures 3, 4). Most of the variation in the gene size was attributable to the observed variation within introns. Based on these observations we speculate the barley RBOH gene family continued to evolve independently leading to functional divergence and/or emergence of new functions.

Given that regulated production of ROS is a requirement for normal developmental processes it was not surprising that RBOH genes exhibited varying levels of expression in the different tissues (Figure 5). There were several similarities between the spatial expression patterns of HvRBOH genes and those from other plant species. For example, in Arabidopsis and Rice, RBOH orthologs AtRBOHF (Torres et al., 1998; Sagi and Fluhr, 2001) and OsRBOHA and OsRBOHC (Kaur and Pati, 2016), respectively, were found to be expressed in all tissues/organ examined and our investigation revealed the two barley orthologs (HvRBOHA and HvRBOHC) also share the same expression pattern. The barley HvRBOHD and its ortholog in rice OsRBOHD and Arabidopsis AtRBOHH are expressed strongly in the flower tissues (Kaur and Pati, 2016). AtRBOHE is expressed preferentially in roots and seed tissues (Sagi and Fluhr, 2006), and the barley ortholog HvRBOHG is strongly expressed in roots, head and coleoptile tissues (Lightfoot et al., 2008), while the closely related grape VvrbohE is highly expressed in roots, ovules and inflorescences (Cheng et al., 2013). It therefore seems that members belonging to specific RBOH clades from different plant species have similar expression signatures suggesting that there may be conserved functionality amongst members of the same group. It is important to note that genes with low levels of expression such as the HvRBOHB1, HvRBOHI1, HvRBOHI2, and the HvRBOHF may be expressed at very specific developmental stages, as has been shown for their rice orthologs, OsRBOHI expressed during heading and OsRBOHF during stem elongation stage (Kaur and Pati, 2016).

Several studies have reported on the role of ROS produced by NADPH oxidases in regular seed germination in barley (Ishibashi et al., 2010, 2015). However, the role of these genes during the actual malting process has not been investigated. This study showed that nine of the 13 HvRBOH genes significantly modulated their expression profiles during the various malting stages (Figure 6). Consistent with the changes in the expression of HvRBOHs during the malting process, ROS accumulation was significantly higher during steep and early stages of germination (Figure 7A). This was attributable to both superoxide and hydrogen peroxide (Figure 7B) and is similar to the ROS localization patterns reported for laboratory germination (Ishibashi et al., 2015). Based on the HvRBOH gene expression patterns, ROS quantification and tissue localization it is tempting to speculate HvRBOH-induced ROS is intimately associated with the malting process.

In the mature Arabidopsis seeds, carbonylation mainly affected 12S cruciferins, the major seed storage proteins, that disappeared steadily, reflecting their mobilization during plantlet establishment and thus demonstrating that protein oxidation during germination is a selective phenomenon (Job et al., 2005). Dormancy alleviation in sunflower seeds was associated with ROS production and targeted changes in protein carbonylation (Oracz et al., 2007). The observed increase in the carbonylation of barley proteins during malting is concordant with the increasing ROS levels observed during steep and early stages of germination (Figures 8A,B). It will be interesting to determine the precise identity of the individual proteins undergoing carbonylation during the malting process.

In a previous study, changes in lipid content during barley malting reported a slight decline during steep, followed by restoration to normal levels by end of germination and a nearly 70% reduction by the end of kilning stage (Kaukovirta-Norja and Laasko, 1993). Consistent with this pattern there was also a significant reduction in the oxidized lipid content at the end of steep and 1 DAG (Figure 8C). However, our results indicate that oxidized lipids in the kilned sample is nearly twofold higher compared to the barley seeds. It has been reported that the lipoxygenase enzyme activity that increase during germination is greatly reduced (>95%) due to inactivation at high temperatures during kilning cycle (Yang and Schwarz, 1995). Based on these observations, we speculate that the higher levels of lipid oxidation in kilned samples is likely associated with the presence of oxygen with elevated temperatures required for kilning (Figure 8C). Thus, lipid peroxidation may be the source of the higher ROS levels detected in the kilned seeds but warrants further investigation since oxidized lipids can have a bearing on beer flavor and shelf life/stability (Bamforth et al., 1993).

Our observation that ROS generated during the malting process can lead to oxidation of proteins and lipids prompted us to examine if there are concomitant changes in the antioxidants. Recent reports indicate that the overall antioxidant capacity of malt increases during malting as a consequence of an increase of phenolic compounds (Carvalho et al., 2016). Kilning increases the antioxidative capacity of the malt due to generation of polyphenolic compounds (Lu et al., 2007) and Malliard reaction products (MRPs) that are known to have antiradical and antioxidant properties (Maillard and Berset, 1995; Woffenden et al., 2001; Papetti et al., 2006; Figure 8D). We speculate that the higher levels of both the tocopherols and tocotrienols observed in the kilned samples (Figure 8E and Supplementary Figure 4) may be induced in response to high temperatures (heat stress) (Roach et al., 2018) during the kilning cycle and an important factor contributing to the overall antioxidant capacity of the malt.

To make a direct correlation between expression of RBOHs and ROS often NADPH oxidase inhibitors such as DPI has been routinely used in many plant species including barley (Ishibashi et al., 2010, 2015). However, within the context of micromalting, the pretreatment of seeds with inhibitor such as DPI prior to malting, is simply not practical. Rather, we examined this correlation using the RNAi line of the HvRBOHC (previously referred to as HvRBOHF2) gene, that also exhibited significant reduction in the levels of its related gene HvRBOHA (previously referred to as HvRBOHF1) (Trujillo et al., 2006). While the anticipated reduction in expression of HvRBOHA/C was observed, it was accompanied by compensatory increases in the expression of other HvRBOH genes, most notably the HvRBOHH during steeping and other gene family members during germination stage (Figure 9). Genetic compensation was phenotypically confirmed by ROS staining of seeds from the knockdown lines from various malting stages that were similar to those observed in the varietal types (Conrad and Golden Promise) (Supplementary Figure 5).

It is important to point out that our analysis using whole seeds cannot extricate the subtle differences in the temporal and spatial expression patterns of HvRBOHs that could play a crucial role during malting. Some of these subtleties manifested physiologically in the quality of the malts generated from the HvRBOHA/C RNAi transgenic line which was suboptimal compared to the standard check (Table 2). For instance, we observed that genetic downregulation of HvRBOHA/C, led to a significant reduction in the alpha-amylase activity. It has been reported ROS produced by NADPH oxidases promote GA biosynthesis in embryos, that in turn induces and activates NADPH oxidases in aleurone cells, and ROS produced by NADPH oxidases induce α-amylase (Ishibashi et al., 2015). Reduced alpha-amylase activity can retard the degradation of endosperm cell wall (Muller et al., 2009b) that can lead to excessive beta glucan content and in turn decrease the extract content in the wort (Bamforth, 2003, 2017). In line with these studies the beta glucan content from the malted seeds of RNAi transgenic HvRBOHA/C line was significantly higher compared with the standard check and the malt extractability was also greatly reduced (Table 2). Cell specific expression of HvRBOHs including barley varieties with divergent malting attributes will aid in teasing out the intricate connections between HvRBOHs, ROS and its influence on this complex commercially important trait.

## Data Availability Statement

The original contributions presented in the study are included in the article/Supplementary Material, further inquiries can be directed to the corresponding author/s.

## Author Contributions

RM conceived all the experiments, conducted the bioinformatic analysis for identifying HvRBOHs, phylogenetic analysis, carbonylation assays, ROS analysis, and wrote the manuscript. DG conducted the histochemical assays, qPCR experiments, lipid peroxidation, and wrote sections of the manuscript related to these experiments. JW conducted the malt quality analysis of the transgenic barley lines and edited the manuscript. All authors contributed to the article and approved the submitted version.

## Conflict of Interest

The authors declare that the research was conducted in the absence of any commercial or financial relationships that could be construed as a potential conflict of interest.

## Footnotes

1 http://www.heatmapper.ca/expression/

## Supplementary Material

Supplementary Figure 1 Coomassie stained gel of protein samples used for identifying carbonylated proteins.

Supplementary Figure 2 A graphical representation of the distribution of NADPH oxidase family across species (Output from PFAM database).

Supplementary Figure 3 Location of the 13 HvRBOH genes on the barley chromosomes. The black triangles represent the HvRBOH genes. The number corresponding to each triangle in this figure corresponds to the numbers in Table 1 that provides details of each gene.

Supplementary Figure 4 Profile of the tocochromanol isoforms during various stages of barley malting. T and T3 refers to tocopherols and tococtrienols, respectively. Error bar represents the standard error (*n* = 4). ^∗^Represents statistically significant changes (*p* < 0.05) in the tocols when compared to dry seeds.

Supplementary Figure 5 Histochemical localization of ROS during barley malting in the seeds of HvRBOHA/C knockdown line and wildtype Golden Promise. The blue staining of the embryos and aleurone layer indicates presence of superoxide and the brownish stain in these tissues shows the presence of hydrogen peroxide at different stages of germination during malting.

Supplementary Table 1 List of primers used for qPCR analysis of the newly identified barley HvRBOH genes.

Supplementary Table 2 Averaged cT values from the real-time PCR analysis of the HvRBOH genes during malting in barley variety Conrad, HvRBOHA/C knockdown line and wildtype Golden Promise.

Supplementary Text 1 Sequences of the barley protein sequences containing the NADPH oxidase domain retrieved from the PFAM database.
