# Supplementary figures and images for: The Barley (Hordeum vulgare ssp. vulgare) Respiratory Burst Oxidase Homolog (HvRBOH) Gene Family and Their Plausible Role on Malting Quality
Source: Front Plant Sci. 2021 Feb 19;12:608541. doi: 10.3389/fpls.2021.608541 (PMC7934426; doi:10.3389/fpls.2021.608541)

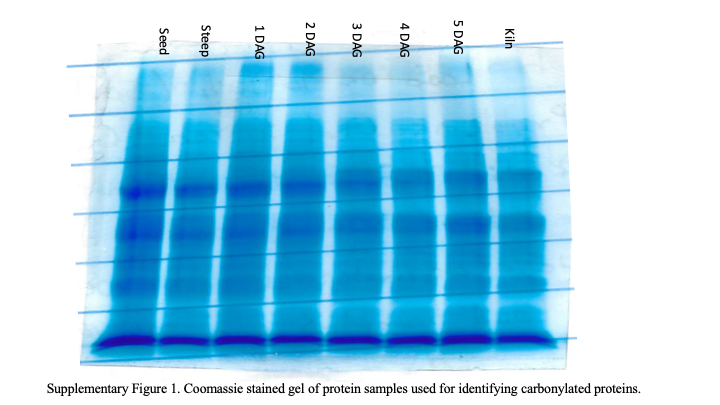

Supplement: Supplementary Figure 1 — Coomassie stained gel of protein samples used for identifying carbonylated proteins. [file Image_1.TIFF]
