# Supplementary material for: The Barley (Hordeum vulgare ssp. vulgare) Respiratory Burst Oxidase Homolog (HvRBOH) Gene Family and Their Plausible Role on Malting Quality
Source: Front Plant Sci. 2021 Feb 19;12:608541. doi: 10.3389/fpls.2021.608541 (PMC7934426; doi:10.3389/fpls.2021.608541)
Supplement: Supplementary Figure 2 — A graphical representation of the distribution of NADPH oxidase family across species (Output from PFAM database). [file Image_2.pdf]

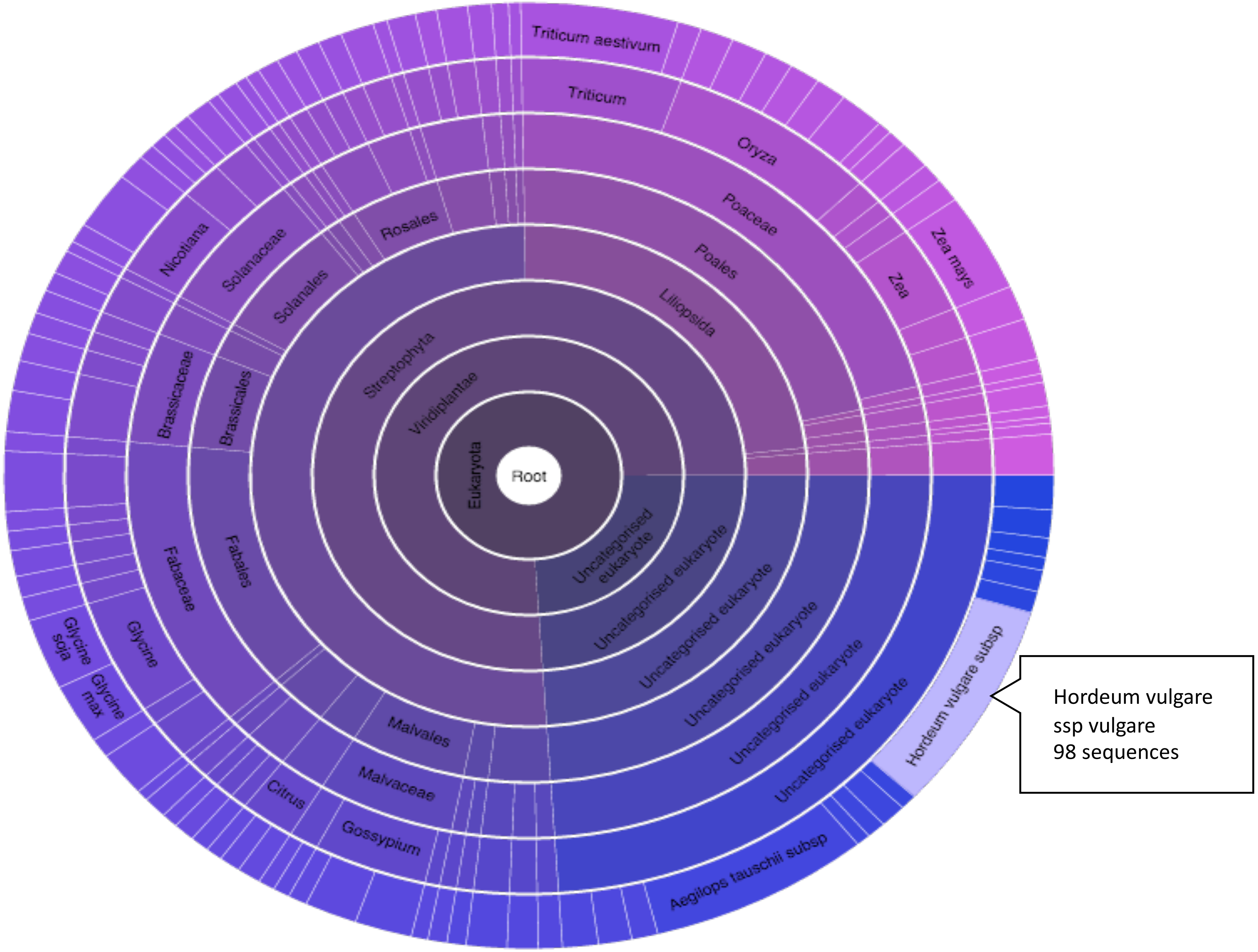

Supplementary Figure 2. A graphical representation of the distribution of NADPH oxidase family across species. (From PFAM database)
