# Supplementary material for: The Barley (Hordeum vulgare ssp. vulgare) Respiratory Burst Oxidase Homolog (HvRBOH) Gene Family and Their Plausible Role on Malting Quality
Source: Front Plant Sci. 2021 Feb 19;12:608541. doi: 10.3389/fpls.2021.608541 (PMC7934426; doi:10.3389/fpls.2021.608541)
Supplement: Supplementary Figure 3 — Location of the 13 HvRBOH genes on the barley chromosomes. The black triangles represent the HvRBOH genes. The number corresponding to each triangle in this figure corresponds to the numbers in Table 1 that provides details of each gene. [file Image_3.pdf]

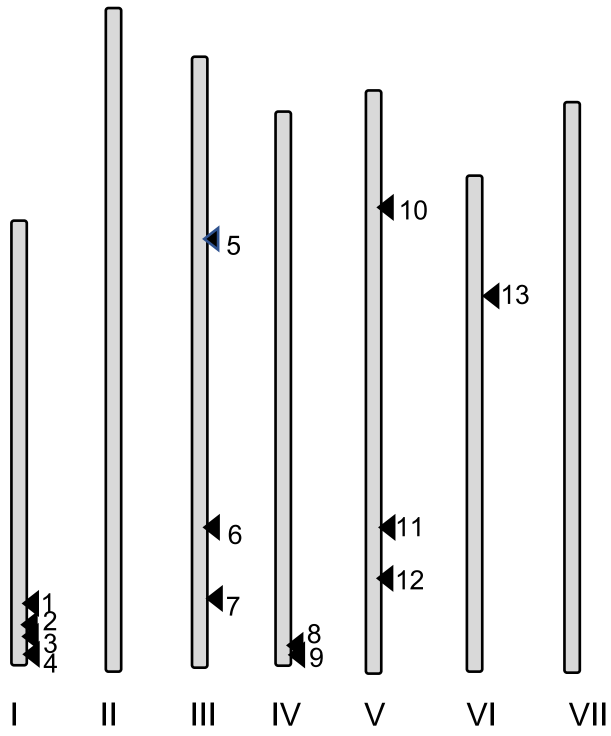

Supplementary Figure 3. Location of the 13 HvRBOH genes on the barley chromosomes. The black triangles represent the HvRBOH genes. The number corresponding to each triangle in this figure corresponds to the numbers in Table 1 that provides details of each gene.
