# Supplementary material for: The Barley (Hordeum vulgare ssp. vulgare) Respiratory Burst Oxidase Homolog (HvRBOH) Gene Family and Their Plausible Role on Malting Quality
Source: Front Plant Sci. 2021 Feb 19;12:608541. doi: 10.3389/fpls.2021.608541 (PMC7934426; doi:10.3389/fpls.2021.608541)
Supplement: Supplementary Figure 4 — Profile of the tocochromanol isoforms during various stages of barley malting. T and T3 refers to tocopherols and tococtrienols, respectively. Error bar represents the standard error (n = 4). ∗Represents statistically significant changes (p < 0.05) in the tocols when compared to dry seeds. [file Image_4.pdf]

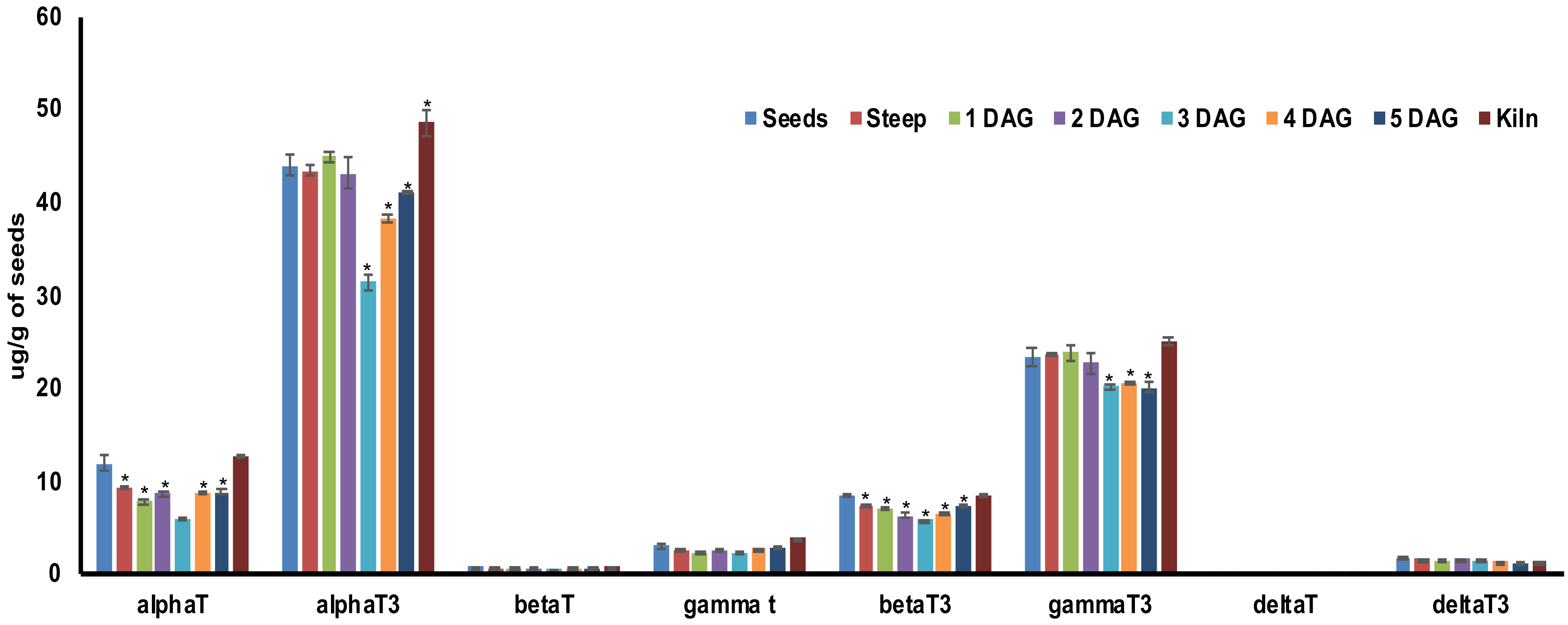

Supplementary Figure 4. Profile of the eight tocochromanol isoforms during various stages of barley malting in cultivar Conrad
