# Supplementary material for: The Barley (Hordeum vulgare ssp. vulgare) Respiratory Burst Oxidase Homolog (HvRBOH) Gene Family and Their Plausible Role on Malting Quality
Source: Front Plant Sci. 2021 Feb 19;12:608541. doi: 10.3389/fpls.2021.608541 (PMC7934426; doi:10.3389/fpls.2021.608541)
Supplement: Supplementary Figure 5 — Histochemical localization of ROS during barley malting in the seeds of HvRBOHA/C knockdown line and wildtype Golden Promise. The blue staining of the embryos and aleurone layer indicates presence of superoxide and the brownish stain in these tissues shows the presence of hydrogen peroxide at different stages of germination during malting. [file Image_5.pdf]

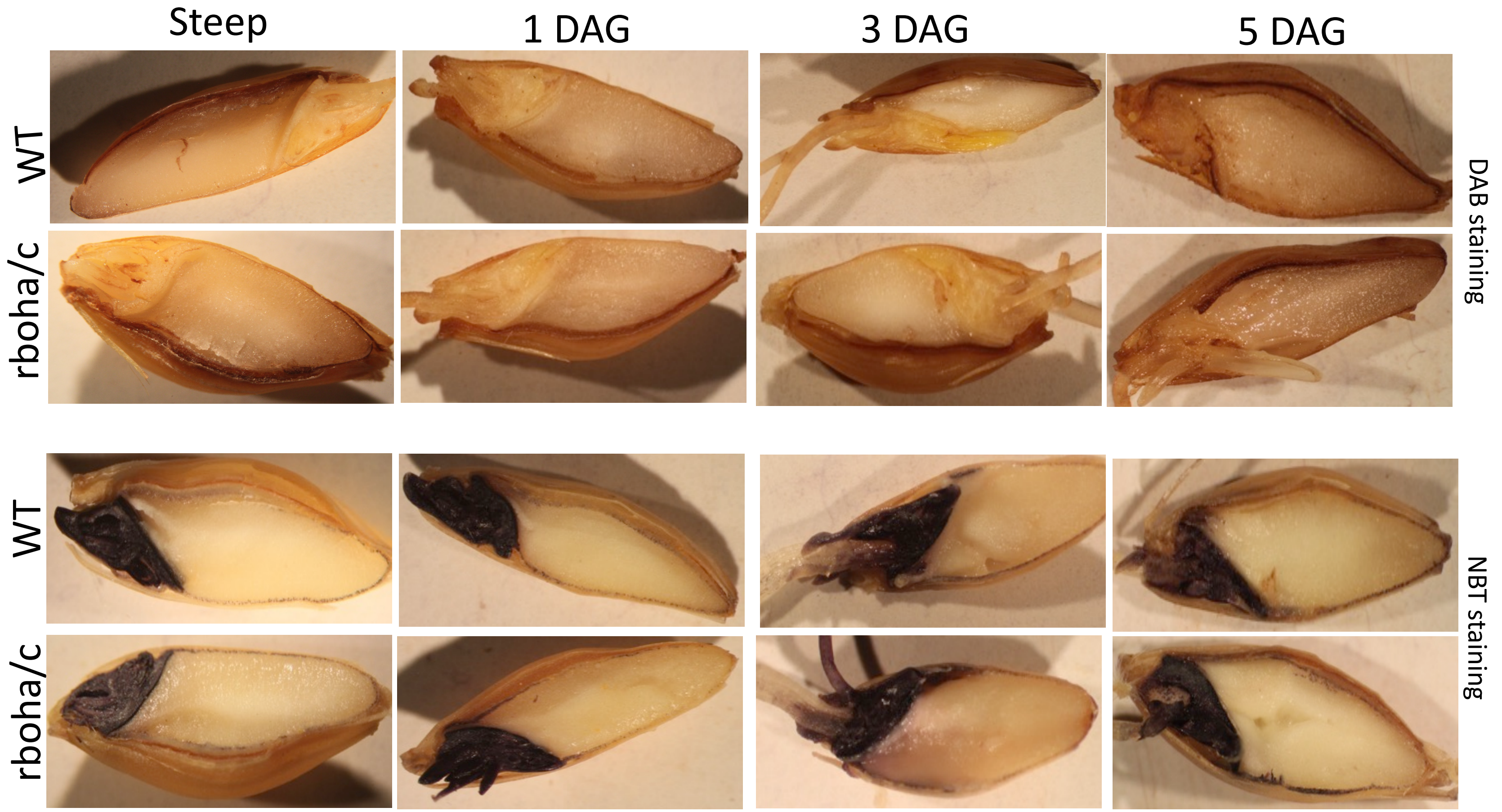

Supplementary Figure 5. Histochemical localization of ROS during barley malting in the seeds of HvRBOHA/C knockdown line and wildtype Golden Promise. The blue staining of the embryos and aleurone layer indicates presence of superoxide and the brownish stain in these tissues shows the presence of hydrogen peroxide at different stages of germination during malting.
