# Supplementary material for: The Barley (Hordeum vulgare ssp. vulgare) Respiratory Burst Oxidase Homolog (HvRBOH) Gene Family and Their Plausible Role on Malting Quality
Source: Front Plant Sci. 2021 Feb 19;12:608541. doi: 10.3389/fpls.2021.608541 (PMC7934426; doi:10.3389/fpls.2021.608541)
Supplement: Supplementary Table 1 — List of primers used for qPCR analysis of the newly identified barley HvRBOH genes. [file Data_Sheet_1.PDF]

Supplementary Table 1. List of primer sequences used for real-time PCR analysis of the new RBOH genes identified in this study.

**Ensembl ID**	**Primer name**	**Primer sequence**
HORVU5Hr1G024550	5633_F	GCGTCGGGGTCTTCTACTG
	5633_R	TGTGGAAGACGAACTTGGTG
HORVU4Hr1G081670	55429_F	GAGCAAGGCTCCTTTGAGTG
	55429_R	CCGTCCTCGTAGACACTGGT
HORVU1Hr1G071340	11469_F	GAAGGCCTGTGAAGCAGAAG
	11469_R	GAAAGGGCCATCAACAAAGA
HORVU1Hr1G072160	53927_F	GTGCGATCGGTGAAGAAGAT
	53927_R	GTACTGCCCGCTCTTGTACC
HORVU5Hr1G078630	73746_F	ATGCTCCAGTCCCTCAACC
	73746_R	GGCGATGTCCTTGTACACCT
HORVU1Hr1G072140	69263_F	GCTCGTTAATGCCTTTCTCG
	69263_R	GTAGAGAAAGTGCCCGTGGA
HORVU6Hr1G035970	77866_F	AGGGTCAGGACACATTTTGC
	77866_R	CTTTCGCTAGTGTGGGCTTC
