# Supplementary material for: The Barley (Hordeum vulgare ssp. vulgare) Respiratory Burst Oxidase Homolog (HvRBOH) Gene Family and Their Plausible Role on Malting Quality
Source: Front Plant Sci. 2021 Feb 19;12:608541. doi: 10.3389/fpls.2021.608541 (PMC7934426; doi:10.3389/fpls.2021.608541)
Supplement: Supplementary file 8 [file Data_Sheet_3.PDF]

HvRBOH genes in HvRBOHA/C knockdown and Golden Promise

Stage	GeneID	Mean	SD	G3P mean	G3P SD	dcT	ddcT	g2-Fold char	SE of Gene	E of GAPDI	SE for Fold
F1 Seed	HvRBOHB1	28.05	0.22	21.61	0.28	6.43	0.00	1.00	0.13	0.08	0.13
F1 Steep	HvRBOHB1	28.85	0.09	21.61	0.28	7.24	0.80	0.57	0.05	0.08	0.06
F1 1 DAG	HvRBOHB1	27.78	0.16	21.61	0.28	6.17	-0.26	1.20	0.09	0.08	0.10
F1 2 DAG	HvRBOHB1	28.65	0.33	21.61	0.28	7.03	0.60	0.66	0.19	0.08	0.20
F1 3 DAG	HvRBOHB1	27.99	0.21	21.61	0.28	6.38	-0.06	1.04	0.12	0.08	0.13
F1 4 DAG	HvRBOHB1	29.11	0.25	21.61	0.28	7.50	1.07	0.48	0.15	0.08	0.15
F1 5 DAG	HvRBOHB1	29.46	0.43	21.61	0.28	7.85	1.42	0.37	0.25	0.08	0.25
F1 Seed	HvRBOHG	27.88	0.12	21.61	0.28	6.27	0.00	1.00	0.07	0.08	0.07
F1 Steep	HvRBOHG	25.30	0.17	21.61	0.28	3.69	-2.58	5.99	0.10	0.08	0.11
F1 1 DAG	HvRBOHG	26.14	0.10	21.61	0.28	4.52	-1.75	3.36	0.06	0.08	0.06
F1 2 DAG	HvRBOHG	26.78	0.17	21.61	0.28	5.16	-1.11	2.16	0.10	0.08	0.11
F1 3 DAG	HvRBOHG	26.25	0.12	21.61	0.28	4.63	-1.64	3.11	0.07	0.08	0.08
F1 4 DAG	HvRBOHG	27.79	0.08	21.61	0.28	6.18	-0.09	1.07	0.05	0.08	0.05
F1 5 DAG	HvRBOHG	28.65	0.23	21.61	0.28	7.04	0.77	0.59	0.13	0.08	0.14
F1 Seed	HvRBOHC	30.84	0.41	21.61	0.28	9.22	0.00	1.00	0.24	0.08	0.24
F1 Steep	HvRBOHC	29.91	0.20	21.61	0.28	8.29	-0.93	1.90	0.12	0.08	0.12
F1 1 DAG	HvRBOHC	28.71	0.08	21.61	0.28	7.10	-2.12	4.35	0.04	0.08	0.05
F1 2 DAG	HvRBOHC	29.53	0.31	21.61	0.28	7.91	-1.31	2.47	0.18	0.08	0.18
F1 3 DAG	HvRBOHC	28.87	0.09	21.61	0.28	7.26	-1.96	3.89	0.05	0.08	0.06
F1 4 DAG	HvRBOHC	29.85	0.08	21.61	0.28	8.23	-0.99	1.98	0.05	0.08	0.05
F1 5 DAG	HvRBOHC	30.73	0.25	21.61	0.28	9.11	-0.11	1.08	0.14	0.08	0.15
F1 Seed	HvRBOHD	28.64	0.23	21.61	0.28	7.03	0.00	1.00	0.13	0.08	0.14
F1 Steep	HvRBOHD	28.09	0.12	21.61	0.28	6.48	-0.55	1.47	0.07	0.08	0.07
F1 1 DAG	HvRBOHD	28.36	0.22	21.61	0.28	6.74	-0.29	1.22	0.12	0.08	0.13
F1 2 DAG	HvRBOHD	29.87	0.78	21.61	0.28	8.26	1.23	0.43	0.45	0.08	0.46
F1 3 DAG	HvRBOHD	29.06	0.36	21.61	0.28	7.45	0.42	0.75	0.21	0.08	0.22
F1 4 DAG	HvRBOHD	29.91	0.46	21.61	0.28	8.30	1.27	0.42	0.26	0.08	0.27
F1 5 DAG	HvRBOHD	30.65	0.55	21.61	0.28	9.04	2.01	0.25	0.32	0.08	0.32
F1 Seed	HvRBOHI2	29.44	0.05	21.61	0.28	7.83	0.00	1.00	0.03	0.08	0.04
F1 Steep	HvRBOHI2	30.49	0.22	21.61	0.28	8.88	1.05	0.48	0.13	0.08	0.13
F1 1 DAG	HvRBOHI2	29.55	0.33	21.61	0.28	7.94	0.11	0.93	0.19	0.08	0.20
F1 2 DAG	HvRBOHI2	30.31	0.05	21.61	0.28	8.70	0.87	0.55	0.03	0.08	0.04
F1 3 DAG	HvRBOHI2	30.59	0.27	21.61	0.28	8.98	1.15	0.45	0.15	0.08	0.16
F1 4 DAG	HvRBOHI2	31.33	0.15	21.61	0.28	9.71	1.88	0.27	0.08	0.08	0.09
F1 5 DAG	HvRBOHI2	31.81	0.33	21.61	0.28	10.20	2.37	0.19	0.19	0.08	0.19
F1 Seed	HvRBOHF	27.80	0.11	21.61	0.28	6.19	0.00	1.00	0.06	0.08	0.07
F1 Steep	HvRBOHF	29.11	0.19	21.61	0.28	7.50	1.31	0.40	0.11	0.08	0.11
F1 1 DAG	HvRBOHF	28.36	0.60	21.61	0.28	6.75	0.56	0.68	0.35	0.08	0.35
F1 2 DAG	HvRBOHF	28.55	0.06	21.61	0.28	6.94	0.75	0.59	0.04	0.08	0.04
F1 3 DAG	HvRBOHF	28.77	0.13	21.61	0.28	7.15	0.96	0.51	0.07	0.08	0.08
F1 4 DAG	HvRBOHF	29.99	0.50	21.61	0.28	8.38	2.19	0.22	0.29	0.08	0.29
F1 5 DAG	HvRBOHF	29.70	0.37	21.61	0.28	8.09	1.90	0.27	0.21	0.08	0.22
F1 Seed	HvRBOHB2	27.89	0.06	21.61	0.28	6.28	0.00	1.00	0.04	0.08	0.04
F1 Steep	HvRBOHB2	26.61	0.12	21.61	0.28	5.00	-1.28	2.44	0.07	0.08	0.08
F1 1 DAG	HvRBOHB2	24.85	0.05	21.61	0.28	3.24	-3.04	8.23	0.03	0.08	0.03
F1 2 DAG	HvRBOHB2	24.97	0.05	21.61	0.28	3.36	-2.92	7.57	0.03	0.08	0.04
F1 3 DAG	HvRBOHB2	24.61	0.08	21.61	0.28	3.00	-3.28	9.74	0.05	0.08	0.05
F1 4 DAG	HvRBOHB2	26.08	0.11	21.61	0.28	4.46	-1.82	3.52	0.06	0.08	0.07
F1 5 DAG	HvRBOHB2	26.67	0.08	21.61	0.28	5.05	-1.23	2.34	0.04	0.08	0.05
F1 Seed	HvRBOHA	30.18	0.26	21.61	0.28	8.57	0.00	1.00	0.15	0.08	0.16
F1 Steep	HvRBOHA	30.94	0.54	21.61	0.28	9.32	0.75	0.59	0.31	0.08	0.32
F1 1 DAG	HvRBOHA	28.55	0.19	21.61	0.28	6.94	-1.63	3.10	0.11	0.08	0.11
F1 2 DAG	HvRBOHA	28.84	0.09	21.61	0.28	7.23	-1.34	2.53	0.05	0.08	0.06
F1 3 DAG	HvRBOHA	28.66	0.07	21.61	0.28	7.05	-1.52	2.87	0.04	0.08	0.05
F1 4 DAG	HvRBOHA	30.07	0.23	21.61	0.28	8.46	-0.11	1.08	0.13	0.08	0.14
F1 5 DAG	HvRBOHA	30.80	0.50	21.61	0.28	9.19	0.62	0.65	0.29	0.08	0.29
F1 Seed	HvRBOHE	30.22	0.45	21.61	0.28	8.61	0.00	1.00	0.26	0.08	0.27
F1 Steep	HvRBOHE	29.31	0.05	21.61	0.28	7.69	-0.92	1.89	0.03	0.08	0.04
F1 1 DAG	HvRBOHE	29.17	0.18	21.61	0.28	7.56	-1.05	2.07	0.10	0.08	0.11
F1 2 DAG	HvRBOHE	29.41	0.13	21.61	0.28	7.79	-0.82	1.76	0.07	0.08	0.08
F1 3 DAG	HvRBOHE	28.76	0.07	21.61	0.28	7.14	-1.47	2.76	0.04	0.08	0.05
F1 4 DAG	HvRBOHE	28.97	0.06	21.61	0.28	7.36	-1.25	2.39	0.03	0.08	0.04
F1 5 DAG	HvRBOHE	29.07	0.25	21.61	0.28	7.46	-1.15	2.22	0.14	0.08	0.15
F1 Seed	HvRBOHI1	28.43	0.31	21.61	0.28	6.82	0.00	1.00	0.18	0.08	0.19
F1 Steep	HvRBOHI1	28.88	0.07	21.61	0.28	7.27	0.45	0.73	0.04	0.08	0.04
F1 1 DAG	HvRBOHI1	29.33	0.16	21.61	0.28	7.71	0.89	0.54	0.09	0.08	0.10
F1 2 DAG	HvRBOHI1	29.81	0.18	21.61	0.28	8.19	1.37	0.39	0.10	0.08	0.11
F1 3 DAG	HvRBOHI1	29.31	0.09	21.61	0.28	7.70	0.88	0.54	0.05	0.08	0.06
F1 4 DAG	HvRBOHI1	30.48	0.32	21.61	0.28	8.87	2.05	0.24	0.18	0.08	0.19
F1 5 DAG	HvRBOHI1	30.88	0.26	21.61	0.28	9.27	2.45	0.18	0.15	0.08	0.16
F1 Seed	HvRBOHI3	27.85	0.09	21.61	0.28	6.23	0.00	1.00	0.05	0.08	0.06
F1 Steep	HvRBOHI3	26.02	0.10	21.61	0.28	4.41	-1.82	3.53	0.06	0.08	0.06
F1 1 DAG	HvRBOHI3	25.44	0.08	21.61	0.28	3.83	-2.40	5.28	0.04	0.08	0.05
F1 2 DAG	HvRBOHI3	26.61	0.07	21.61	0.28	5.00	-1.23	2.35	0.04	0.08	0.05
F1 3 DAG	HvRBOHI3	26.48	0.14	21.61	0.28	4.87	-1.36	2.57	0.08	0.08	0.09
F1 4 DAG	HvRBOHI3	27.96	0.02	21.61	0.28	6.34	0.11	0.93	0.01	0.08	0.02
F1 5 DAG	HvRBOHI3	28.84	0.23	21.61	0.28	7.22	0.99	0.50	0.13	0.08	0.14
F1 Seed	HvRBOHI4	29.30	0.10	21.61	0.28	7.69	0.00	1.00	0.06	0.08	0.06
F1 Steep	HvRBOHI4	29.12	0.15	21.61	0.28	7.50	-0.19	1.14	0.09	0.08	0.09
F1 1 DAG	HvRBOHI4	27.73	0.02	21.61	0.28	6.11	-1.58	2.99	0.01	0.08	0.02
F1 2 DAG	HvRBOHI4	28.85	0.08	21.61	0.28	7.24	-0.45	1.37	0.04	0.08	0.05
F1 3 DAG	HvRBOHI4	28.82	0.09	21.61	0.28	7.20	-0.49	1.40	0.05	0.08	0.06
F1 4 DAG	HvRBOHI4	30.49	0.21	21.61	0.28	8.88	1.19	0.44	0.12	0.08	0.12
F1 5 DAG	HvRBOHI4	31.24	0.13	21.61	0.28	9.63	1.94	0.26	0.07	0.08	0.08
F1 Seed	HvRBOHH	29.64	0.25	21.61	0.28	8.03	0.00	1.00	0.14	0.08	0.15
F1 Steep	HvRBOHH	23.90	0.04	21.61	0.28	2.29	-5.74	53.63	0.02	0.08	0.03
F1 1 DAG	HvRBOHH	28.36	0.17	21.61	0.28	6.74	-1.29	2.44	0.10	0.08	0.11
F1 2 DAG	HvRBOHH	28.88	0.14	21.61	0.28	7.26	-0.77	1.70	0.08	0.08	0.09
F1 3 DAG	HvRBOHH	29.68	0.11	21.61	0.28	8.07	0.04	0.98	0.07	0.08	0.07
F1 4 DAG	HvRBOHH	31.09	0.13	21.61	0.28	9.47	1.44	0.37	0.07	0.08	0.08
F1 5 DAG	HvRBOHH	29.57	0.09	21.61	0.28	7.96	-0.07	1.05	0.05	0.08	0.06

Stage	GeneID	Mean	SD	G3P mean	G3P SD	dcT	ddcT	g2-Fold char	SE of Gene	E of GAPDI	SE for Fold
WT seed	HvRBOHB1	28.63	0.40	20.65	0.18	7.98	0.00	1.00	0.23	0.05	0.23
Wt Steep	HvRBOHB1	27.46	0.17	20.65	0.18	6.81	-1.17	2.25	0.10	0.05	0.10
WT 1DAG	HvRBOHB1	27.90	0.24	20.65	0.18	7.26	-0.72	1.65	0.14	0.05	0.14
WT 2 DAG	HvRBOHB1	28.16	0.26	20.65	0.18	7.51	-0.47	1.38	0.15	0.05	0.15
WT 3 DAG	HvRBOHB1	27.69	1.02	20.65	0.18	7.05	-0.93	1.91	0.59	0.05	0.59
WT 4 DAG	HvRBOHB1	28.34	0.20	20.65	0.18	7.70	-0.28	1.22	0.12	0.05	0.12
WT 5 DAG	HvRBOHB1	28.06	0.09	20.65	0.18	7.41	-0.57	1.48	0.05	0.05	0.05
WT seed	HvRBOHG	27.87	0.07	20.65	0.18	7.22	0.00	1.00	0.04	0.05	0.04
Wt Steep	HvRBOHG	25.44	0.02	20.65	0.18	4.79	-2.43	5.37	0.01	0.05	0.02
WT 1DAG	HvRBOHG	25.19	0.04	20.65	0.18	4.54	-2.68	6.40	0.03	0.05	0.03
WT 2 DAG	HvRBOHG	25.98	0.01	20.65	0.18	5.33	-1.89	3.70	0.00	0.05	0.01
WT 3 DAG	HvRBOHG	26.13	0.17	20.65	0.18	5.48	-1.74	3.33	0.10	0.05	0.10
WT 4 DAG	HvRBOHG	26.43	0.07	20.65	0.18	5.78	-1.44	2.72	0.04	0.05	0.04
WT 5 DAG	HvRBOHG	26.82	0.11	20.65	0.18	6.17	-1.05	2.07	0.06	0.05	0.07
WT seed	HvRBOHC	29.91	0.65	20.65	0.18	9.26	0.00	1.00	0.37	0.05	0.38
Wt Steep	HvRBOHC	27.38	0.14	20.65	0.18	6.73	-2.53	5.76	0.08	0.05	0.08
WT 1DAG	HvRBOHC	26.21	0.04	20.65	0.18	5.57	-3.69	12.95	0.02	0.05	0.02
WT 2 DAG	HvRBOHC	27.14	0.19	20.65	0.18	6.50	-2.76	6.80	0.11	0.05	0.11
WT 3 DAG	HvRBOHC	26.81	0.21	20.65	0.18	6.16	-3.10	8.58	0.12	0.05	0.13
WT 4 DAG	HvRBOHC	27.13	0.12	20.65	0.18	6.49	-2.77	6.84	0.07	0.05	0.07
WT 5 DAG	HvRBOHC	27.43	0.17	20.65	0.18	6.79	-2.47	5.55	0.10	0.05	0.10
WT seed	HvRBOHD	29.22	0.45	20.65	0.18	8.58	0.00	1.00	0.26	0.05	0.27
Wt Steep	HvRBOHD	28.04	0.03	20.65	0.18	7.39	-1.19	2.28	0.02	0.05	0.02
WT 1DAG	HvRBOHD	29.39	0.48	20.65	0.18	8.75	0.17	0.89	0.28	0.05	0.28
WT 2 DAG	HvRBOHD	28.24	0.24	20.65	0.18	7.59	-0.99	1.98	0.14	0.05	0.14
WT 3 DAG	HvRBOHD	28.96	0.21	20.65	0.18	8.31	-0.27	1.20	0.12	0.05	0.12
WT 4 DAG	HvRBOHD	29.07	0.16	20.65	0.18	8.42	-0.16	1.11	0.09	0.05	0.09
WT 5 DAG	HvRBOHD	28.76	0.19	20.65	0.18	8.12	-0.46	1.38	0.11	0.05	0.11
WT seed	HvRBOHI2	30.31	0.32	20.65	0.18	9.66	0.00	1.00	0.19	0.05	0.19
Wt Steep	HvRBOHI2	29.72	0.16	20.65	0.18	9.08	-0.58	1.50	0.09	0.05	0.09
WT 1DAG	HvRBOHI2	29.77	0.15	20.65	0.18	9.13	-0.53	1.45	0.09	0.05	0.09
WT 2 DAG	HvRBOHI2	30.16	0.35	20.65	0.18	9.51	-0.15	1.11	0.20	0.05	0.20
WT 3 DAG	HvRBOHI2	30.58	0.37	20.65	0.18	9.94	0.28	0.83	0.21	0.05	0.21
WT 4 DAG	HvRBOHI2	30.81	0.40	20.65	0.18	10.16	0.50	0.71	0.23	0.05	0.23
WT 5 DAG	HvRBOHI2	30.63	0.26	20.65	0.18	9.99	0.33	0.80	0.15	0.05	0.15
WT seed	HvRBOHF	28.18	0.16	20.65	0.18	7.54	0.00	1.00	0.09	0.05	0.09
Wt Steep	HvRBOHF	28.12	0.20	20.65	0.18	7.48	-0.06	1.05	0.12	0.05	0.12
WT 1DAG	HvRBOHF	27.69	0.45	20.65	0.18	7.05	-0.49	1.41	0.26	0.05	0.26
WT 2 DAG	HvRBOHF	27.88	0.08	20.65	0.18	7.23	-0.31	1.24	0.05	0.05	0.05
WT 3 DAG	HvRBOHF	28.14	0.05	20.65	0.18	7.49	-0.05	1.03	0.03	0.05	0.03
WT 4 DAG	HvRBOHF	29.04	0.10	20.65	0.18	8.40	0.86	0.55	0.06	0.05	0.06
WT 5 DAG	HvRBOHF	28.80	0.15	20.65	0.18	8.15	0.61	0.65	0.08	0.05	0.09
WT seed	HvRBOHB2	27.71	0.22	20.65	0.18	7.07	0.00	1.00	0.13	0.05	0.13
Wt Steep	HvRBOHB2	26.23	0.06	20.65	0.18	5.58	-1.49	2.80	0.04	0.05	0.04
WT 1DAG	HvRBOHB2	23.99	0.03	20.65	0.18	3.34	-3.73	13.23	0.02	0.05	0.02
WT 2 DAG	HvRBOHB2	24.27	0.08	20.65	0.18	3.63	-3.44	10.86	0.05	0.05	0.05
WT 3 DAG	HvRBOHB2	24.83	0.09	20.65	0.18	4.18	-2.89	7.42	0.05	0.05	0.05
WT 4 DAG	HvRBOHB2	24.87	0.12	20.65	0.18	4.22	-2.85	7.21	0.07	0.05	0.07
WT 5 DAG	HvRBOHB2	25.21	0.08	20.65	0.18	4.56	-2.51	5.69	0.05	0.05	0.05
WT seed	HvRBOHA	31.65	0.24	20.65	0.18	11.00	0.00	1.00	0.14	0.05	0.14
Wt Steep	HvRBOHA	29.34	0.07	20.65	0.18	8.69	-2.31	4.95	0.04	0.05	0.04
WT 1DAG	HvRBOHA	26.88	0.08	20.65	0.18	6.23	-4.77	27.19	0.05	0.05	0.05
WT 2 DAG	HvRBOHA	27.05	0.09	20.65	0.18	6.41	-4.59	24.13	0.05	0.05	0.05
WT 3 DAG	HvRBOHA	27.58	0.18	20.65	0.18	6.94	-4.06	16.72	0.10	0.05	0.11
WT 4 DAG	HvRBOHA	27.57	0.08	20.65	0.18	6.92	-4.08	16.86	0.05	0.05	0.05
WT 5 DAG	HvRBOHA	27.64	0.10	20.65	0.18	7.00	-4.00	16.03	0.06	0.05	0.06
WT seed	HvRBOHE	29.51	0.23	20.65	0.18	8.86	0.00	1.00	0.14	0.05	0.14
Wt Steep	HvRBOHE	29.55	0.08	20.65	0.18	8.90	0.04	0.97	0.05	0.05	0.05
WT 1DAG	HvRBOHE	28.61	0.19	20.65	0.18	7.96	-0.90	1.86	0.11	0.05	0.11
WT 2 DAG	HvRBOHE	28.88	0.26	20.65	0.18	8.23	-0.63	1.55	0.15	0.05	0.15
WT 3 DAG	HvRBOHE	28.78	0.15	20.65	0.18	8.13	-0.73	1.66	0.08	0.05	0.09
WT 4 DAG	HvRBOHE	28.47	0.18	20.65	0.18	7.82	-1.04	2.05	0.10	0.05	0.10
WT 5 DAG	HvRBOHE	28.45	0.11	20.65	0.18	7.81	-1.05	2.08	0.06	0.05	0.07
WT seed	HvRBOHI1	29.10	0.15	20.65	0.18	8.45	-2.18	4.53	0.09	0.05	0.09
Wt Steep	HvRBOHI1	28.42	0.33	20.65	0.18	7.77	-2.86	7.25	0.19	0.05	0.19
WT 1DAG	HvRBOHI1	29.89	0.15	20.65	0.18	9.24	-1.39	2.62	0.09	0.05	0.09
WT 2 DAG	HvRBOHI1	28.76	0.17	20.65	0.18	8.11	-2.52	5.73	0.10	0.05	0.10
WT 3 DAG	HvRBOHI1	29.35	0.27	20.65	0.18	8.70	-1.93	3.81	0.16	0.05	0.16
WT 4 DAG	HvRBOHI1	30.33	0.41	20.65	0.18	9.68	-0.95	1.93	0.24	0.05	0.24
WT 5 DAG	HvRBOHI1	29.68	0.19	20.65	0.18	9.04	-1.59	3.02	0.11	0.05	0.11
WT seed	HvRBOHI3	26.86	0.06	20.65	0.18	6.21	0.00	1.00	0.03	0.05	0.04
Wt Steep	HvRBOHI3	25.14	0.09	20.65	0.18	4.49	-1.72	3.29	0.05	0.05	0.05
WT 1DAG	HvRBOHI3	24.74	0.06	20.65	0.18	4.10	-2.11	4.33	0.04	0.05	0.04
WT 2 DAG	HvRBOHI3	25.80	0.24	20.65	0.18	5.16	-1.05	2.07	0.14	0.05	0.14
WT 3 DAG	HvRBOHI3	26.10	0.10	20.65	0.18	5.45	-0.76	1.70	0.06	0.05	0.06
WT 4 DAG	HvRBOHI3	26.56	0.14	20.65	0.18	5.91	-0.30	1.23	0.08	0.05	0.09
WT 5 DAG	HvRBOHI3	26.68	0.08	20.65	0.18	6.03	-0.18	1.13	0.05	0.05	0.05
WT seed	HvRBOHI4	30.17	0.06	20.65	0.18	9.53	0.00	1.00	0.04	0.05	0.04
Wt Steep	HvRBOHI4	28.68	0.09	20.65	0.18	8.03	-1.50	2.83	0.05	0.05	0.06
WT 1DAG	HvRBOHI4	27.08	0.10	20.65	0.18	6.44	-3.09	8.54	0.06	0.05	0.06
WT 2 DAG	HvRBOHI4	28.51	0.07	20.65	0.18	7.87	-1.66	3.17	0.04	0.05	0.04
WT 3 DAG	HvRBOHI4	28.94	0.03	20.65	0.18	8.30	-1.23	2.35	0.02	0.05	0.02
WT 4 DAG	HvRBOHI4	29.41	0.18	20.65	0.18	8.76	-0.77	1.71	0.10	0.05	0.10
WT 5 DAG	HvRBOHI4	29.49	0.25	20.65	0.18	8.85	-0.68	1.60	0.14	0.05	0.15
WT seed	HvRBOHH	29.69	0.42	20.65	0.18	9.05	0.00	1.00	0.24	0.05	0.25
Wt Steep	HvRBOHH	25.35	0.04	20.65	0.18	4.71	-4.34	20.32	0.02	0.05	0.03
WT 1DAG	HvRBOHH	28.49	0.13	20.65	0.18	7.85	-1.20	2.30	0.08	0.05	0.08
WT 2 DAG	HvRBOHH	29.30	0.29	20.65	0.18	8.65	-0.40	1.32	0.17	0.05	0.17
WT 3 DAG	HvRBOHH	29.92	0.33	20.65	0.18	9.27	0.22	0.86	0.19	0.05	0.19
WT 4 DAG	HvRBOHH	30.66	0.40	20.65	0.18	10.01	0.96	0.51	0.23	0.05	0.24
WT 5 DAG	HvRBOHH	29.05	0.08	20.65	0.18	8.40	-0.65	1.57	0.05	0.05	0.05
