# Supplementary material for: The Barley (Hordeum vulgare ssp. vulgare) Respiratory Burst Oxidase Homolog (HvRBOH) Gene Family and Their Plausible Role on Malting Quality
Source: Front Plant Sci. 2021 Feb 19;12:608541. doi: 10.3389/fpls.2021.608541 (PMC7934426; doi:10.3389/fpls.2021.608541)
Supplement: Supplementary Text 1 — Sequences of the barley protein sequences containing the NADPH oxidase domain retrieved from the PFAM database. [file Data_Sheet_4.pdf]

Supplementary Text1. Sequences of the 98 barley sequences containing the NADPH Oxidase domain retrieved from the PFAM database search.

>A0A287E0V6.1/5-100 Uncharacterized protein
{ECO:0000313|EnsemblPlants:HORVU0Hr1G013380.6}/ HORVU5Hr1G078630.2
RRPSRIDRSKSAAAHALKGLKFISKPDGWPAVEKRFDELAENGGLLHRSKFGKCIGMK
ENAFAGELFDALARRRDISGDSISKAELLEFWDQISDTSFDSRLQTFFDMVDKDADGRI
TEAEVGEIIRLSAAANDLKKITERIEEYARLIMEELDPDNLGYIELYNLETLLLQAPPTQP
SRGGTTSSRNLSPXXXXXXXXXXXXXXXXXXXXXXXYFLEDNWRRCWVIILWFSICAGL
FAWKFVQYRRRAVFEVMGYCVCVAKGGAETLKFNMALVLLPVCRNTITWLRNRTAA
GRVVPFDDNLNFHKVIAAGITVGAGMHIISHLACDFPRLLHATEEEYEPMKPFFGDVQP
PNYWWFVKGTEGWTGLVMLALMAVAFTLATPWFRRGRVRLPGPLSRLTGFNAFWYT
HHLFIIVYALLIVHGHFLYLTKKWQKKSTWMYVAAPMVLYXXXXXXXXXXXXXXX
XXRWPCTPATCCRCASPSPRGSGARAGSTSSSTAPPCRRSSGTRSPSRRRRTTTTSASTS
GRSATGPGSSRASSPRFAGRRRXXXXXXXXXXXXXXXXPEQLPNGADRRAVRRAGAG
LQAVRRGAAGGAGHRGHAHDLHHQGHHQQHEAARRRHRVRQPQRQERVGGVVPDP
AGLLLLGDAGGRLLRLVPRRHGRGGRERQEGHHRAPQLLHQRVRGRGRPVGAHRHA
PVPQPRQARRRRRLRHPREDALRPPELAQGVQGHRPEARRAASRSVLXXXSESECSTA
ARRC
>A0A287E0X5.1/44-139 Uncharacterized protein
{ECO:0000313|EnsemblPlants:HORVU0Hr1G013380.4} HORVU5Hr1G078630.2
TRSSSYGGRAGGHGGGVLRNASTRIKQVSQELRRIASVKRRPSRIDRSKSAAAHALKGL
KFISKPDGWPAVEKRFDELAENGGLLHRSKFGKCIGMKENAFAGELFDALARRRDISG
DSISKAELLEFWDQISDTSFDSRLQTFFDMVDKDADGRITEAEVGEIIRLSAAANDLKKI
TERIEEYARLIMEELDPDNLGYIELYNLETLLLQAPPTQPXXXXXXXXXXXXXXXXXX
XXXXXXXXXRRASYFLEDNWRRCWVIILWFSICAGLFAWKFVQYRRRAVFEVMGYC
VCVAKGGAETLKFNMALVLLPVCRNTITWLRNRTAAGRVVPFDDNLNFHKVIAAGIT
VGAGMHIISHLACDFPRLLHATEEEYEPMKPFFGDVQPPNYWWFVKGTEGWTGLVML
ALMAVAFTLATPWFRRGRVRLPGPLSRLTGFNAFWYTHHLFIIVYALLIVHGHFLYLTK
KWQKKSTWMYVAAPMVLYXXXXXXXXXXXXXXXXXRWPCTPATCCRCASPSPRGS
GARAGSTSSSTAPPCRRSSGTRSPSRRRRTTTTSASTSGRSATGPGSSRASSPRFAGRRR
XXXXXXXXXXXXXXXXPEQLPNGADRRAVRRAGAGLQAVRRGAAGGAGHRGHAHD
LHHQGHHQQHEAARRRHRVRQPQRQERVGGVVPDPAGLLLLGDAGGRLLRLVPRRH
GRGGRERQEGHHRAPQLLHQRVRGRGRPVGAHRHAPVPQPRQARRRRRLRHPREDAL
RPPELAQGVQGHRPEARRAASRSVLXXXSESECSTAARRC
>A0A287G589.1/215-315 Uncharacterized protein
{ECO:0000313|EnsemblPlants:HORVU1Hr1G071340.11}
SRCYDKPRLPLNAFRKNKKKLGAALLAPTSTHVAQSRLAVQRRAEQRSAGQGRLAWT
ARHGHWCRAGAGASATLSGEAQTKGRREERQGRPALPRALHACRCSATAKIGATPLR
NTRHSSPRFLLPSPLRLPTFQIYVRPPAASHRAVVPSPVRAMASSSEGYVDVPLGGEQPP
QQQQQQHSGPVMRKQPSRLASGMKRLASRVTSIRVPDSVMGLKRTHSSAQPALKGLR
FLDKTAAGKDGWKSVEKRFDEMSADGRLHQENFAKCIGMADSKEFAGEVFVAMARR
RKIEPDQGITKEQLKEFWEEMSDNNFDARLRIFFDMCDKNGDGKLTEDEVKEIIVLSAS
ANKLANLKKHAATYASLIMEELDPDGRGHIEVPGNE
>A0A287G590.1/214-314 Uncharacterized protein
{ECO:0000313|EnsemblPlants:HORVU1Hr1G071340.13}

RCYDKPRLPLNAFRKNKKKLGAALLAPTSTHVAQSRLAVQRRAEQRSAGQGRLAWT
ARHGHWCRAGAGASATLSGEAQTKGRREERQGRPALPRALHACRCSATAKIGATPLR
NTRHSSPRFLLPSPLRLPTFQIYVRPPAASHRAVVPSPVRAMASSSEGYVDVPLGGEQPP
QQQQQQHSGPVMRKQPSRLASGMKRLASRVTSIRVPDSVMGLKRTHSSAQPALKGLR
FLDKTAAGKDGWKSVEKRFDEMSADGRLHQENFAKCIGMADSKEFAGEVFVAMARR
RKIEPDQGITKEQLKEFWEEMSDNNFDARLRIFFDMCDKNGDGKLTEDEVKEVSKQTT
VLGSACQTLQISEFSFHAQIIVLSASANKLANLKKHAATYASLIMEELDPDGRGHIEIWQ
LEKLLRKMVMADGSQDQMDQASTSLAKTMVPSSHRSPMQKRIHTTVELIHENWKRIW
VLTLWGIANFGLFMFKFIQYRNREVFEVMGYCVCIAKGAAETTKLNMALILLPVCRNT
LTSLRSTVLSTVVPFDDNINFHKVIALAIAIGASMHTIAHLTCDFPRLVSCPSDKFQQTLG
PFFNYVQPTWGTLVTSTPGWTGILLILIMSFSFTLATHSFRRSVVKLPSPLHHLAGFNSF
WYAHHLLVFAYILLVMHSYFLFLTREWYKKTGWMYIAVPVIFYASERATRRVREKNY
GVTVIKAAIYPGNVLSLYMKKPSNFKYKSGMYLFVKCPDVSPFEWHPFSITSAPGDDY
LSVHIRTLGDWTTELRNLFGKACEAEVSSKKATLSRLETTVIAEGPEENTRFPKIFVDGP
FGAPAQNYKKYDILFLIGLGIGATPFISILKDLLHNIKSNKEQQSMHDEEVGSAFKSNGP
SRAYFYWVTREQGSFEWFKGVMNEVAECDNDNAIEMHNYLTSVYEEGDARSALIAM
VQSLQHAKNGLDIVSGSKIRTHFARPNWRKVYSDLANTHKNARIGVFYCGSPTLTKTL
RELAIEFSHTTTTRFHFHKENF
>A0A287G593.1/161-261 Uncharacterized protein
{ECO:0000313|EnsemblPlants:HORVU1Hr1G071340.17}
AWTARHGHWCRAGAGASATLSGEAQTKGRREERQGRPALPRALHACRCSATAKIGA
TPLRNTRHSSPRFLLPSPLRLPTFQIYVRPPAASHRAVVPSPVRAMASSSEGYVDVPLGG
EQPPQQQQQQHSGPVMRKQPSRLASGMKRLASRVTSIRVPDSVMGLKRTHSSAQPAL
KGLRFLDKTAAGKDGWKSVEKRFDEMSADGRLHQENFAKCIGMADSKEFAGEVFVA
MARRRKIEPDQGITKEQLKEFWEEMSDNNFDARLRIFFDMCDKNGDGKLTEDEVKEV
SKQTTVLGSACQTLQISEFSFHAQIIVLSASANKLANLKKHAATYASLIMEELDPDGRG
HIEIWQLEKLLRKMVMADGSQDQMDQASTSLAKTMVPSSHRSPMQKRIHTTVELIHEN
WKRIWVLTLWGIANFGLFMFKFIQYRNREVFEVMGYCVCIAKGAAETTKLNMALILLP
VCRNTLTSLRSTVLSTVVPFDDNINFHKVHSLPLPFPFQDNGITSCIMGCTAQHGPPLTIT
MYPGYCARNCNWSEYAYDCSPDLRLPKAGLLPK
>A0A287G595.1/202-302 Uncharacterized protein
{ECO:0000313|EnsemblPlants:HORVU1Hr1G071340.18}
FRKNKKKLGAALLAPTSTHVAQSRLAVQRRAEQRSAGQGRLAWTARHGHWCRAGA
GASATLSGEAQTKGRREERQGRPALPRALHACRCSATAKIGATPLRNTRHSSPRFLLPS
PLRLPTFQIYVRPPAASHRAVVPSPVRAMASSSEGYVDVPLGGEQPPQQQQQQHSGPV
MRKQPSRLASGMKRLASRVTSIRVPDSVMGLKRTHSSAQPALKGLRFLDKTAAGKDG
WKSVEKRFDEMSADGRLHQENFAKCIGMADSKEFAGEVFVAMARRRKIEPDQGITKE
QLKEFWEEMSDNNFDARLRIFFDMCDKNGDGKLTEDEVKEVSKQTTVLGSACQTLQIS
EFSFHAQIIVLSASANKLANLKKHAATYASLIMEELDPDGRGHIEIWQLEKLLRKMVM
ADGSQDQMDQASTSLAKTMVPSSHRSPMQKRIHTTVELIHENWKRIWVLTLWGIANF
GLFMFKFIQYRNREVFEVMGYCVCIAKGAAETTKLNMALILLPVCRNTLTSLRSTVLST
VVPFDDNINFHKVIALAIAIGASMHTIAHLTCDFPRLVSCPSDKFQQTLGPFFNYVQPTW
GTLVTSTPGWTGILLILIMSFSFTLATHSFRRSVVKLPSPLHHLAGFNSFWYAHHLLVFA
YILLVMHSYFLFLTREWYKKTGWMYIAVPVIFYASERATRRVREKNYGVTVIKAAIYP
GNVLSLYMKKPSNFKYKSGMYLFVKCPDVSPFEWHPFSITSAPGDDYLSVHIRTLGDW

TTELRNLFGKACEAEVSSKKATLSRLETTVIAEGPEENTRFPKIFVDGPFGAPAQNYKK
YDILFLIGLGIGATPFISILKDLLHNIKSNKVTQVTESSFSYMNTDAECLK
>A0A287G599.1/6-67 Uncharacterized protein
{ECO:0000313|EnsemblPlants:HORVU1Hr1G071340.20}
TNYISESLSPEMYGSAFLLTGMADSKEFAGEVFVAMARRRKIEPDQGITKEQLKEFWEE
MSDNNFDARLRIFFDMCDKNGDGKLTEDEVKEIIVLSASANKLANLKKHAATYASLIM
EELDPDGRGHIEIWQLEKLLRKMVMADGSQDQMDQASTSLAKTMVPSSHRSPMQKRI
HTTVELIHENWKRIWVLTLWGIANFGLFMFKFIQYRNREVFEVMGYCVCIAKGAAETT
KLNMALILLPVCRNTLTSLRSTVLSTVVPFDDNINFHKVIALAIAIGASMHTIAHLTCDF
PRLVSCPSDKFQQTLGPFFNYVQPTWGTLVTSTPGWTGILLILIMSFSFTLATHSFRRSV
VKLPSPLHHLAGFNSFWYAHHLLVFAYILLVMHSYFLFLTREWYKKTGMDVHSGSCY
LLCQRESYQKSP
>A0A287G5A3.1/216-316 Uncharacterized protein
{ECO:0000313|EnsemblPlants:HORVU1Hr1G071340.26}
GSRCYDKPRLPLNAFRKNKKKLGAALLAPTSTHVAQSRLAVQRRAEQRSAGQGRLAW
TARHGHWCRAGAGASATLSGEAQTKGRREERQGRPALPRALHACRCSATAKIGATPL
RNTRHSSPRFLLPSPLRLPTFQIYVRPPAASHRAVVPSPVRAMASSSEGYVDVPLGGEQP
PQQQQQQHSGPVMRKQPSRLASGMKRLASRVTSIRVPDSVMGLKRTHSSAQPALKGL
RFLDKTAAGKDGWKSVEKRFDEMSADGRLHQENFAKCIGMADSKEFAGEVFVAMAR
RRKIEPDQGITKEQLKEFWEEMSDNNFDARLRIFFDMCDKNGDGKLTEDEVKEVSKQT
TVLGSACQTLQISEFSFHAQIIVLSASANKLANLKKHAATYASLIMEELDPDGRGHIEIW
QLEKLLRKMVMADGSQDQMDQASTSLAKTMVPSSHRSPMQKRIHTTVELIHENWKRI
WVLTLWGIANFGLFMFKFIQYRNREVFEVMGYCVCIAKGAAETTKLNMALILLPVCR
NTLTSLRSTVLSTVVPFDDNINFHKVIALAIAIGASMHTIAHLTCDFPRLVSCPSDKFQQT
LGPFFNYVQPTWGTLVTSTPGWTGILLILIMSFSFTLATHSFRRSVVKLPSPLHHLAGFN
SFWYAHHLLVFAYILLVMHSYFLFLTREWYKKTVLLLLSIMFGKTSYEFMNVHQLKL
>A0A287G5A5.1/162-262 Uncharacterized protein
{ECO:0000313|EnsemblPlants:HORVU1Hr1G071340.27}
LAWTARHGHWCRAGAGASATLSGEAQTKGRREERQGRPALPRALHACRCSATAKIG
ATPLRNTRHSSPRFLLPSPLRLPTFQIYVRPPAASHRAVVPSPVRAMASSSEGYVDVPLG
GEQPPQQQQQQHSGPVMRKQPSRLASGMKRLASRVTSIRVPDSVMGLKRTHSSAQPA
LKGLRFLDKTAAGKDGWKSVEKRFDEMSADGRLHQENFAKCIGMADSKEFAGEVFV
AMARRRKIEPDQGITKEQLKEFWEEMSDNNFDARLRIFFDMCDKNGDGKLTEDEVKEI
IVLSASANKLANLKKHAATYASLIMEELDPDGRGHIEIWQLEKLLRKMVMADGSQDQ
MDQASTSLAKTMVPSSHRSPMQKRIHTTVELIHENWKRIWVLTLWGIANFGLFMFKFI
QYRNREVFEVMGYCVCIAKGAAETTKLNMALILLPVCRNTLTSLRSTVLSTVVPFDDNI
NFHKVIALAIAIGASMHTIAHLTCDFPRLVSCPSDKFQQTLGPFFNYVQPTWGTLVTSTP
GWTGILLILIMSFSFTLATHSFRRSVVKLPSPLHHLAGFNSFWYAHHLLVFAYILLVMHS
YFLFLTREWYKKTVLLLLSIMFGKTSYEFMNVHQLKL
>A0A287G5B0.1/149-249 Uncharacterized protein
{ECO:0000313|EnsemblPlants:HORVU1Hr1G071340.31}
GAGASATLSGEAQTKGRREERQGRPALPRALHACRCSATAKIGATPLRNTRHSSPRFLL
PSPLRLPTFQIYVRPPAASHRAVVPSPVRAMASSSEGYVDVPLGGEQPPQQQQQQHSGP
VMRKQPSRLASGMKRLASRVTSIRVPDSVMGLKRTHSSAQPALKGLRFLDKTAAGKD
GWKSVEKRFDEMSADGRLHQENFAKCIGMADSKEFAGEVFVAMARRRKIEPDQGITK

EQLKEFWEEMSDNNFDARLRIFSTRCDKNGDGKLTEDEVKEIIVLSASANKLANLKKH
AATYASLIMEELDPDGRGHIEVPGNE
>A0A287G5B2.1/212-312 Uncharacterized protein
{ECO:0000313|EnsemblPlants:HORVU1Hr1G071340.28}
YDKPRLPLNAFRKNKKKLGAALLAPTSTHVAQSRLAVQRRAEQRSAGQGRLAWTAR
HGHWCRAGAGASATLSGEAQTKGRREERQGRPALPRALHACRCSATAKIGATPLRNT
RHSSPRFLLPSPLRLPTFQIYVRPPAASHRAVVPSPVRAMASSSEGYVDVPLGGEQPPQQ
QQQQHSGPVMRKQPSRLASGMKRLASRVTSIRVPDSVMGLKRTHSSAQPALKGLRFL
DKTAAGKDGWKSVEKRFDEMSADGRLHQENFAKCIGMADSKEFAGEVFVAMARRR
KIEPDQGITKEQLKEFWEEMSDNNFDARLRIFFDMCDKNGDGKLTEDEVKEVSKQTTV
LGSACQTLQISEFSFHAQIIVLSASANKLANLKKHAATYASLIMEELDPDGRGHIEIWQL
EKLLRKMVMADGSQDQMDQASTSLAKTMVPSSHRSPMQKRIHTTVELIHENWKRIW
VLTLWGIANFGLFMFKFIQYRNREVFEVMGYCVCIAKGAAETTKLNMALILLPVCRNT
LTSLRSTVLSTVVPFDDNINFHKVIALAIAIGASMHTIAHLTCDFPRLVSCPSDKFQQTLG
PFFNYVQPTWGTLVTSTPGWTGILLILIMSFSFTLATHSFRRSVVKLPSPLHHLAGFNSF
WYAHHLLVFAYILLVMHSYFLFLTREWYKKTGWMYIAVPVIFYASERATRRVREKNY
GVTVIKVSYRSYKNTTNKFLQYTLKSQGKW
>A0A287G5B5.1/162-262 Uncharacterized protein
{ECO:0000313|EnsemblPlants:HORVU1Hr1G071340.36}
LAWTARHGHWCRAGAGASATLSGEAQTKGRREERQGRPALPRALHACRCSATAKIG
ATPLRNTRHSSPRFLLPSPLRLPTFQIYVRPPAASHRAVVPSPVRAMASSSEGYVDVPLG
GEQPPQQQQQQHSGPVMRKQPSRLASGMKRLASRVTSIRVPDSVMGLKRTHSSAQPA
LKGLRFLDKTAAGKDGWKSVEKRFDEMSADGRLHQENFAKCTGMADSKEFAGEVFV
AMARRRKIEPDQGITKEQLKEFWEEMSDNNFDARLRIFFDMCDKNGDGKLTEDEVKEI
IVLSASANKLANLKKHAATYASLIMEELDPDGRGHIEVPGNE
>A0A287G5B6.1/212-312 Uncharacterized protein
{ECO:0000313|EnsemblPlants:HORVU1Hr1G071340.29}
YDKPRLPLNAFRKNKKKLGAALLAPTSTHVAQSRLAVQRRAEQRSAGQGRLAWTAR
HGHWCRAGAGASATLSGEAQTKGRREERQGRPALPRALHACRCSATAKIGATPLRNT
RHSSPRFLLPSPLRLPTFQIYVRPPAASHRAVVPSPVRAMASSSEGYVDVPLGGEQPPQQ
QQQQHSGPVMRKQPSRLASGMKRLASRVTSIRVPDSVMGLKRTHSSAQPALKGLRFL
DKTAAGKDGWKSVEKRFDEMSADGRLHQENFAKCIGMADSKEFAGEVFVAMARRR
KIEPDQGITKEQLKEFWEEMSDNNFDARLRIFFDMCDKNGDGKLTEDEVKEIIVLSASA
NKLANLKKHAATYASLIMEELDPDGRGHIEIWQLEKLLRKMVMADGSQDQMDQAST
SLAKTMVPSSHRSPMQKRIHTTVELIHENWKRIWVLTLWGIANFGLFMFKFIQYRNRE
VFEVMGYCVCIAKGAAETTKLNMALILLPVCRNTLTSLRSTVLSTVVPFDDNINFHKVI
ALAIAIGASMHTIAHLTCDFPRLVSCPSDKFQQTLGPFFNYVQPTWGTLVTSTPGWTGI
LLILIMSFSFTLATHSFRRSVVKLPSPLHHLAGFNSFWYAHHLLVFAYILLVMHSYFLFL
TREWYKKTGWMYIAVPVIFYASERATRRVREKNYGVTVIKAAIYPGNVLSLYMKKPS
NFKYKSGMYLFVKCPDVSPFEWHPFSITSAPGDDYLSVHIRTLGDWTTELRNLFGKAC
EAEVSSKKATLSRLETTVIAEGPEENTRFPKIFVDGPFGAPAQNYKKYDILFLIGLGIGAT
PFISILKDLLHNIKSNKEQQSMHDEEVGSAFKSNGPSRAYFYWVTREQGSFEWFKGVM
NEVAECDNDNAIEMHNYLTSVYEEGDARSALIAMVQSLQHAKNGLDIVSGSKIRTHFA
RPNWRKVYSDLANTHKNARIGVFYCGSPTLTKTLRELAIEFSHTTTTRFHFHKENF
>A0A287G5B7.1/4-62 Uncharacterized protein
{ECO:0000313|EnsemblPlants:HORVU1Hr1G071340.32}

IQYCSLFQNMPDRFSGMADSKEFAGEVFVAMARRRKIEPDQGITKEQLKEFWEEMSDN
NFDARLRIFFDMCDKNGDGKLTEDEVKEIIVLSASANKLANLKKHAATYASLIMEELD
PDGRGHIEIWQLEKLLRKMVMADGSQDQMDQASTSLAKTMVPSSHRSPMQKRIHTTV
ELIHENWKRIWVLTLWGIANFGLFMFKFIQYRNREVFEVMGYCVCIAKGAAETTKLN
MALILLPVCRNTLTSLRSTVLSTVVPFDDNINFHKVIALAIAIGASMHTIAHLTCDFPRLV
SCPSDKFQQTLGPFFNYVQPTWGTLVTSTPGWTGILLILIMSFSFTLATHSFRRSVVKLP
SPLHHLAGFNSFWYAHHLLVFAYILLVMHSYFLFLTREWYKKTGWMYIAVPVIFYASE
RATRRVREKNYGVTVIKAAIYPGNVLSLYMKKPSNFKYKSGMYLFVKCPDVSPFEWH
PFSITSAPGDDYLSVHIRTLGDWTTELRNLFGKACEAEVSSKKATLSRLETTVIAEGPEE
NTRFPKIFVDGPFGAPAQNYKKYDILFLIGLGIGATPFISILKDLLHNIKSNKEQQSMHDE
EVGSAFKSNGPSRAYFYWVTREQGSFEWFKGVMNEVAECDNDNAIEMHNYLTSVYE
EGDARSALIAMVQSLQHAKNGLDIVSGSKIRTHFARPNWRKVYSDLANTHKNARIGVF
YCGSPTLTKTLRELAIEFSHTTTTRFHFHKENF
>A0A287G5C2.1/161-261 Uncharacterized protein
{ECO:0000313|EnsemblPlants:HORVU1Hr1G071340.37}
AWTARHGHWCRAGAGASATLSGEAQTKGRREERQGRPALPRALHACRCSATAKIGA
TPLRNTRHSSPRFLLPSPLRLPTFQIYVRPPAASHRAVVPSPVRAMASSSEGYVDVPLGG
EQPPQQQQQQHSGPVMRKQPSRLASGMKRLASRVTSIRVPDSVMGLKRTHSSAQPAL
KGLRFLDKTAAGKDGWKSVEKRFDEMSADGRLHQENFAKCIGMADSKEFAGEVFVA
MARRRKIEPDQGITKEQLKEFWEEMSDNNFDARLRIFFDMCDKNGDGKLTEDEVKEII
VLSASANKLANLKKHAATYASLIMEELDPDGRGHIEVPGNE
>A0A287G670.1/128-225 Uncharacterized protein
{ECO:0000313|EnsemblPlants:HORVU1Hr1G072160.1}
MPNRVGADSGGGGGVGDPVEASGGVHERAPRPGKSARFADPVSAPRGGGGGNDDVE
ITLDVREDSVVVRSVKPVAAGGGEDSGVTPENRSSSSYGHGVLRIASTRDKQVSRELRP
VASFRRRGGGPSRIDRFKPVATHALEGLKFISGTDGAAGWTAAESFFDKKAKNGRLPR
SKFGGCIGMKEAAFAGELFDALARRRNIAGDSINKAELREFWDQISDTSFDSRLQTFLD
MVDKDADGKISEQEVKQIITLSVSANKLTMAPHQCEEYARLIMEALDPHGLRYIELYN
LKMLLLEAPGESTTNNRKLNKLLSERLRPTVDPSLVWRLYRHAKCFLEDNWRRCWV
MLLWLSICVGLFAWKFVQYRHHDVFGVMGYCVCVAKGGAETLKFNMALTLLPVCRN
TITWLRSHTGAGRYVPFNDNLSFHKAIAVGITVGVGLHAISHLACDFPRLLHVMDDEY
GPMKPFFGDNKPPNYWWFVRGTEGWTGLVMLVLMVVAFTFATGPLRKGKLQLPKVK
RLESLDSHPKPIELDRLAMLINTSRSRLTWLVNTSLKCFTGYNAFWYTHHLFLIVYAFLI
VHGHFLYLTKKWQKKSVRMNFHYYLTTSRTEQC
>A0A287G673.1/7-104 Uncharacterized protein
{ECO:0000313|EnsemblPlants:HORVU1Hr1G072160.6}
RGGGPSRIDRFKPVATHALEGLKFISGTDGAAGWTAAESFFDKKAKNGRLPRSKFGGCI
GMKEAAFAGELFDALARRRNIAGDSINKAELREFWDQISDTSFDSRLQTFLDMVDKDA
DGKISEQEVKQIITLSVSANKLTMAPHQCEEYARLIMEALDPHGLRYIELYNLKMLLLE
APGESTTNNRKLNKLLSERLRPTVDPSLVWRLYRHAKCFLEDNWRRCWVMLLWLSIC
VGLFAWKFVQYRHHDVFGVMGYCVCVAKGGAETLKFNMALTLLPVCRNTITWLRSH
TGAGRYVPFNDNLSFHKAIAVGITVGVGLHAISHLACDFPRLLHVMDDEYGPMKPFFG
DNKPPNYWWFVRGTEGWTGLVMLVLMVVAFTFATGPLRKGKLQLPKSLKCFTGYNA
FWYTHHLFLIVYAFLIVHGHFLYLTKKWQKKSTWMYLAVPMVVYASERLTRALRSSV
RSVKKMKVAVHPHPATLLSLHLSKPQGFRYKSGQYIFVKCPDVSPSQWHPFSITSAPED
DHVSVHIKAAGDWTNQLRNAFLKVCSTPTEGKTEILRAEYSRDDVNSNPSFPKVLIDGP

YGAPAQDYKEYDIVLLVGLGIGATPMISIIKDIINNAKRLGGVDVESGNGNGNGNGNAS
TFRTRRAYFYWVTREQGSLEWFRGVMDEVAEADEKRIIELHNHCTSVYGKGDARSALI
AMLQSLYYAKNGVDVVSGSSRVMTHFGRPDWDQVYRRIADENEGKRVGQCHPKAN
YTCSFVSFHFNQWTNAAAHKHAHTP
>A0A287G674.1/53-150 Uncharacterized protein
{ECO:0000313|EnsemblPlants:HORVU1Hr1G072140.3}
HRVRPVARGEESDVKLLASSSGHGALKNARTRIEQVRQELRRAASISRRGGGFDRSMP
SAPAHALEGLRFISGTDASEGWAKAEWFFKNNAENGRLPRSKFGECIGMKEAAFAGEL
FDALGRRRGNSADSIDKAELLEYWDQISDTDYTTRLQLFFDRVDKDSDGRISKVEFKQI
ITLSASANKLKVAEQDSEKYARQIMEKLDPYGLGYIELYDLETLFVKPSNDPASIETKT
NNHEPSKPLTKNNPFMRWYRHTRYFVKDNWRRCWVMLLWLSICTSLFAWKFVQYRH
RAVFQVMGYCVCVAKGGAETLKFNMALTLLPVCRNTVTWLRTRTAAGQFVPFNDNL
NFHKVIAVGISVGASL
>A0A287G675.1/128-225 Uncharacterized protein
{ECO:0000313|EnsemblPlants:HORVU1Hr1G072160.3}
MPNRVGADSGGGGGVGDPVEASGGVHERAPRPGKSARFADPVSAPRGGGGGNDDVE
ITLDVREDSVVVRSVKPVAAGGGEDSGVTPENRSSSSYGHGVLRIASTRDKQVSRELRP
VASFRRRGGGPSRIDRFKPVATHALEGLKFISGTDGAAGWTAAESFFDKKAKNGRLPR
SKFGGCIGMKEAAFAGELFDALARRRNIAGDSINKAELREFWDQISDTSFDSRLQTFLD
MVDKDADGKISEQEVKQVNSQSVSANKLTMAPHQCEEYARLIMEALDPHGLRYIELY
NLKMLLLEAPGESTTNNRKLNKLLSERLRPTVDPSLVWRLYRHAKCFLEDNWRRCWV
MLLWLSICVGLFAWKFVQYRHHDVFGVMGYCVCVAKGGAETLKFNMALTLLPVCRN
TITWLRSHTGAGRYVPFNDNLSFHKAIAVGITVGVGLHAISHLACDFPRLLHVMDDEY
GPMKPFFGDNKPPNYWWFVRGTEGWTGLVMLVLMVVAFTFATGPLRKGKLQLPKVK
RLESLDSHPKPIELDRLAMLINTSRSRLTWLVNTSLKCFTGYNAFWYTHHLFLIVYAFLI
VHGHFLYLTKKWQKKSVRMNFHYYLTT
>A0A287G679.1/128-225 Uncharacterized protein
{ECO:0000313|EnsemblPlants:HORVU1Hr1G072160.2}
MPNRVGADSGGGGGVGDPVEASGGVHERAPRPGKSARFADPVSAPRGGGGGNDDVE
ITLDVREDSVVVRSVKPVAAGGGEDSGVTPENRSSSSYGHGVLRIASTRDKQVSRELRP
VASFRRRGGGPSRIDRFKPVATHALEGLKFISGTDGAAGWTAAESFFDKKAKNGRLPR
SKFGGCIGMKEAAFAGELFDALARRRNIAGDSINKAELREFWDQISDTSFDSRLQTFLD
MVDKDADGKISEQEVKQIITLSVSANKLTMAPHQCEEYARLIMEALDPHGLRYIELYN
LKMLLLEAPGESTTNNRKLNKLLSERLRPTVDPSLVWRLYRHAKCFLEDNWRRCWV
MLLWLSICVGLFAWKFVQYRHHDVFGVMGYCVCVAKGGAETLKFNMALTLLPVCRN
TITWLRSHTGAGRYVPFNDNLSFHKAIAVGITVGVGLHAISHLACDFPRLLHVMDDEY
GPMKPFFGDNKPPNYWWFVRGTEGWTGLVMLVLMVVAFTFATGPLRKGKLQLPKVK
RLESLDSHPKPIELDRLAMLINTSRSRLTWLVNTSLKCFTGYNAFWYTHHLFLIVYAFLI
VHGHFLYLTKKWQKKSTWMYLAVPMVVYASERLTRALRSSVRSVKKMKVAVHPHP
ATLLSLHLSKPQGFRYKSGQYIFVKCPDVSPSQWHPFSITSAPEDDHVSVHIKAAGDWT
NQLRNAFLKVCSTPTEGKTEILRAEYSRDDVNSNPSFPKVLIDGPYGAPAQDYKEYDIV
LLVGLGIGATPMISIIKDIINNAKRLGGVDVESGNGNGNGNGNASTFRTRRAYFYWVTR
EQGSLEWFRGVMDEVAEADEKRIIELHNHCTSVYGKGDARSALIAMLQSLYYAKNGV
DVVSGSSRVMTHFGRPDWDQVYRRIADENEGKRVGSLLLR
>A0A287G680.1/2-99 Uncharacterized protein
{ECO:0000313|EnsemblPlants:HORVU1Hr1G072160.8}

SRIDRFKPVATHALEGLKFISGTDGAAGWTAAESFFDKKAKNGRLPRSKFGGCIGMKE
AAFAGELFDALARRRNIAGDSINKAELREFWDQISDTSFDSRLQTFLDMVDKDADGKIS
EQEVKQIITLSVSANKLTMAPHQCEEYARLIMEALDPHGLRYIELYNLKMLLLEAPGES
TTNNRKLNKLLSERLRPTVDPSLVWRLYRHAKCFLEDNWRRCWVMLLWLSICVGLFA
WKFVQYRHHDVFGVMGYCVCVAKGGAETLKFNMALTLLPVCRNTITWLRSHTGAGR
YVPFNDNLSFHKAIAVGITVGVGLHAISHLACDFPRLLHVMDDEYGPMKPFFGDNKPP
NYWWFVRGTEGWTGLVMLVLMVVAFTFATGPLRKGKLQLPKSLKCFTGYNAFWYT
HHLFLIVYAFLIVHGHFLYLTKKWQKKSTWMYLAVPMVVYASERLTRALRSSVRSVK
KMKVAVHPHPATLLSLHLSKPQGFRYKSGQYIFVKCPDVSPSQWHPFSITSAPEDDHVS
VHIKAAGDWTNQLRNAFLKVCSTPTEGKTEILRAEYSRDDVNSNPRASRRC
>A0A287G681.1/122-219 Uncharacterized protein
{ECO:0000313|EnsemblPlants:HORVU1Hr1G072140.1}
MPSRVDADDARGGPGVGEIVEARASGGVRVPPRKTARFAEPVSPQRVGDDDDDVVEE
ITLDMRDGTKAVHRVRPVARGEESDVKLLASSSGHGALKNARTRIEQVRQELRRAASI
SRRGGGFDRSMPSAPAHALEGLRFISGTDASEGWAKAEWFFKNNAENGRLPRSKFGEC
IGMKEAAFAGELFDALGRRRGNSADSIDKAELLEYWDQISDTDYTTRLQLFFDMVDKD
SDGRISKVEFKQIITLSASANKLKVAEQDSEKYARQIMEKLDPYGLGYIELYDLETLFVK
PSNDPASIETKTNNHEPSKPLTKNNPFMRWYRHTRYFVKDNWRRCWVMLLWLSICTS
LFAWKFVQYRHRAVFQVMGYCVCVAKGGAETLKFNMALTLLPVCRNTVTWLRTRT
AAGQFVPFNDNLNFHKVIAVGISVGASLHVISHLACDFPRLLHATDDEYEPMKPFFGDV
KPPNYWWFVKGTEGWTGLVMLVLMAIAFTLATGWFRNRALRLSKPKKNDNRPQSKK
PDSPPRPLTRFMRASRKRLNMLVNAFLDRFTGYNSFLYTHHFFIIVYALLIVHGHFLYLT
KKWQKKTTWMYLAVPMIVYACERLTRTLRSRMRSVQKVKVAVHPDSAALLSLRLSK
PEGFTYKSGQYIFVKCPDVSRFEWHPFSITSAPEDDHISVHIKAMGDWTKKLRKTFFEA
SEALTEDKTEIRRLEYEHGDAMPAPRDGLKYPTVLIDGPYGAPAQDYKQYDTLLLVGL
GIGATPMISIIKDIINNMKRLPGDIESGNPGDAGTSSSSFRTRRAYFYWVTREQESLEWF
HGIMDEVAETDKNGVIELHVHCTSVHEEGDARSAPITIIQSLNYDKHGIDIISGTRVKTS
LGRANWGQVYKHIAQENQGKRVGVFYCGMPMLTKELREHAKVYSRETSTTFEFHKE
NF
>A0A287G682.1/1-98 Uncharacterized protein
{ECO:0000313|EnsemblPlants:HORVU1Hr1G072160.9}
RIDRFKPVATHALEGLKFISGTDGAAGWTAAESFFDKKAKNGRLPRSKFGGCIGMKEA
AFAGELFDALARRRNIAGDSINKAELREFWDQISDTSFDSRLQTFLDMVDKDADGKISE
QEVKQIITLSVSANKLTMAPHQCEEYARLIMEALDPHGLRYIELYNLKMLLLEAPGEST
TNNRKLNKLLSERLRPTVDPSLVWRLYRHAKCFLEDNWRRCWVMLLWLSICVGLFA
WKFVQYRHHDVFGVMGYCVCVAKGGAETLKFNMALTLLPVCRNTITWLRSHTGAGR
YVPFNDNLSFHKAIAVGITVGVGLHAISHLACDFPRLLHVMDDEYGPMKPFFGDNKPP
NYWWFVRGTEGWTGLVMLVLMVVAFTFATGPLRKGKLQLPKSLKCFTGYNAFWYT
HHLFLIVYAFLIVHGHFLYLTKKWQKKSTWMYLAVPMVVYASERLTRALRSSVRSVK
KMKVAVHPHPATLLSLHLSKPQGFRYKSGQYIFVKCPDVSPSQWHPFSITSAPEDDHVS
VHIKAAGDWTNQLRNAFLKVCSTPTEGKTEILRAEYSRDDVNSNPSFPKVLIDGPYGAP
AQDYKEYDIVLLVGLGIGATPMISIIKDIINNAKRLGGVDVESGNGNGNGNGNASTFRT
RRAYFYWVTREQGSLEWFRGVMDEVAEADEKRIIELHNHCTSVYGKGDARSALIAML
QSLYYAKNGVDVVSGSSRVMTHFGRPDWDQVYRRIADENEGKRVGQCHPKANYTCS
FVSFHFNQWTNAAAHKHRNF

>A0A287G684.1/7-104 Uncharacterized protein
{ECO:0000313|EnsemblPlants:HORVU1Hr1G072160.7}
RGGGPSRIDRFKPVATHALEGLKFISGTDGAAGWTAAESFFDKKAKNGRLPRSKFGGCI
GMKEAAFAGELFDALARRRNIAGDSINKAELREFWDQISDTSFDSRLQTFLDMVDKDA
DGKISEQEVKQIITLSVSANKLTMAPHQCEEYARLIMEALDPHGLRYIELYNLKMLLLE
APGESTTNNRKLNKLLSERLRPTVDPSLVWRLYRHAKCFLEDNWRRCWVMLLWLSIC
VGLFAWKFVQYRHHDVFGVMGYCVCVAKGGAETLKFNMALTLLPVCRNTITWLRSH
TGAGRYVPFNDNLSFHKAIAVGITVGVGLHAISHLACDFPRLLHVMDDEYGPMKPFFG
DNKPPNYWWFVRGTEGWTGLVMLVLMVVAFTFATGPLRKGKLQLPKTSLKCFTGYN
AFWYTHHLFLIVYAFLIVHGHFLYLTKKWQKKSTWMYLAVPMVVYASERLTRALRSS
VRSVKKMKVAVHPHPATLLSLHLSKPQGFRYKSGQYIFVKCPDVSPSQWHPFSITSAPE
DDHVSVHIKAAGDWTNQLRNAFLKVCSTPTEGKTEILRAEYSRDDVNSNPSFPKVLID
GPYGAPAQDYKEYDIVLLVGLGIGATPMISIIKDIINNAKRLGGVDVESGNGNGNGNGN
ASTFRTRRAYFYWVTREQGSLEWFRGVMDEVAEADEKRIIELHNHCTSVYGKGDARS
ALIAMLQSLYYAKNGVDVVSGSSRVMTHFGRPDWDQVYRRIADENEGKRVGQCHPK
ANYTCSFVSFHFNQWTNAAAHKHRNF
>A0A287GHC7.1/12-112 Uncharacterized protein
{ECO:0000313|EnsemblPlants:HORVU1Hr1G081950.11}
LAARAERRQRAQLDRTKSTAQRAIKGLRFISGNTKASNNAWIEVQRNFDRLALDGRLS
RADFPQCIGMTESKEFAMELFDTLSRRRQMQVDHINKDELREIWLQITDNSFDSRLQIFF
DMVDKDADGHITEAEVKEIIMLSASANKLARLKEQAEEYAALIMEELDPEGLGYIELW
QLETLLLQKDTYVNYSQALSYTSQALSQNLAGLRYRSPIRKMSSKLSYYLEDNWKRL
WVLALWIGIMAGLFIWKFIQYRNRYVFHVMGYCVTIAKGAAETLKLNMALILLPVCR
NTITWLRNTRAARALPFDDNINFHKTIAAAIVVGVILHAGNHLACDFPRLIDSSDQMYA
PLGKYFGETKPTYLALVKGVEGVTGVIMVVCMLIAFTLATRWFRRSLVKLPKPFDKLT
GFNAFWYSHHLFIIVYISLVIHGERLYLILDWYKRTVSLYLFFCFTLFS
>A0A287GHC9.1/175-275 Uncharacterized protein
{ECO:0000313|EnsemblPlants:HORVU1Hr1G081950.10}
EQGAIMRGAGGGGTPGRPRWGGSGATTPRSLSTGSSPRGSDRSSDDGEELVEVTLDLQ
EDDTIVLRSVEPAAATATATAASVPVSSGASPSVMGWSAEPTPPGPSSRSRSPAIRRSSS
HRLLQFSQELKAGVSRAKQISQDLTKRFTRTQSRAALPLSGIESALAARAERRQRAQLD
RTKSTAQRAIKGLRFISGNTKASNNAWIEVQRNFDRLALDGRLSRADFPQCIGMTESKE
FAMELFDTLSRRRQMQVDHINKDELREIWLQITDNSFDSRLQIFFDMVDKDADGHITEA
EVKEIIMLSASANKLARLKEQAEEYAALIMEELDPEGLGYIELWQLETLLLQKDTYVN
YSQALSYTSQALSQNLAGLRYRSPIRKMSSKLSYYLEDNWKRLWVLALWIGIMAGLFI
WKFIQYRNRYVFHVMGYCVTIAKGAAETLKLNMALILLPVCRNTITWLRNTRAARAL
PFDDNINFHKTIAAAIVVGVILHAGNHLACDFPRLIDSSDQMYAPLGKYFGETKPTYLA
LVKGVEGVTGVIMVVCMLIAFTLATRWFRRSLVKLPKPFDKLTGFNAFWYSHHLFIIV
YISLVIHGERLYLILDWYKRTTWMYLAVPVGLYVGERTLRFFRSGSYSVRILKVAIYPG
NVLTLQMSKPPTFRYKSGQYMFVQCPAVSPFEWHPFSITSAPGDDFLSIHVRQLGDWT
RELKRVFSAACEPPMNGKSGLLRADENTKKTFPKLLIDGPYGSPAQDYSKYDVLLLVG
LGIGATPFISILKDLINNIIKMEEEDEASTDLYPPIGPSKASVDLDTLMRITSKPKRVFKTT
NAYFYWVTREQGSFDWFKGIMNEIAELDQRNIIEMHNYLTSVYEEGDARSALITMLQA
LNHAKNGVDVVSGTRVRTHFARPNFKRVLSKVAAKHPYAKIGVFYCGAPVLAQELSN
LCHEFNGKCTTKFEFHKEHF

>A0A287GHD2.1/174-274 Uncharacterized protein
{ECO:0000313|EnsemblPlants:HORVU1Hr1G081950.12}
AGGGGTPGRPRWGGSGATTPRSLSTGSSPRGSDRSSDDGEELVEVTLDLQEDDTIVLRS
VEPAAATATATAASVPVSSGASPSVMGWSAEPTPPGPSSRSRSPAIRRSSSHRLLQFSQE
LKAGVSRAKQISQDLTKRFTRTQSRAXXXPPPPAQPLSGIESALAARAERRQRAQLDRT
KSTAQRAIKGLRFISGNTKASNNAWIEVQRNFDRLALDGRLSRADFPQCIGMTESKEFA
MELFDTLSRRRQMQVDHINKDELREIWLQITDNSFDSRLQIFFDMVDKDADGHITEAE
VKEIIMLSASANKLARLKEQAEEYAALIMEELDPEGLGYIELWQLETLLLQKDTYVNYS
QALSYTSQALSQNLAGLRYRSPIRKMSSKLSYYLEDNWKRLWVLALWIGIMAGLFIW
KFIQYRNRYVFHVMGYCVTIAKGAAETLKLNMALILLPVCRNTITWLRNTRAARALPF
DDNINFHKTIAAAIVVGVILHAGNHLACDFPRLIDSSDQMYAPLGKYFGETKPTYLALV
KGVEGVTGVIMVVCMLIAFTLATRWFRRSLVKLPKPFDKLTGFNAFWYSHHLFIIVYIS
LVIHGERLYLILDWYKRTTWMYLAVPVGLYVGERTLRFFRSGSYSVRILKVAIYPGNV
LTLQMSKPPTFRYKSGQYMFVQCPAVSPFEWHPFSITSAPGDDFLSIHVRQLGDWTREL
KRVFSAACEPPMNGKSGLLRADENTKKTFPKLLIDGPYGSPAQDYSKYDVLLLVGLGI
GATPFISILKDLINNIIKMEEEDEASTDLYPPIGPSKASVDLDTLMRITSKPKRVFKTTNA
YFYWVTREQGSFDWFKGIMNEIAELDQRNIIEMHNYLTSVYEEGDARSALITMLQALN
HAKNGVDVVSGTRGEMWKRIMLACLL
>A0A287GHD4.1/9-109 Uncharacterized protein
{ECO:0000313|EnsemblPlants:HORVU1Hr1G081950.3}
RAERRQRAQLDRTKSTAQRAIKGLRFISGNTKASNNAWIEVQRNFDRLALDGRLSRAD
FPQCIGMTESKEFAMELFDTLSRRRQMQVDHINKDELREIWLQITDNSFDSRLQIFFDM
VDKDADGHITEAEVKEIIMLSASANKLARLKEQAEEYAALIMEELDPEGLGYIELWQLE
TLLLQKDTYVNYSQALSYTSQALSQNLAGLRYRSPIRKMSSKLSYYLEDNWKRLWVL
ALWIGIMAGLFIWKFIQYRNRYVFHVMGYCVTIAKGAAETLKLNMALILLPVCRNTIT
WLRNTRAARALPFDDNINFHKTIAAAIVVGVILHAGNHLACDFPRLIDSSDQMYAPLG
KYFGETKPTYLALVKGVEGVTGVIMVVCMLIAFTLATRWFRRSLVKLPKPFDKLTGFN
AFWYSHHLFIIVYISLVIHGERLYLILDWYKRTVSLYLFFCFTLFS
>A0A287GHD6.1/5-105 Uncharacterized protein
{ECO:0000313|EnsemblPlants:HORVU1Hr1G081950.2}
RQRAQLDRTKSTAQRAIKGLRFISGNTKASNNAWIEVQRNFDRLALDGRLSRADFPQCI
GMTESKEFAMELFDTLSRRRQMQVDHINKDELREIWLQITDNSFDSRLQIFFDMVDKD
ADGHITEAEVKEIIMLSASANKLARLKEQAEEYAALIMEELDPEGLGYIELWQLETLLL
QKDTYVNYSQALSYTSQALSQNLAGLRYRSPIRKMSSKLSYYLEDNWKRLWVLALWI
GIMAGLFIWKFIQYRNRYVFHVMGYCVTIAKGAAETLKLNMALILLPVCRNTITWLRN
TRAARALPFDDNINFHKTIAAAIVVGVILHAGNHLACDFPRLIDSSDQMYAPLGKYFGE
TKPTYLALVKGVEGVTGVIMVVCMLIAFTLATRWFRRSLVKLPKPFDKLTGFNAFWYS
HHLFIIVYISLVIHGERLYLILDWYKRTVSLYLFFCFTLFS
>A0A287GHD7.1/186-286 Uncharacterized protein
{ECO:0000313|EnsemblPlants:HORVU1Hr1G081950.9}
TRSHHAGGRRRRDPGPAAVGRLGRHHAALAQHRLLAARLRPQLRRRGGARRGHARP
AGGRHHCAAQRRAGRRHRHRHGRLRPRLVGRVAVGHGVERRAHAAGPVVPVALAG
DPPELLAPAAAVLAGAQGRGVPRQANLAGPHQALHAHPEPRRPRRAPATAATAAQPL
SGIESALAARAERRQRAQLDRTKSTAQRAIKGLRFISGNTKASNNAWIEVQRNFDRLAL
DGRLSRADFPQCIGMTESKEFAMELFDTLSRRRQMQVDHINKDELREIWLQITDNSFDS
RLQIFFDMVDKDADGHITEAEVKEIIMLSASANKLARLKEQAEEYAALIMEELDPEGLG

YIELWQLETLLLQKDTYVNYSQALSYTSQALSQNLAGLRYRSPIRKMSSKLSYYLEDN
WKRLWVLALWIGIMAGLFIWKFIQYRNRYVFHVMGYCVTIAKGAAETLKLNMALILL
PVCRNTITWLRNTRAARALPFDDNINFHKTIAAAIVVGVILHAGNHLACDFPRLIDSSD
QMYAPLGKYFGETKPTYLALVKGVEGVTGVIMVVCMLIAFTLATRWFRRSLVKLPKP
FDKLTGFNAFWYSHHLFIIVYISLVIHGERLYLILDWYKRTTWMYLAVPVGLYVGERT
LRFFRSGSYSVRILKVAIYPGNVLTLQMSKPPTFRYKSGQYMFVQCPAVSPFEWHPFSIT
SAPGDDFLSIHVRQLGDWTRELKRVFSAACEPPMNGKSGLLRADENTKKTFPKLLIDG
PYGSPAQDYSKYDVLLLVGLGIGATPFISILKDLINNIIKMEEEDEASTDLYPPIGPSKAS
VDLDTLMRITSKPKRVFKTTNAYFYWVTREQGSFDWFKGIMNEIAELDQRNIIEMHNY
LTSVYEEGDARSALITMLQALNHAKNGVDVVSGTRVRTHFARPNFKRVLSKVAAKHP
YAKIGVFYCGAPVLAQELSNLCHEFNGKCTTKFEFHKEHF
>A0A287GHD8.1/172-272 Uncharacterized protein
{ECO:0000313|EnsemblPlants:HORVU1Hr1G081950.5}
AGGGGTPGRPRWGGSGATTPRSLSTGSSPRGSDRSSDDGEELVEVTLDLQEDDTIVLRS
VEPAAATATATAASVPVSSGASPSVMGWSAEPTPPGPSSRSRSPAIRRSSSHRLLQFSQE
LKAGVSRAKQISQDLTKRFTRTQSRAALAEPPPPLSGIESALAARAERRQRAQLDRTKS
TAQRAIKGLRFISGNTKASNNAWIEVQRNFDRLALDGRLSRADFPQCIGMTESKEFAM
ELFDTLSRRRQMQVDHINKDELREIWLQITDNSFDSRLQIFFDMVDKDADGHITEAEVK
EIIMLSASANKLARLKEQAEEYAALIMEELDPEGLGYIELWQLETLLLQKDTYVNYSQA
LSYTSQALSQNLAGLRYRSPIRKMSSKLSYYLEDNWKRLWVLALWIGIMAGLFIWKFI
QYRNRYVFHVMGYCVTIAKGAAETLKLNMALILLPVCRNTITWLRNTRAARALPFDD
NINFHKTIAAAIVVGVILHAGNHLACDFPRLIDSSDQMYAPLGKYFGETKPTYLALVKG
VEGVTGVIMVVCMLIAFTLATRWFRRSLVKLPKPFDKLTGFNAFWYSHHLFIIVYISLVI
HGERLYLILDWYKRTTWMYLAVPVGLYVGERTLRFFRSGSYSVRILKVAIYPGNVLTL
QMSKPPTFRYKSGQYMFVQCPAVSPFEWHPFSITSAPGDDFLSIHVRQLGDWTRELKR
VFSAACEPPMNGKSGLLRADENTKKTFPKLLIDGPYGSPAQDYSKYDVLLLVGLGIGA
TPFISILKDLINNIIKMEEEDEASTDLYPPIGPSKASVDLDTLMRITSKPKRVFKTTNAYFY
WVTREQGSFDWFKGIMNEIAELDQRNIIEMHNYLTSVYEEGDARSALITMLQALNHAK
NGVDVVSGTRVRTHFARPNFKRVLSKVAAKHPYAKIGVFYCGAPVLAQELSNLCHEF
NGKCTTKFEFHKEHF
>A0A287GHD9.1/190-290 Uncharacterized protein
{ECO:0000313|EnsemblPlants:HORVU1Hr1G081950.8}
PSCGGPAAAGPRAGRGGAARAPPRRARSAPAPRRAAPTAAPTTGRSSSRSRSTCRRTTP
LCCAASSRPPPPPPPRPPPSPSRRARRRRSWGGAPSPRRRARRPGRARRRSAGAPRTGCC
SSRRSSRPGCPAPSKSRRTSPSASRAPRAAPPSXXXXXXXXXXXXPPPPAQPLSGIESAL
AARAERRQRAQLDRTKSTAQRAIKGLRFISGNTKASNNAWIEVQRNFDRLALDGRLSR
ADFPQCIGMTESKEFAMELFDTLSRRRQMQVDHINKDELREIWLQITDNSFDSRLQIFF
DMVDKDADGHITEAEVKEIIMLSASANKLARLKEQAEEYAALIMEELDPEGLGYIELW
QLETLLLQKDTYVNYSQALSYTSQALSQNLAGLRYRSPIRKMSSKLSYYLEDNWKRL
WVLALWIGIMAGLFIWKFIQYRNRYVFHVMGYCVTIAKGAAETLKLNMALILLPVCR
NTITWLRNTRAARALPFDDNINFHKTIAAAIVVGVILHAGNHLACDFPRLIDSSDQMYA
PLGKYFGETKPTYLALVKGVEGVTGVIMVVCMLIAFTLATRWFRRSLVKLPKPFDKLT
GFNAFWYSHHLFIIVYISLVIHGERLYLILDWYKRTTWMYLAVPVGLYVGERTLRFFRS
GSYSVRILKVAIYPGNVLTLQMSKPPTFRYKSGQYMFVQCPAVSPFEWHPFSITSAPGD
DFLSIHVRQLGDWTRELKRVFSAACEPPMNGKSGLLRADENTKKTFPKLLIDGPYGSP
AQDYSKYDVLLLVGLGIGATPFISILKDLINNIIKMEEEDEASTDLYPPIGPSKASVDLDT

LMRITSKPKRVFKTTNAYFYWVTREQGSFDWFKGIMNEIAELDQRNIIEMHNYLTSVY
EEGDARSALITMLQALNHAKNGVDVVSGTRVRTHFARPNFKRVLSKVAAKHPYAKIG
VFYCGAPVLAQELSNLCHEFNGKCTTKFEFHKEHF
>A0A287GHE1.1/181-281 Uncharacterized protein
{ECO:0000313|EnsemblPlants:HORVU1Hr1G081950.7}
MRGAGGGGTPGRPRWGGSGATTPRSLSTGSSPRGSDRSSDDGEELVEVTLDLQEDDTI
VLRSVEPAAATATATAASVPVSSGASPSVMGWSAEPTPPGPSSRSRSPAIRRSSSHRLLQ
FSQELKAGVSRAKQISQDLTKRFTRTQSRAALAEPPPPPPPAQPLSGIESALAARAERRQ
RAQLDRTKSTAQRAIKGLRFISGNTKASNNAWIEVQRNFDRLALDGRLSRADFPQCIG
MTESKEFAMELFDTLSRRRQMQVDHINKDELREIWLQITDNSFDSRLQIFFDMVDKDA
DGHITEAEVKEIIMLSASANKLARLKEQAEEYAALIMEELDPEGLGYIELWQLETLLLQ
KDTYVNYSQALSYTSQALSQNLAGLRYRSPIRKMSSKLSYYLEDNWKRLWVLALWIG
IMAGLFIWKFIQYRNRYVFHVMGYCVTIAKGAAETLKLNMALILLPVCRNTITWLRNT
RAARALPFDDNINFHKTIAAAIVVGVILHAGNHLACDFPRLIDSSDQMYAPLGKYFGET
KPTYLALVKGVEGVTGVIMVVCMLIAFTLATRWFRRSLVKLPKPFDKLTGFNAFWYS
HHLFIIVYISLVIHGERLYLILDWYKRTTWMYLAVPVGLYVGERTLRFFRSGSYSVRILK
VAIYPGNVLTLQMSKPPTFRYKSGQYMFVQCPAVSPFEWHPFSITSAPGDDFLSIHVRQ
LGDWTRELKRVFSAACEPPMNGKSGLLRADENTKKTFPKLLIDGPYGSPAQDYSKYD
VLLLVGLGIGATPFISILKDLINNIIKMEEEDEASTDLYPPIGPSKASVDLDTLMRITSKPK
RVFKTTNAYFYWVTREQGSFDWFKGIMNEIAELDQRNIIEMHNYLTSVYEEGDARSAL
ITMLQALNHAKNGVDVVSGTRVRTHFARPNFKRVLSKVAAKHPYAKIGVFYCGAPVL
AQELSNLCHEFNGKCTTKFEFHKEHF
>A0A287GHE5.1/158-258 Uncharacterized protein
{ECO:0000313|EnsemblPlants:HORVU1Hr1G081950.6}
RRRRDPGPAAVGRLGRHHAALAQHRLLAARLRPQLRRRGGARRGHARPAGGRHHCA
AQRRAGRRHRHRHGRLRPRRRARRPGRARRRSAGAPRTGCCSSRRSSRPGCPAPSKSR
RTSPSASRAPRAAPPSPSPRHPAQPLSGIESALAARAERRQRAQLDRTKSTAQRAIKGLR
FISGNTKASNNAWIEVQRNFDRLALDGRLSRADFPQCIGMTESKEFAMELFDTLSRRRQ
MQVDHINKDELREIWLQITDNSFDSRLQIFFDMVDKDADGHITEAEVKEIIMLSASANK
LARLKEQAEEYAALIMEELDPEGLGYIELWQLETLLLQKDTYVNYSQALSYTSQALSQ
NLAGLRYRSPIRKMSSKLSYYLEDNWKRLWVLALWIGIMAGLFIWKFIQYRNRYVFH
VMGYCVTIAKGAAETLKLNMALILLPVCRNTITWLRNTRAARALPFDDNINFHKTIAA
AIVVGVILHAGNHLACDFPRLIDSSDQMYAPLGKYFGETKPTYLALVKGVEGVTGVIM
VVCMLIAFTLATRWFRRSLVKLPKPFDKLTGFNAFWYSHHLFIIVYISLVIHGERLYLIL
DWYKRTTWMYLAVPVGLYVGERTLRFFRSGSYSVRILKVAIYPGNVLTLQMSKPPTFR
YKSGQYMFVQCPAVSPFEWHPFSITSAPGDDFLSIHVRQLGDWTRELKRVFSAACEPP
MNGKSGLLRADENTKKTFPKLLIDGPYGSPAQDYSKYDVLLLVGLGIGATPFISILKDLI
NNIIKMEEEDEASTDLYPPIGPSKASVDLDTLMRITSKPKRVFKTTNAYFYWVTREQGS
FDWFKGIMNEIAELDQRNIIEMHNYLTSVYEEGDARSALITMLQALNHAKNGVDVVSG
TRVRTHFARPNFKRVLSKVAAKHPYAKIGVFYCGAPVLAQELSNLCHEFNGKCTTKFE
FHKEHF
>A0A287GR24.1/122-224 Uncharacterized protein
{ECO:0000313|EnsemblPlants:HORVU1Hr1G091930.8}
MPSRVGADDADAGGSGGVGEIVEASGGVPPGNRSAARKTARFAEPVSAPCADDDGYG
DAVEVTIDIQDYTATVRSMEPIAGGKSSHGRSVIQNAKTRIKQVAEELQRFKSLSRRGW
NRGPSSIDRSVPARALEGLKFISKKTDGSDGWKWTDVEKCFREETKDGLLHRSKFAMC

IGMKERAGDVAFAGALFDALSRRRNISGDTINMEELRVFWKDISDTSFDSRLGIFFDMV
DKDANGVLDEKEVKDIITLSASANNLKMVAQKSEEYTRLIMEELDPKNRRYIEVAQLE
ALLTRPSNEWMQMGRMTISPKLSKLLSERQTDPNPLRRWYSRARYFIKDNWRRCWV
MLLWLSICVGLFAWKFEQYRHRAVFKVMGYCVCVAKGGAETLKFNMALTLLPVCRN
TITWLRSRTTVGQF
>A0A287KSQ2.1/134-232 Uncharacterized protein
{ECO:0000313|EnsemblPlants:HORVU3Hr1G037600.1}
MEMPDIEAGTVVTDSDSSRRPQDNTATTIPNSGSLEGSSHKTTKTTRFKDDGDDGVVEI
TLDIQRDSVSIQDVRPVAFDDSGSASAHSGALVSPSSSRGGKLSSKLRQVTNGLKLTNP
SKKVPPPTAPKTARKRYDRSKSSAAVALKGLQFVTAKVGNDGWTAVEKRFNHLQVD
GMLLRSRFGKCIGMEGSDEFAMQMFDSLARKRGMVKQVLTKEELKDFWEQLGDQGF
DNRLQTFFDIRVDKNADGRITSEEVKEIIALSASANKLSKIKERADEYTALIMEELDPNN
LGYIELEDLEALLLQSPSEAVARSTTTHSSKLSKALSMKLAPSNDTSPLHHHWQEFLYF
VEENWKRIWVVTLWLSICIALFVWKFIQYRNRAVFHIMGYCVATAKG
>A0A287KSR3.1/134-232 Uncharacterized protein
{ECO:0000313|EnsemblPlants:HORVU3Hr1G037600.17}
MEMPDIEAGTVVTDSDSSRRPQDNTATTIPNSGSLEGSSHKTTKTTRFKDDGDDGVVEI
TLDIQRDSVSIQDVRPVAFDDSGSASAHSGALVSPSSSRGGKLSSKLRQVTNGLKLTNP
SKKVPPPTAPKTARKRYDRSKSSAAVALKGLQFVTAKVGNDGWTAVEKRFNHLQVD
GMLLRSRFGKCIGMEGSDEFAMQMFDSLARKRGMVKQVLTKEELKDFWEQLGDQGF
DNRLQTFFDMVDKNADGRITSEEVKEIIALSASANKLSKIKERADEYTALIMEELDPNN
LGYIELEDLEALLLQSPSEAVARSTTTHSSKLSKALSMKLAPSNDTSPLHHHWQEFLYF
VEENWKRIWVVTLWLSICIALFVWKFIQYRNRAVFHIMGYCVATAKGAAETLKFNMA
LVLLPVCRNTITWIRSKTQIGAVVPFNDNINFHKVM
>A0A287KSR9.1/134-232 Uncharacterized protein
{ECO:0000313|EnsemblPlants:HORVU3Hr1G037600.11}
MEMPDIEAGTVVTDSDSSRRPQDNTATTIPNSGSLEGSSHKTTKTTRFKDDGDDGVVEI
TLDIQRDSVSIQDVRPVAFDDSGSASAHSGALVSPSSSRGGKLSSKLRQVTNGLKLTNP
SKKVPPPTAPKTARKRYDRSKSSAAVALKGLQFVTAKVGNDGWTAVEKRFNHLQVD
GMLLRSRFGKCIGMEGSDEFAMQMFDSLARKRGMVKQVLTKEELKDFWEQLGDQGF
DNRLQTFFDMVDKNADGRITSEEVKEIIALSASANKLSKIKERADEYTALIMEELDPNN
LGYIELEDLEALLLQSPSEAVARSTTTHSSKLSKALSMKLAPSNDTSPLHHHWQEFLYF
VEENWKRIWVVTLWLSICIALFVWKFIQYRNRAVFHIMGYCVATAKGAAETLKFNMA
LVLLPVCRNTITWIRSKTQIGAVVPFNDNINFHKVIAAGVAVGVALHAGAHLTC
>A0A287KSS4.1/134-232 Uncharacterized protein
{ECO:0000313|EnsemblPlants:HORVU3Hr1G037600.16}
MEMPDIEAGTVVTDSDSSRRPQDNTATTIPNSGSLEGSSHKTTKTTRFKDDGDDGVVEI
TLDIQRDSVSIQDVRPVAFDDSGSASAHSGALVSPSSSRGGKLSSKLRQVTNGLKLTNP
SKKVPPPTAPKTARKRYDRSKSSAAVALKGLQFVTAKVGNDGWTAVEKRFNHLQVD
GMLLRSRFGKCIGMEGSDEFAMQMFDSLARKRGMVKQVLTKEELKDFWEQLGDQGF
DNRLQTFFDMFDKNADGRITSEEVKEIIALSASANKLSKIKERADEYTALIMEELDPNNL
GYIELEDLEALLLQSPSEAVARSTTTHSSKLSKALSMKLAPSNDTSPLHHHWQEFLYFV
EENWKRIWVVTLWLSICIALFVWKFIQYRNRAVFHIMGYCVATAKGAAETLKFNMAL
VLLPVCRNTITWIRSKTQIGAVVPFNDNINFHKVIAAGVAVGVALHAG
>A0A287KST0.1/134-232 Uncharacterized protein
{ECO:0000313|EnsemblPlants:HORVU3Hr1G037600.10}

MEMPDIEAGTVVTDSDSSRRPQDNTATTIPNSGSLEGSSHKTTKTTRFKDDGDDGVVEI
TLDIQRDSVSIQDVRPVAFDDSGSASAHSGALVSPSSSRGGKLSSKLRQVTNGLKLTNP
SKKVPPPTAPKTARKRYDRSKSSAAVALKGLQFVTAKVGNDGWTAVEKRFNHLQVD
GMLLRSRFGKCIGMEGSDEFAMQMFDSLARKRGMVKQVLTKEELKDFWEQLGDQGF
DNRLQTFFDMG
>A0A287LIQ4.1/1-92 Predicted protein
{ECO:0000313|EnsemblPlants:HORVU3Hr1G069780.11}
GAHRALRGLRFISSNKASNAWREVQANFDRLARDGHLSRSDFAECIGMTESKEFALEL
FDTLSRRRQMKLDTISKEELREIWQQITDNSFDSRLQIFFDMVDKNADGRIGEAEVKEII
MLSASANKLSRLKEQAEEYAALIMEELDPEELGYIELWQLETLLLQKDTYVNYSQALS
YTSQALSQNLALRKRGSIRKIGNSLIYYLEDNWKRLWVLALWIGIMAGLFTWKFIQYR
ERYVFSVMGYCVTTAKGAAETLKLNMAIILLPVCRNTITWLRNTRAARVLPFDDNINF
HKTIAAAIVVGVILHAGNHLVCDFPRLIRSSEETYAPLGIYFGETKPTYLALIKGVEGITG
IIMVVCMIIAFTLATRWFRRSLVKLPKPFDKLTGFNAFWYSHHLFAIVYVALIVHGQCV
YLIRVWYRKSTWMYLAVPVCLYLGERILRFFRSGSYAVRLLKVAIYPGNVLTLQMTKP
ATFRYKSGQYMFVQCPAVSPFEWHPFSITSAPGDEYLSIHVRQLGDWTRELKRVFSAA
CEPPVSGKSGLLRADETTKKTLPKLLIDGPYGSPAQDYGKYDVLLLVGLGIGATPFISIL
KDLLNNIIKMEEEEDTSTDLYPPVGRNKPHVDLGTLMRVTTRPKKVLKTTNAYFYWV
TREQGSFDWFKGVMNEIAEMDQRNIIEMHNYLTSVYEEGDARSALITMLQALNHAKN
GVDVVSGTKVRTHFARPNWKKVLAKIASKHPYAKIGVFYCGAPVLAQELAKLCHEFN
GKCTTKFEFHKEYF
>A0A287LIQ6.1/1-81 Uncharacterized protein
{ECO:0000313|EnsemblPlants:HORVU3Hr1G069690.2}
ISSNKASNAWREVQANFDRLARDGHLSRSDFAECIGMTESKEFALELFDTLSRRRQMK
LDTISKEELREIWQQITDNSFDSRLQIFFDM
>A0A287LIR0.1/1-92 Predicted protein
{ECO:0000313|EnsemblPlants:HORVU3Hr1G069780.10}
GAHRALRGLRFISSNKASNAWREVQANFDRLARDGHLSRSDFAECIGMTESKEFALEL
FDTLSRRRQMKLDTISKEELREIWQQITDNSFDSRLQIESIRVDKNADGRIGEAEVKEIIM
LSASANKLSRLKEQAEEYAALIMEELDPEELGYIELWQLETLLLQKDTYVNYSQALSY
TSQALSQNLALRKRGSIRKIGNSLIYYLEDNWKRLWVLALWIGIMAGLFTWKFIQYRE
RYVFSVMGYCVTTAKGAAETLKLNMAIILLPVCRNTITWLRNTRAARVLPFDDNINFH
KTIAAAIVVGVILHAGNHLVCDFPRLIRSSEETYAPLGIYFGETKPTYLALIKGVEGITGII
MVVCMIIAFTLATRWFRRSLVKLPKPFDKLTGFNAFWYSHHLFAIVYVALIVHGQCVY
LIRVWYRKSTWMYLAVPVCLYLGERILRFFRSGSYAVRLLKVAIYPGNVLTLQMTKPA
TFRYKSGQYMFVQCPAVSPFEWHPFSITSAPGDEYLSIHVRQLGDWTRELKRVFSAAC
EPPVSGKSGLLRADETTKKTLPKLLIDGPYGSPAQDYGKYDVLLLVGLGIGATPFISILK
DLLNNIIKMEEEEDTSTDLYPPVGRNKPHVDLGTLMRVTTRPKKVLKTTNAYFYWVTR
EQGSFDWFKGVMNEIAEMDQRNIIEMHNYLTSVYEEGDARSALITMLQALNHAKNGV
DVVSGTKVRTHFARPNWKKVLAKIASKHPYAKIGVFYCGAPVLAQELAKLCHEFNGK
CTTKFEFHKEYF
>A0A287LIR3.1/69-167 Predicted protein
{ECO:0000313|EnsemblPlants:HORVU3Hr1G069780.13}
SMRRTSSYRLLQLSQELMAGARHLSHDLTKRFSRSHSHSRDDAHHHQHQPPSGIESAL
AARAARRQRAQLDRTRSGAHRALRGLRFISSNKASNAWREVQANFDRLARDGHLSRS
DFAECIGMTESKEFALELFDTLSRRRQMKLDTISKEELREIWQQITDNSFDSRLQIFFDM

VDKNADGRIGEAEVKEIIMLSASANKLSRLKEQAEEYAALIMEELDPEELGYIELWQLE
TLLLQKDTYVNYSQALSYTSQALSQNLALRKRGSIRKIGNSLIYYLEDNWKRLWVLAL
WIGIMAGLFTWKFIQYRERYVFSVMGYCVTTAKGAAETLKLNMAIILLPVCRNTITWL
RNTRAARVLPFDDNINFHKTIAAAIVVGVILHAGNHLVCDFPRLIRSSEETYAPLGIYFG
ETKPTYLALIKGVEGITGIIMVVCMIIAFTLATRWFRRSLVKLPKPFDKLTGFNAFWYSH
HLFAIVYVALIVHGQCVYLIRVWYRKSTWMYLAVPVCLYLGERILRFFRSGSYAVRLL
KVSPNTSET
>A0A287LIR4.1/1-34 Predicted protein
{ECO:0000313|EnsemblPlants:HORVU3Hr1G069780.20}
FDTLSRRRQMKLDTISKEELREIWQQITDNSFDSRLQIFFDMVDKNADGRIGEAEVKEII
MLSASANKLSRLKEQAEEYAALIMEELDPEELGYIELWQLETLLLQKDTYVNYSQALS
YTSQALSQNLALRKRGSIRKIGNSLIYYLEDNWKRLWVLALWIGIMAGLFTWKFIQYR
ERYVFSVMGYCVTTAKGAAETLKLNMAIILLPVCRNTITWLRNTRAARVLPFDDNINF
HKTIAAAIVVGVILHAGNHLVCDFPRLIRSSEETYAPLGIYFGETKPTYLALIKGVEGITG
IIMVVCMIIAFTLATRWFRRSLVKLPKPFDKLTGFNAFWYSHHLFAIVYVALIVHGQCV
YLIRVWYRKSTWMYLAVPVCLYLGERILRFFRSGSYAVRLLKVAIYPGNVLTLQMTKP
ATFRYKSGQYMFVQCPAVSPFEWHPFSITSAPGDEYLSIHVRQLGDWTRELKRVFSAA
CEPPVSGKSGLLRADETTKKTLPKLLIDGPYGSPAQDYGKYDVLLLVGLGIGATPFISIL
KDLLNNIIKMEEEERILLRTFIHQLVGISHMLIWVLL
>A0A287LIR6.1/2-100 Uncharacterized protein
{ECO:0000313|EnsemblPlants:HORVU3Hr1G069690.1}
AQLDRTRSGAHRALRGLRFISSNKASNAWREVQANFDRLARDGHLSRSDFAECIGMTE
SKEFALELFDTLSRRRQMKLDTISKEELREIWQQITDNSFDSRLQIFFDMVDKNADGRIG
EAE
>A0A287LIR8.1/1-68 Predicted protein
{ECO:0000313|EnsemblPlants:HORVU3Hr1G069780.18}
QANFDRLARDGHLSRSDFAECIGMTESKEFALELFDTLSRRRQMKLDTISKEELREIWQ
QITDNSFDSRLQIFFDMVDKNADGRIGEAEVKEIIMLSASANKLSRLKEQAEEYAALIM
EELDPEELGYIELWQLETLLLQKDTYVNYSQALSYTSQALSQNLALRKRGSIRKIGNSLI
YYLEDNWKRLWVLALWIGIMAGLFTWKFIQYRERYVFSVMGYCVTTAKGAAETLKL
NMAIILLPVCRNTITWLRNTRAARVLPFDDNINFHKTIAAAIVVGVILHAGNHLVCDFP
RLIRSSEETYAPLGIYFGETKPTYLALIKGVEGITGIIMVVCMIIAFTLATRWFRRSLVKL
PKPFDKLTGFNAFWYSHHLFAIVYVALIVHGQCVYLIRVWYRKSTWMYLAVPVCLYL
GERILRFFRSGSYAVRLLKVAIYPGNVLTLQMTKPATFRYKSGQYMFVQCPAVSPFE
>A0A287LIR9.1/4-54 Predicted protein
{ECO:0000313|EnsemblPlants:HORVU3Hr1G069780.5}
LRLRGMHRGMTESKEFALELFDTLSRRRQMKLDTISKEELREIWQQITDNSFDSRLQFE
SIRVDKNADGRIGEAEVKEIIMLSASANKLSRLKEQAEEYAALIMEELDPEELGYIELW
QLETLLLQKDTYVNYSQALSYTSQALSQNLALRKRGSIRKIGNSLIYYLEDNWKRLWV
LALWIGIMAGLFTWKFIQYRERYVFSVMGYCVTTAKGAAETLKLNMAIILLPVCRNTIT
WLRNTRAARVLPFDDNINFHKTIAAAIVVGVILHAGNHLVCDFPRLIRSSEETYAPLGIY
FGETKPTYLALIKGVEGITGIIMVVCMIIAFTLATRWFRRSLVKLPKPFDKLTGFNAFWY
SHHLFAIVYVALIVHGQCVYLIRVWYRKSTWMYLAVPVCLYLGERILRFFRSGSYAVR
LLKVAIYPGNVLTLQMTKPATFRYKSGQYMFVQCPAVSPFEWHPFSITSAPGDEYLSIH
VRQLGDWTRELKRVFSAACEPPVSGKSGLLRADETTKKTLPKLLIDGPYGSPAQDYGK
YDVLLLVGLGIGATPFISILKDLLNNIIKMEEEEDTSTDLYPPVGRNKPHVDLGTLMRVT

TRPKKVLKTTNAYFYWVTREQGSFDWFKGVMNEIAEMDQRNIIEMHNYLTSVYEEGD
ARSALITMLQALNHAKNGVDVVSGTKVRTHFARPNWKKVLAKIASKHPYAKIGVFYC
GAPVLAQELAKLCHEFNGKCTTKFEFHKEYF
>A0A287LIS1.1/1-88 Predicted protein
{ECO:0000313|EnsemblPlants:HORVU3Hr1G069780.22}
ALRGLRFISSNKASNAWREVQANFDRLARDGHLSRSDFAECIGMTESKEFALELFDTLS
RRRQMKLDTISKEELREIWQQITDNSFDSRLQIFFDMVDKNADGRIGEAEVKEIIMLSAS
ANKLSRLKEQAEEYAALIMEELDPEELGYIELWQLETLLLQKDTYVNYSQALSYTSQA
LSQNLALRKRGSIRKIGNSLIYYLEDNWKRLWVLALWIGIMAGLFTWKFIQYRERYVF
SVMGYCVTTAKGAAETLKLNMAIILLPVCRNTITWLRNTRAARVLPFDDNINFHKTIA
AAIVVGVILHAGNHLVCDFPRLIRSSEETYAPLGIYFGETKPTYLALIKGVEGITGIIMVV
CMIIAFTLATRWFRRSLVKLPKPFDKLTGFNAFWYSHHLFAIVYVALIVHGQCVYLIRV
WYRKSTWMYLAVPVCLYLGERILRFFRSGSYAVRLLKVAIYPGNVLTLQMTKPATFR
YKSGQYMFVQCPAVSPFEWYIITVHLWPSIIAELCVLVSTFRIDNT
>A0A287LIS7.1/1-91 Predicted protein
{ECO:0000313|EnsemblPlants:HORVU3Hr1G069780.7}
AHRALRGLRFISSNKASNAWREVQANFDRLARDGHLSRSDFAECIGMTESKEFALELF
DTLSRRRQMKLDTISKEELREIWQQITDNSFDSRLQIFFDIRVDKNADGRIGEAEVKEII
MLSASANKLSRLKEQAEEYAALIMEELDPEELGYIELWQLETLLLQKDTYVNYSQALS
YTSQALSQNLALRKRGSIRKIGNSLIYYLEDNWKRLWVLALWIGIMAGLFTWKFIQYR
ERYVFSVMGYCVTTAKGAAETLKLNMAIILLPVCRNTITWLRNTRAARVLPFDDNINF
HKTIAAAIVVGVILHAGNHLVCDFPRLIRSSEETYAPLGIYFGETKPTYLALIKGVEGITG
IIMVVCMIIAFTLATRWFRRSLVKLPKPFDKLTGFNAFWYSHHLFAIVYVALIVHGQCV
YLIRVWYRKSTWMYLAVPVCLYLGERILRFFRSGSYAVRLLKVAIYPGNVLTLQMTKP
ATFRYKSGQYMFVQCPAVSPFEWHPFSITSAPGDEYLSIHVRQLGDWTRELKRVFSAA
CEPPVSGKSGLLRADETTKKTLPKLLIDGPYGSPAQDYGKYDVLLLVGLGIGATPFISIL
KDLLNNIIKMEEEEDTSTDLYPPVGRNKPHVDLGTLMRVTTRPKKVLKTTNAYFYWV
TREQGSFDWFKGVMNEIAEMDQRNIIEMHNYLTSVYEEGDARSALITMLQALNHAKN
GVDVVSGTKVRTHFARPNWKKVLAKIASKHPYAKIGVFYCGAPVLAQELAKLCHEFN
GKCTTKFEFHKEYF
>A0A287LIT2.1/1-68 Predicted protein
{ECO:0000313|EnsemblPlants:HORVU3Hr1G069780.17}
QANFDRLARDGHLSRSDFAECIGMTESKEFALELFDTLSRRRQMKLDTISKEELREIWQ
QITDNSFDSRLQIFSIRVDKNADGRIGEAEVKEIIMLSASANKLSRLKEQAEEYAALIME
ELDPEELGYIELWQLETLLLQKDTYVNYSQALSYTSQALSQNLALRKRGSIRKIGNSLIY
YLEDNWKRLWVLALWIGIMAGLFTWKFIQYRERYVFSVMGYCVTTAKGAAETLKLN
MAIILLPVCRNTITWLRNTRAARVLPFDDNINFHKTIAAAIVVGVILHAGNHLVCDFPRL
IRSSEETYAPLGIYFGETKPTYLALIKGVEGITGIIMVVCMIIAFTLATRWFRRSLVKLPK
PFDKLTGFNAFWYSHHLFAIVYVALIVHGQCVYLIRVWYRKSTWMYLAVPVCLYLGE
RILRFFRSGSYAVRLLKVAIYPGNVLTLQMTKPATFRYKSGQYMFVQCPAVSPFEWHP
FSITSAPGDEYLSIHVRQLGDWTRELKRVFSAACEPPVSGKSGLLRADETTKKTLPKLLI
DGPYGSPAQDYGKYDVLLLVGLGIGATPFISILKDLLNNIIKMEEEEDTSTDLYPPVGRN
KPHVDLGTLMRVTTRPKKVLKTTNAYFYWVTREQGSFDWFKGVMNEIAEMDQRNIIE
MHNYLTSVYEEGDARSALITMLQALNHAKNGVDVVSGTKVRTHFARPNWKKVLAKI
ASKHPYAKIGVFYCGAPVLAQELAKLCHEFNGKCTTKFEFHKEYF

>A0A287LIT9.1/1-89 Predicted protein
{ECO:0000313|EnsemblPlants:HORVU3Hr1G069780.6}
RALRGLRFISSNKASNAWREVQANFDRLARDGHLSRSDFAECIGMTESKEFALELFDTL
SRRRQMKLDTISKEELREIWQQITDNSFDSRLQIFFDMVDKNADGRIGEAEVKEIIMLSA
SANKLSRLKEQAEEYAALIMEELDPEELGYIELWQLETLLLQKDTYVNYSQALSYTSQ
ALSQNLALRKRGSIRKIGNSLIYYLEDNWKRLWVLALWIGIMAGLFTWKFIQYRERYV
FSVMGYCVTTAKGAAETLKLNMAIILLPVCRNTITWLRNTRAARVLPFDDNINFHKTIA
AAIVVGVILHAGNHLVCDFPRLIRSSEETYAPLGIYFGETKPTYLALIKGVEGITGIIMVV
CMIIAFTLATRWFRRSLVKLPKPFDKLTGFNAFWYSHHLFAIVYVALIVHGQCVYLIRV
WYRKSTWMYLAVPVCLYLGERILRFFRSGSYAVRLLKVAIYPGNVLTLQMTKPATFR
YKSGQYMFVQCPAVSPFEWHPFSITSAPGDEYLSIHVRQLGDWTRELKRVFSAACEPP
VSGKSGLLRADETTKKTLPKLLIDGPYGSPAQDYGKYDVLLLVGLGIGATPFISILKDLL
NNIIKMEEEEDTSTDLYPPVGRNKPHVDLGTLMRVTTRPKKVLKTTNAYFYWVTREQ
GSFDWFKGVMNEIAEMDQRNIIEMHNYLTSVYEEGDARSALITMLQALNHAKNGVDV
VSGTKVRTHFARPNWKKVLAKIASKHPYAKIGVFYCGAPVLAQELAKLCHEFNGKCT
TKFEFHKEYF
>A0A287M3V5.1/44-145 Uncharacterized protein
{ECO:0000313|EnsemblPlants:HORVU3Hr1G087210.10}
PRPPGFRGLMQQPSRLASGVRQFASRVSMKVPEVVPGIRPGGGRMTRMQSSAQMGLK
GLRFLDKTSGSKEGWKAVERRFDEMSKASGRLPKESFGKCIGMGDSKEFAGELFVTLS
RRRSIEPEQGITKEQLREFWTEMTDQNFDSRLRIFFDMCDKNGDGMLTEDEVKEVIILS
ASANKLAKLKSHAATYSSLIMEELDPDDRGYIEIWQLETLLRGMVSAQAPEVKLKRTT
SSLARTMIPMRYRSPLKRHVTRTMDFIHENWKRIWLVTLWLAANLALFVYKFEQYKH
RSSFQVMGNCVCIAKGAAETLKLNMALILLPVCRNTLTTLRSTALSHVIPFDDNINFHK
VLAGAIAVGTVVHTLAHVTCDFPRLISCPSDKFMALLGPNFGFRQPTYPDLLASAPGVT
GILMIIIMSFSFTLAMHTFRRSVVKLPSPLHHLAGFNAFWYAHHLLLLVYVLLVVHSYFI
FLTRVWYKKTVIKSSALLTYIYLGFFPVNSNVDDRYLFTQTWMFLIVPVLFYACERIIRK
VRENNYHVNILKVGY
>A0A287M3W0.1/71-172 Uncharacterized protein
{ECO:0000313|EnsemblPlants:HORVU3Hr1G087210.1}
MADRPAPPLDGITVDGGGRTPPAGPGLPRPPGFRGLMQQPSRLASGVRQFASRVSMKV
PEVVPGIRPGGGRMTRMQSSAQMGLKGLRFLDKTSGSKEGWKAVERRFDEMSKASGR
LPKESFGKCIGMGDSKEFAGELFVTLSRRRSIEPEQGITKEQLREFWTEMTDQNFDSRLR
IFFDMCDKNGDGMLTEDEVKEVIILSASANKLAKLKSHAATYSSLIMEELDPDDRGYIE
IWQLETLLRGMVSAQAPEVKLKRTTSSLARTMIPMRYRSPLKRHVTRTMDFIHENWKR
IWLVTLWLAANLALFVYKFEQYKHRSSFQVMGNCVCIAKGAAETLKLNMALILLPVY
VLLVVHSYFIFLTRVWYKKTTWMFLIVPVLFYACERIIRKVRENNYHVNILKAAIYPGN
VLSLHMKKPPGFKYKSGMYLFVKCPDVSPFEWHPFSITSAPGDDYLSVHIRTLGDWTS
ELRNLFGKCCEAQVTSKKATLSRLETTVVADSTTEDTRFPKVFIDGPYGAPAQNYKKY
DILLLIGLGIGATPFISILKDLLNNIKSNDEVESIHGSEIGSFKNSGPGRAYFYWVTREQGS
FDWFKGVMNEVADNDHSNVIEMHNYLTSVYEEGDARSALIAMVQSLQHAKNGVDIV
SGSKIRTHFARPNWRKVFSDLANAHKNSRIGVFYCGSPTLTKQLKDLSKEFSQTTTTRF
HFHKENF
>A0A287M3W6.1/38-139 Uncharacterized protein
{ECO:0000313|EnsemblPlants:HORVU3Hr1G087210.14}

RGLMQQPSRLASGVRQFASRVSMKVPEVVPGIRPGGGRMTRMQSSAQMGLKGLRFLD
KTSGSKEGWKAVERRFDEMSKASGRLPKESFGKCIGMGDSKEFAGELFVTLSRRRSIEP
EQGITKEQLREFWTEMTDQNFDSRLRIFFDMCDKNGDGMLTEDEVKEVIILSASANKL
AKLKSHAATYSSLIMEELDPDDRGYIEIWQLETLLRGMVSAQAPEVKLKRTTSSLART
MIPMRYRSPLKRHVTRTMDFIHENWKRIWLVTLWLAANLALFVYKFEQYKHRSSFQV
MGNCVCIAKGAAETLKLNMALILLPVCRNTLTTLRSTALSHVIPFDDNINFHKVLAGAI
AVGTVVHTLAHVTCDFPRLISCPSDKFMALLGPNFGFRQPTYPDLLASAPGVTGILMIII
MSFSFTLAMHTFRRSVVKLPSPLHHLAGFNAFWYAHHLLLLVYVLLVVHSYFIFLTRV
WYKKTTWMFLIVPVLFYACERIIRKVRENNYHVNILKAAIYPGNVLSLHMKKPPGFKY
KSGMYLFVKCPDVSPFEWHPFSITSAPGDDYLSVHIRTLGDWTSELRNLFGKCCEAQV
TSKKATLSRLETTVVADSTTEDTRFPKVFIDGPYGAPAQNYKKYDILLLIGLGIGATPFIS
ILKDLLNNIKSNDEVESIHGSEIGSFKNSGPGRAYFYWVTREQGSFDWFKGVMNEVAD
NDHSNVIEMHNYLTSVYEEGDARSALIAMVQSLQHAKNGVDIVSGSKVFSQLCILICNP
ICPCLLF
>A0A287M3W9.1/1-46 Uncharacterized protein
{ECO:0000313|EnsemblPlants:HORVU3Hr1G087210.24}
MGDSKEFAGELFVTLSRRRSIEPEQGITKEQLREFWTEMTDQNFDSRLRIFFDMCDKNG
DGMLTEDEVKEVIILSASANKLAKLKSHAATYSSLIMEELDPDDRGYIEIWQLETLLRG
MVSAQAPEVKLKRTTSSLARTMIPMRYRSPLKRHVTRTMDFIHENWKRIWLVTLWLA
ANLALFVYKFEQYKHRSSFQVMGNCVCIAKGAAETLKLNMALILLPVCRNTLTTLRST
ALSHVIPFDDNINFHKVLAGAIAVGTVVHTLAHVTCDFPRLISCPSDKFMALLGPNFGF
RQPTYPDLLASAPGVTGILMIIIMSFSFTLAMHTFRRSVVKLPSPLHHLAGFNAFWYAH
HLLLLVYVLLVVHSYFIFLTRVWYKKTTWMFLIVPVLFYACERIIRKVRENNYHVNILK
AAIYPGNVLSLHMKKPPGFKYKSGMYLFVKCPDVSPFEWHPFSITSAPGDDYLSVHIRT
LGDWTSELRNLFGKCCEAQVTSKKATLSRLETTVVADSTTEDTRFPKVFIDGPYGAPA
QNYKKYDILLLIGLGIGATPFISILKDLLNNIKSNDEVESIHGSEIGSFKNSGPGRAYFYW
VTREQGSFDWFKGVMNEVADNDHSNVIEMHNYLTSVYEEGDARSALIAMVQSLQHA
KNGVDIVSGSKIRTHFARPNWRKVFSDLANAHKNSRIGVFYCGSPTLTKQLKDLSKEFS
QTTTTRFHFHKENF
>A0A287M3X0.1/26-127 Uncharacterized protein
{ECO:0000313|EnsemblPlants:HORVU3Hr1G087210.19}
GVRQFASRVSMKVPEVVPGIRPGGGRMTRMQSSAQMGLKGLRFLDKTSGSKEGWKA
VERRFDEMSKASGRLPKESFGKCIGMGDSKEFAGELFVTLSRRRSIEPEQGITKEQLREF
WTEMTDQNFDSRLRIFFDMCDKNGDGMLTEDEVKEVIILSASANKLAKLKSHAATYSS
LIMEELDPDDRGYIEIWQLETLLRGMVSAQAPEVKLKRTTSSLARTMIPMRYRSPLKRH
VTRTMDFIHENWKRIWLVTLWLAANLALFVYKFEQYKHRSSFQVMGNCVCIAKGAA
ETLKLNMALILLPVCRNTLTTLRSTALSHVIPFDDNINFHKVLAGAIAVGTVVHTLAHV
TCDFPRLISCPSDKFMALLGPNFGFRQPTYPDLLASAPGVTGILMIIIMSFSFTLAMHTFR
RSVVKLPSPLHHLAGFNAFWYAHHLLLLVYVLLVVHSYFIFLTRVWYKKTTWMFLIVP
VLFYACERIIRKVRENNYHVNILKAAIYPGNVLSLHMKKPPGFKYKSGMYLFVKCPDV
SPFEWHPFSITSAPGDDYLSVHIRTLGDWTSELRNLFGKCCEAQVTSKKATLSRLETTV
VADSTTEDTRFPKVFIDGPYGAPAQNYKKYDILLLIGLGIGATPFISILKDLLNNIKSNDE
VESIHGSEIGSFKNSGPGRAYFYWVTREQGSFDWFKGVMNEVADNDHSNVIEMHNYL
TSVYEEGDARSALIAMVQSLQHAKNGVDIVSGSKIRTHFARPNWRKVFSDLANAHKNS
RIGVFSIVDLQHSQNNSRIFQKNSAKQPQLGSISTRRTSKTVRKEFQTVCRYTCIGKEI

>A0A287M3X3.1/33-134 Uncharacterized protein
{ECO:0000313|EnsemblPlants:HORVU3Hr1G087210.17}
QPSRLASGVRQFASRVSMKVPEVVPGIRPGGGRMTRMQSSAQMGLKGLRFLDKTSGS
KEGWKAVERRFDEMSKASGRLPKESFGKCIGMGDSKEFAGELFVTLSRRRSIEPEQGIT
KEQLREFWTEMTDQNFDSRLRIFFDMCDKNGDGMLTEDEVKEVIILSASANKLAKLKS
HAATYSSLIMEELDPDDRGYIEIWQLETLLRGMVSAQAPEVKLKRTTSSLARTMIPMRY
RSPLKRHVTRTMDFIHENWKRIWLVTLWLAANLALFVYKFEQYKHRSSFQVMGNCVC
IAKGAAETLKLNMALILLPVCRNTLTTLRSTALSHVIPFDDNINFHKVLAGAIAVGTVV
HTLAHVTCDFPRLISCPSDKFMALLGPNFGFRQPTYPDLLASAPGVTGILMIIIMSFSFTL
AMHTFRRSVVKLPSPLHHLAGFNAFWYAHHLLLLVYVLLVVHSYFIFLTRVWYKKTT
WMFLIVPVLFYACERIIRKVRENNYHVNILKAAIYPGNVLSLHMKKPPGFKYKSGMYL
FVKCPDVSPFEWHPFSITSAPGDDYLSVHIRTLGDWTSELRNLFGKCCEAQVTSKKATL
SRLETTVVADSTTEDTRFPKVFIDGPYGAPAQNYKKYDILLLIGLGIGATPFISILKDLLN
NIKSNDEVESIHGSEIGSFKNSGPGRAYFYWVTREQGSFDWFKGVMNEVADNDHSNVI
EMHNYLTSVYEEGDARSALIAMVQSLQHAKNGVDIVSGSKIRTHFARPNWRKVFSDL
ANAHKNSRIGGVFYCGSPTLTKQLKDLSKEFSQTTTTRFHFHKENF
>A0A287M3X4.1/64-165 Uncharacterized protein
{ECO:0000313|EnsemblPlants:HORVU3Hr1G087210.4}
PLDGITVDGGGRTPPAGPGLPRPPGFRGLMQQPSRLASGVRQFASRVSMKVPEVVPGIR
PGGGRMTRMQSSAQMGLKGLRFLDKTSGSKEGWKAVERRFDEMSKASGRLPKESFG
KCIGMGDSKEFAGELFVTLSRRRSIEPEQGITKEQLREFWTEMTDQNFDSRLRIFFDMC
DKNGDGMLTEDEVKEVIILSASANKLAKLKSHAATYSSLIMEELDPDDRGYIEIWQLET
LLRGMVSAQAPEVKLKRTTSSLARTMIPMRYRSPLKRHVTRTMDFIHENWKRIWLVTL
WLAANLALFVYKFEQYKHRSSFQVMGNCVCIAKGAAETLKLNMALILLPVCRNTLTT
LRSTALSHVIPFDDNINFHKVLAGAIAVGTVVHTLAHVTCDFPRLISCPSDKFMALLGP
NFGFRQPTYPDLLASAPGVTGILMIIIMSFSFTLAMHTFRRSVVKLPSPLHHLAGFNAFW
YAHHLLLLVYVLLVVHSYFIFLTRVWYKKTVIKSSALLTYIYLGFFPVNSNVDDRYLFT
QTWMFLIVPVLFYACERIIRKVRENNYHVNILKAAIYPGNVLSLHMKKPPGFKYKSGM
YLFVKCPDVSPFEWHPFSITSAPGDDYLSVHIRTLGDWTSELRNLFGKCCEAQVTSKKA
TLSRLETTVVADSTTEDTRFPKVFIDGPYGAPAQNYKKYDILLLIGLGIGATPFISILKDL
LNNIKSNDEVESIHGSEIGSFKNSGPGRAYFYWVTREQGSFDWFKGVMNEVADNDHSN
VIEMHNYLTSVYEEGDARSALIAMVQSLQHAKNGVDIVSGSKIRTHFARPNWRKVFSD
LANAHKNSRIGVFYCGSPTLTKQLKDLSKEFSQTTTTRFHFHKENF
>A0A287M3X8.1/24-125 Uncharacterized protein
{ECO:0000313|EnsemblPlants:HORVU3Hr1G087210.21}
RQFASRVSMKVPEVVPGIRPGGGRMTRMQSSAQMGLKGLRFLDKTSGSKEGWKAVE
RRFDEMSKASGRLPKESFGKCIGMGDSKEFAGELFVTLSRRRSIEPEQGITKEQLREFWT
EMTDQNFDSRLRIFFDMCDKNGDGMLTEDEVKEVIILSASANKLAKLKSHAATYSSLI
MEELDPDDRGYIEIWQLETLLRGMVSAQAPEVKLKRTTSSLARTMIPMRYRSPLKRHV
TRTMDFIHENWKRIWLVTLWLAANLALFVYKFEQYKHRSSFQVMGNCVCIAKGAAET
LKLNMALILLPVCRNTLTTLRSTALSHVIPFDDNINFHKVLAGAIAVGTVVHTLAHVTC
DFPRLISCPSDKFMALLGPNFGFRQPTYPDLLASAPGVTGILMIIIMSFSFTLAMHTFRRS
VVKLPSPLHHLAGFNAFWYAHHLLLLVYVLLVVHSYFIFLTRVWYKKTTWMFLIVPV
LFYACERIIRKVRENNYHVNILKAAIYPGNVLSLHMKKPPGFKYKSGMYLFVKCPDVS
PFEWHPFSITSAPGDDYLSVHIRTLGDWTSELRNLFGKCCEAQVTSKKATLSRLETTVV
ADSTTEDTR

>A0A287M3X9.1/44-145 Uncharacterized protein
{ECO:0000313|EnsemblPlants:HORVU3Hr1G087210.9}
PRPPGFRGLMQQPSRLASGVRQFASRVSMKVPEVVPGIRPGGGRMTRMQSSAQMGLK
GLRFLDKTSGSKEGWKAVERRFDEMSKASGRLPKESFGKCIGMGDSKEFAGELFVTLS
RRRSIEPEQGITKEQLREFWTEMTDQNFDSRLRIFFDMCDKNGDGMLTEDEVKEVIILS
ASANKLAKLKSHAATYSSLIMEELDPDDRGYIEIWQLETLLRGMVSAQAPEVKLKRTT
SSLARTMIPMRYRSPLKRHVTRTMDFIHENWKRIWLVTLWLAANLALFVYKFEQYKH
RSSFQVMGNCVCIAKGAAETLKLNMALILLPVCRNTLTTLRSTALSHVIPFDDNINFHK
VLAGAIAVGTVVHTLAHVTCDFPRLISCPSDKFMALLGPNFGFRQPTYPDLLASAPGVT
GILMIIIMSFSFTLAMHTFRRSVVKLPSPLHHLAGFNAFWYAHHLLLLVYVLLVVHSYFI
FLTRVWYKKTTWMFLIVPVLFYACERIIRKVRENNYHVNILKAAIYPGNVLSLHMKKP
PGFKYKSGMYLFVKCPDVSPFEWHPFSITSAPGDDYLSVHIRTLGDWTSELRNLFGKCC
EAQVTSKKATLSRLETTVVADSTTEDTRFPKVFIDGPYGAPAQNYKKYDILLLIGLGIG
ATPFISILKDLLNNIKSNDEVESIHGSEIGSFKNSGPGRAYFYWVTREQGSFDWFKGVM
NEVADNDHSNVIEMHNYLTSVYEEGDARSALIAMVQSLQHAKNGVDIVSGSKIRTHFA
RPNWRKVFSDLANAHKNSRIGVFYCGSPTLTKQLKDLSKEFSQTTTTRFHFHKENF
>A0A287M3Y0.1/53-154 Uncharacterized protein
{ECO:0000313|EnsemblPlants:HORVU3Hr1G087210.7}
RTPPAGPGLPRPPGFRGLMQQPSRLASGVRQFASRVSMKVPEVVPGIRPGGGRMTRMQ
SSAQMGLKGLRFLDKTSGSKEGWKAVERRFDEMSKASGRLPKESFGKCIGMGDSKEF
AGELFVTLSRRRSIEPEQGITKEQLREFWTEMTDQNFDSRLRIFFDMCDKNGDGMLTED
EVKEVIILSASANKLAKLKSHAATYSSLIMEELDPDDRGYIEIWQLETLLRGMVSAQAP
EVKLKRTTSSLARTMIPMRYRSPLKRHVTRTMDFIHENWKRIWLVTLWLAANLALFV
YKFEQYKHRSSFQVMGNCVCIAKGAAETLKLNMALILLPVCRNTLTTLRSTALSHVIPF
DDNINFHKVLAGAIAVGTVVHTLAHVTCDFPRLISCPSDKFMALLGPNFGFRQPTYPDL
LASAPGVTGILMIIIMSFSFTLAMHTFRRSVVKLPSPLHHLAGFNAFWYAHHLLLLVYV
LLVVHSYFIFLTRVWYKKTTWMFLIVPVLFYACERIIRKVRENNYHVNILKAAIYPGNV
LSLHMKKPPGFKYKSGMYLFVKCPDVSPFEWHPFSITSAPGDDYLSVHIRTLGDWTSEL
RNLFGKCCEAQVTSKKATLSRLETTVVADSTTEDTRFPKVFIDGPYGAPAQNYKKYDI
LLLIGLGIGATPFISILKDLLNNIKSNDVNFFRCKVFSTLYTYYS
>A0A287M3Y1.1/71-172 Uncharacterized protein
{ECO:0000313|EnsemblPlants:HORVU3Hr1G087210.3}
MADRPAPPLDGITVDGGGRTPPAGPGLPRPPGFRGLMQQPSRLASGVRQFASRVSMKV
PEVVPGIRPGGGRMTRMQSSAQMGLKGLRFLDKTSGSKEGWKAVERRFDEMSKASGR
LPKESFGKCIGMGDSKEFAGELFVTLSRRRSIEPEQGITKEQLREFWTEMTDQNFDSRLR
IFFDMCDKNGDGMLTEDEVKEVIILSASANKLAKLKSHAATYSSLIMEELDPDDRGYIE
IWQLETLLRGMVSAQAPEVKLKRTTSSLARTMIPMRYRSPLKRHVTRTMDFIHENWKR
IWLVTLWLAANLALFVYKFEQYKHRSSFQVMGNCVCIAKGAAETLKLNMALILLPVC
RNTLTTLRSTALSHVIPFDDNINFHKVLAGAIAVGTVVHTLAHVTCDFPRLISCPSDKFM
ALLGPNFGFRQPTYPDLLASAPGVTGILMIIIMSFSFTLAMHTFRRSVVKLPSPLHHLAG
FNAFWYAHHLLLLVYVLLVVHSYFIFLTRVWYKKTTWMFLIVPVLFYACERIIRKVRE
NNYHVNILKLRII
>A0A287M3Y2.1/26-127 Uncharacterized protein
{ECO:0000313|EnsemblPlants:HORVU3Hr1G087210.20}
GVRQFASRVSMKVPEVVPGIRPGGGRMTRMQSSAQMGLKGLRFLDKTSGSKEGWKA
VERRFDEMSKASGRLPKESFGKCIGMGDSKEFAGELFVTLSRRRSIEPEQGITKEQLREF

WTEMTDQNFDSRLRIFFDIRCDKNGDGMLTEDEVKEVIILSASANKLAKLKSHAATYSS
LIMEELDPDDRGYIEIWQLETLLRGMVSAQAPEVKLKRTTSSLARTMIPMRYRSPLKRH
VTRTMDFIHENWKRIWLVTLWLAANLALFVYKFEQYKHRSSFQVMGNCVCIAKGAA
ETLKLNMALILLPVCRNTLTTLRSTALSHVIPFDDNINFHKVLAGAIAVGTVVHTLAHV
TCDFPRLISCPSDKFMALLGPNFGFRQPTYPDLLASAPGVTGILMIIIMSFSFTLAMHTFR
RSVVKLPSPLHHLAGFNAFWYAHHLLLLVYVLLVVHSYFIFLTRVWYKKTDMDVLDS
PCPLLRLRENYQKSPREQLSCEHSQGCDLPRKRALSSHEEATGFQVQEWDVPVCEMP
>A0A287M3Y5.1/53-154 Uncharacterized protein
{ECO:0000313|EnsemblPlants:HORVU3Hr1G087210.8}
RTPPAGPGLPRPPGFRGLMQQPSRLASGVRQFASRVSMKVPEVVPGIRPGGGRMTRMQ
SSAQMGLKGLRFLDKTSGSKEGWKAVERRFDEMSKASGRLPKESFGKCIGMGDSKEF
AGELFVTLSRRRSIEPEQGITKEQLREFWTEMTDQNFDSRLRIFFDMCDKNGDGMLTED
EVKEVIILSASANKLAKLKSHAATYSSLIMEELDPDDRGYIEIWQLETLLRGMVSAQAP
EVKLKRTTSSLARTMIPMRYRSPLKRHVTRTMDFIHENWKRIWLVTLWLAANLALFV
YKFEQYKHRSSFQVMGNCVCIAKGAAETLKLNMALILLPVCRNTLTTLRSTALSHVIPF
DDNINFHKVLAGAIAVGTVVHTLAHVTCDFPRLISCPSDKFMALLGPNFGFRQPTYPDL
LASAPGVTGILMIIIMSFSFTLAMHTFRRSVVKLPSPLHHLAGFNAFWYAHHLLLLVYV
LLVVHSYFIFLTRVWYKKTTWMFLIVPVLFYACERIIRKVRENNYHVNILKAAIYPGNV
LSLHMKKPPGFKYKSGMYLFVKCPDVSPFEWHPFSITSAPGDDYLSVHIRTLGDWTSEL
RNLFGKCCEAQVTSKKATLSRLETTVVADSTTEDTRFPKVFIDGPYGAPAQNYKKYDI
LLLIGLGIGATPFISILKDLLNNIKSNDEVESIHGSEIGSFKNSGPGRAYFYWVTREQGSF
DWFKGVMNEVADNDHSNVIEMHNYLTSVYEEGDARSALIAMVQSLQHAKNGVDIVS
GSKIRTHFARPNWRKVFSDLANAHKNSRIGVFYCGSPTLTKQLKDLSKEFSQTTTTRFH
FHKENF
>A0A287M3Y8.1/53-154 Uncharacterized protein
{ECO:0000313|EnsemblPlants:HORVU3Hr1G087210.6}
RTPPAGPGLPRPPGFRGLMQQPSRLASGVRQFASRVSMKVPEVVPGIRPGGGRMTRMQ
SSAQMGLKGLRFLDKTSGSKEGWKAVERRFDEMSKASGRLPKESFGKCIGMGDSKEF
AGELFVTLSRRRSIEPEQGITKEQLREFWTEMTDQNFDSRLRIFFDIRCDKNGDGMLTE
DEVKEVIILSASANKLAKLKSHAATYSSLIMEELDPDDRGYIEIWQLETLLRGMVSAQA
PEVKLKRTTSSLARTMIPMRYRSPLKRHVTRTMDFIHENWKRIWLVTLWLAANLALFV
YKFEQYKHRSSFQVMGNCVCIAKGAAETLKLNMALILLPVCRNTLTTLRSTALSHVIPF
DDNINFHKVLAGAIAVGTVVHTLAHVTCDFPRLISCPSDKFMALLGPNFGFRQPTYPDL
LASAPGVTGILMIIIMSFSFTLAMHTFRRSVVKLPSPLHHLAGFNAFWYAHHLLLLVYV
LLVVHSYFIFLTRVWYKKTTWMFLIVPVLFYACERIIRKVRENNYHVNILKAAIYPGNV
LSLHMKKPPGFKYKSGMYLFVKCPDVSPFEWHPFSITSAPGDDYLSVHIRTLGDWTSEL
RNLFGKCCEAQVTSKKATLSRLETTVVADSTTEDTRFPKVFIDGPYGAPAQNYKKYDI
LLLIGLGIGATPFISILKDLLNNIKSNDEVESIHGSEIGSFKNSGPGRAYFYWVTREQGSF
DWFKGVMNEVADNDHSNVIEMHNYLTSVYEEGDARSALIAMVQSLQHAKNGVDIVS
GSKIRTHFARPNWRKVFSDLANAHKNSRIGVFYCGSPTLTKQLKDLSKEFSQTTTTRFH
FHKENF
>A0A287M3Z0.1/59-160 Uncharacterized protein
{ECO:0000313|EnsemblPlants:HORVU3Hr1G087210.5}
TVDGGGRTPPAGPGLPRPPGFRGLMQQPSRLASGVRQFASRVSMKVPEVVPGIRPGGG
RMTRMQSSAQMGLKGLRFLDKTSGSKEGWKAVERRFDEMSKASGRLPKESFGKCIG
MGDSKEFAGELFVTLSRRRSIEPEQGITKEQLREFWTEMTDQNFDSRLRIFFDMCDKNG

DGMLTEDEVKEVIILSASANKLAKLKSHAATYSSLIMEELDPDDRGYIEIWQLETLLRG
MVSAQAPEVKLKRTTSSLARTMIPMRYRSPLKRHVTRTMDFIHENWKRIWLVTLWLA
ANLALFVYKFEQYKHRSSFQVMGNCVCIAKGAAETLKLNMALILLPVCRNTLTTLRST
ALSHVIPFDDNINFHKVLAGAIAVGTVVHTLAHVTCDFPRLISCPSDKFMALLGPNFGF
RQPTYPDLLASAPGVTGILMIIIMSFSFTLAMHTFRRSVVKLPSPLHHLAGFNAFWYAH
HLLLLVYVLLVVHSYFIFLTRVWYKKTTWMFLIVPVLFYACERIIRKVRENNYHVNILK
AAIYPGNVLSLHMKKPPGFKYKSGMYLFVKCPDVSPFEWHPFSITSAPGDDYLSVHIRT
LGDWTSELRNLFGKCCEAQVTSKKATLSRLETTVVADSTTEDTRFPKVFIDGPYGAPA
QNYKKYDILLLIGLGIGATPFISILKDLLNNIKSNDEVESIHGSEIGSFKNSGPGRAYFYW
VTREQGSFDWFKGVMNEVADNDHSNVIEMHNYLTSVYEEGDARSALIAMVQSLQHA
KNGVDIVSGSKIRTHFARPNWRKVFSDLANAHKNSRIGVFYCGSPTLTKQLKDLSKEFS
QTTTTRFHFHKENF
>A0A287PUR2.1/2-76 Uncharacterized protein
{ECO:0000313|EnsemblPlants:HORVU4Hr1G081670.2}
KGKDREATAAKRFDDLAENGLLHRSKFGKCIGMKELAFAGELFDALARRRNIAGDSIS
KAELLEFWDQISDTSFDSRLQTFFDMVDKDADGRITEEEVKEIITLSATANNLTKVKDQ
SEEYARLIMEELDPNNLGYIELYNLEMLLLQAPSQSMAIGTTNSRNLSQMLSQHLRPTA
EPNPLRRWYRRVSYFLEDNWRRCWVLLLWFCICVGLFTWKFMQYRERAVFKVMGYC
VCVAKGGAEMLKFNMALILLPVCRNTITWFRNRTAAGRFVPFDDNINFHKVIAAGISV
GAGLHIISHLTCDFPRLLRATEEEYEPMKRFFGEEQPPNYWWFVKGTEGWTGLVMLVL
MAIAFTLATPWFRRGRLSLPKPLNRLTGFNAFWYSHHLFIIVYALLIVHGHFLYLTKKW
QKKSTWMYLAVPMILYACERLTRALRSSVRPVKILKVAVYPGNVLSLHFSKPQGFRYK
SGQYIFVNCAAVSPFQWHPFSITSAPQDDYVSVHIRTLGDWTRELKNVFSKVCRPPTEG
KSGLLRAEYDRDGAMSNPSFPKVLIDGPYGAPAQDYKQYDIVLLVGLGIGATPMISIIK
DIINNMKRLEGDVESGNPGDASTSTSFRTRRAYFYWVTREQGSFEWFRGVMDEIAESD
KKGVIELHNYCTSVYEDGDARSALIAMLQSLNHAKNGVDIVSGTRVKTHFARPNWRN
VYKRIALNHREQRVGVFYCGAPVLTKELRELAQDFSRKTNTKFEFHKENF
>A0A287PUR6.1/5-102 Uncharacterized protein
{ECO:0000313|EnsemblPlants:HORVU4Hr1G081670.3}
GGPGRVDKSKSAAAHALKGLKFISRTDGSAGWPAVEKRFDDLAENGLLHRSKFGKCI
GMKELAFAGELFDALARRRNIAGDSISKAELLEFWDQISDTSFDSRLQTFFDMVDKDA
DGRITEEEVKEIITLSATANNLTKVKDQSEEYARLIMEELDPNNLGYIELYNLEMLLLQA
PSQSMAIGTTNSRNLSQMLSQHLRPTAEPNPLRRWYRRVSYFLEDNWRRCWVLLLWF
CICVGLFTWKFMQYRERAVFKVMGYCVCVAKGGAEMLKFNMALILLPVCRNTITWF
RNRTAAGRFVPFDDNINFHKVIAAGISVGAGLHIISHLTCDFPRLLRATEEEYEPMKRFF
GEEQPPNYWWFVKGTEGWTGLVMLVLMAIAFTLATPWFRRGRLSLPKPLNRLTGFNA
FWYSHHLFIIVYALLIVHGHFLYLTKKWQKKSVRNSNASP
>A0A287PUS9.1/208-305 Uncharacterized protein
{ECO:0000313|EnsemblPlants:HORVU4Hr1G081670.1}
ERRRPIHSRIAHQFQSAEEKRKGKDREATAAKRTQISSSVCPTILSSYPASGEPGPAAGA
AMHNRAGGGGAGEIVEAGERVVPHSGPLGGKRSAMRKSARFAESVSAPLTAPHGAPR
GGGGNDDDDDYVEITLDVRDDSVAVHSVKPAAGGEDSDVKLLAQTLEKRSSSYGQG
VLRNASTRIKQVSQELRRLASVNRRGGGGAGGPGRVDKSKSAAAHALKGLKFISRTDG
SAGWPAVEKRFDDLAENGLLHRSKFGKCIGMKELAFAGELFDALARRRNIAGDSISKA
ELLEFWDQISDTSFDSRLQTFFDMVDKDADGRITEEEVKEIITLSATANNLTKVKDQSEE
YARLIMEELDPNNLGYIELYNLEMLLLQAPSQSMAIGTTNSRNLSQMLSQHLRPTAEPN

PLRRWYRRVSYFLEDNWRRCWVLLLWFCICVGLFTWKFMQYRERAVFKVMGYCVC
VAKGGAEMLKFNMALILLPVCRNTITWFRNRTAAGRFVPFDDNINFHKVIAAGISVGA
GLHIISHLTCDFPRLLRATEEEYEPMKRFFGEEQPPNYWWFVKGTEGWTGLVMLVLM
AIAFTLATPWFRRGRLSLPKPLNRLTGFNAFWYSHHLFIIVYALLIVHGHFLYLTKKWQ
KKSTWMYLAVPMILYACERLTRALRSSVRPVKILKVAVYPGNVLSLHFSKPQGFRYKS
GQYIFVNCAAVSPFQWHPFSITSAPQDDYVSVHIRTLGDWTRELKNVFSKVCRPPTEGK
SGLLRAEYDRDGAMSNPSFPKVLIDGPYGAPAQDYKQYDIVLLVGLGIGATPMISIIKDI
INNMKRLEGDVESGNPGDASTSTSFRTRRAYFYWVTREQGSFEWFRGVMDEIAESDK
KGVIELHNYCTSVYEDGDARSALIAMLQSLNHAKNGVDIVSGTRVKTHFARPNWRNV
YKRIALNHREQRVGVFYCGAPVLTKELRELAQDFSRKTNTKFEFHKENF
>A0A287PZF9.1/75-173 Predicted protein
{ECO:0000313|EnsemblPlants:HORVU4Hr1G086500.14}
MADIEAGKPATESDTATLIPNSGSLEGSSRATKTTRFKDDDEVVEITLDVQRDAVSVQG
VRPVAAEAAVAAARKRYDRSKSTAAVALKGLQFVTAKVGGDGWAAVEKRFNHLQV
DGVLLRSRFGKCIGMDGSDEFAVQMFDSLARKRGIVKEVLTKAELKEFWEQLSDQGF
DNRLQTFIDMVDKNADGRITSEEVKEIIALSASANKLSKIKERADEYTALIMEELDPDNL
GYIELENLEALLLQPPSEAVASTITHSSKLSKALSMRLAPSKGMSPLHRYWQEFSFFVEE
NWKRIWVAALWLSICIALFVWKFIQYRNRAVFHIMGYCVATAKGAAETLKFNMALVL
LPVCRNTITWIRSKTKIGAVVPFNDNINFHKVIAAGVAVGVVLHAGAHLTCDFPLLLHA
SDAKYEPMKPFFGEKRPPNYWWFVKGTAGWTGVVMVVLMSISFVLAQPWFRRNKLK
PTNPLKKMTGFNAFWFTHHLFAIVYALLIVHGTSLYLTKEWYKKTVKVYNTLLMPIEN
LMIRTIDAY
>A0A287PZH2.1/75-173 Predicted protein
{ECO:0000313|EnsemblPlants:HORVU4Hr1G086500.8}
MADIEAGKPATESDTATLIPNSGSLEGSSRATKTTRFKDDDEVVEITLDVQRDAVSVQG
VRPVAAEAAVAAARKRYDRSKSTAAVALKGLQFVTAKVGGDGWAAVEKRFNHLQV
DGVLLRSRFGKCIGMDGSDEFAVQMFDSLARKRGIVKEVLTKAELKEFWEQLSDQGF
DNRLQTFIDMVDKNADGRITSEEVKEIIALSASANKLSKIKERADEYTALIMEELDPDNL
GYIELENLEALLLQPPSEAVASTITHSSKLSKALSMRLAPSKGMSPLHRYWQEFSFFVEE
NWKRIWVAALWLSICIALFVWKFIQYRNRAVFHIMGYCVATAKGAAETLKFNMALVL
LPVCRNTITWIRSKTKIGAVVPFNDNINFHKVIAAGVAVGVVLHAGAHLTCDFPLLLHA
SDAKYEPMKPFFGEKRPPNYWWFVKGTAGWTGVVMVVLMSISFVLAQPWFRRNKLK
PTNPLKKMTGFNAFWFTHHLFAIVYALLIVHGTSLYLTKEWYKKTTWMYIAYPVFLYL
CERIVRLFRSNDAVKIQKGCGISWECVGSLYDQATWFQIPKWAVHLYKLRRCISI
>A0A287PZI5.1/75-173 Predicted protein
{ECO:0000313|EnsemblPlants:HORVU4Hr1G086500.20}
MADIEAGKPATESDTATLIPNSGSLEGSSRATKTTRFKDDDEVVEITLDVQRDAVSVQG
VRPVAAEAAVAAARKRYDRSKSTAAVALKGLQFVTAKVGGDGWAAVEKRFNHLQV
DGVLLRSRFGKCIGMDGSDEFAVQMFDSLARKRGIVKEVLTKAELKEFWEQLSDQGF
DNRLQTFIDMVDKNADGRITSEEVKEIIALSASANKLSKIKERADEYTALIMEELDPDNL
GYIELENLEALLLQPPSEAVASTITHSSKLSKALSMRLAPSKGMSPLHRYWQEFSFFVEE
NWKRIWVAALWLSICIALFVWKFIQYRNRAVFHIMGYCVATAKGAAETLKFNMALVL
LPVCRNTITWIRSKTKIGAVVPFNDNINFHKVIAAGVAVGVVLHAGAHLTCDFPLLLHA
SDAKYEPMKPFFGEKRPPNYWWFVKGTAGWTGVVMVVLMSISFVLAQPWFRRNKLK
PTNPLKKMTGFNAFWFTHHLFAIVYALLIVHGTSLYLTKEWYKKTTWMYIAYPVFLYL
CERIVRLFRSNDAVKIQKVLRYILGMCWLFI

>A0A287PZM1.1/75-173 Predicted protein
{ECO:0000313|EnsemblPlants:HORVU4Hr1G086500.13}
MADIEAGKPATESDTATLIPNSGSLEGSSRATKTTRFKDDDEVVEITLDVQRDAVSVQG
VRPVAAEAAVAAARKRYDRSKSTAAVALKGLQFVTAKVGGDGWAAVEKRFNHLQV
DGVLLRSRFGKCIGMDGSDEFAVQMFDSLARKRGIVKEVLTKAELKEFWEQLSDQGF
DNRLQTFIDMVDKNADGRITSEEVKEIIALSASANKLSKIKERADEYTALIMEELDPDNL
GYIELENLEALLLQPPSEAVASTITHSSKLSKALSMRLAPSKGMSPLHRYWQEFSFFVEE
NWKRIWVAALWLSICIALFVWKFIQYRNRAVFHIMGYCVATAKGAAETLKFNMALVL
LPVCRNTITWIRSKTKIGAVVPFNDNINFHKVIAAGVAVGVVLHAGAHLTCDFPLLLHA
SDAKYEPMKPFFGEKRPPNYWWFVKGTAGWTGVVMVVLMSISFVLAQPWFRRNKLK
PTNPLKKMTGFNAFWFTHHLFAIVYALLIVHGTSLYLTKEWYKKTDMDVHRLSCLLIF
VRAHCSVV
>A0A287QPE1.1/1-45 Uncharacterized protein
{ECO:0000313|EnsemblPlants:HORVU5Hr1G024550.2}
MVGSEEFAAQMYDALARRRGIVAQVLTKDELRQFWEQLSDPGFDAKLQTFFDMVDK
NADGRITEEELKEVLTLTASANKLTKILERVDEYTALIMEELDPDQLGYIELATLESLLL
LPPSQAPTSLVAHSSNISQLISRRLVPARDANPLRRGLTATRYFLEDNWKRVWVMALW
LSINAGLFAWKFYAYRRHPTFDVMGYCVCVAKGGAETTKFNMAVILLPVCRNTVTWL
RSRTRLGAAVPFNDNINFHKVVAGGVAVGVALHGVTHLTXXFPRLLHASDEAYEPMK
RYFGQTRVPDYWWFVRGVEGVTGVIMVVLMAVAYTLAHPRFRRSKLGAGNPLKRLS
GFNMFWYTHHLFVVVYVALVVHGVCLYINRTWYKQTTWMYLAVPVLLYAGERLLR
ALRSHGLTTVRIEKVAVYPGNVIAIHMSKPHGFRYRSGQYIYVNCSEVSPFEWHPFTITS
APGDDYLSMHIRCRGDWTSRFRAIFSQICRPPSAGQSGLLRADFTSMVEHNAK
>A0A287QPE9.1/1-85 Uncharacterized protein
{ECO:0000313|EnsemblPlants:HORVU5Hr1G024550.5}
LQFLNQSVVTQGSWPEVEKRFDRLAVDGLLLRSRFGQCIGMVGSEEFAAQMYDALAR
RRGIVAQVLTKDELRQFWEQLSDPGFDAKLQTFFDMVDKNADGRITEEELKE
>A0A287QPR9.1/1-88 Uncharacterized protein
{ECO:0000313|EnsemblPlants:HORVU5Hr1G024550.4}
LRGLQFLNQSVVTQGSWPEVEKRFDRLAVDGLLLRSRFGQCIGMVGSEEFAAQMYDA
LARRRGIVAQVLTKDELRQFWEQLSDPGFDAKLQTFFDMVDKNADGRITEEELKEVLT
LTASANKLTKILERVDEYTALIMEELDPDQLGYIELATLESLLLLPPSQAPTSLVAHSSNI
SQLISRRLVPARDANPLRRGLTATRYFLEDNWKRVWVMALWLSINAGLFAWKFYAYR
RHPTFDVMGYCVCVAKGGAETTKFNMAVILLPVCRNTVTWLRSRTRLGAAVPFNDNI
NFHKVVAGGVAVGVALHGVTHLTXXX
>A0A287QQ22.1/98-193 Uncharacterized protein
{ECO:0000313|EnsemblPlants:HORVU5Hr1G024550.3}
MERQAGNGNAIAGPPPEENKGPDGKSASRRSTRFKEENAYVEVTLDVRADDAVAVQS
VRACLLYTSPSPRDXXXXXXXXXXXXXXXXXXXXXXXXXXXXXXXXXALRGLQFL
NQSVVTQGSWPEVEKRFDRLAVDGLLLRSRFGQCIGMVGSEEFAAQMYDALARRRGI
VAQVLTKDELRQFWEQLSDPGFDAKLQTFFDMVDKNADGRITEEELKEVLTLTASAN
KLTKILERVDEYTALIMEELDPDQLGYIELATLESLLLLPPSQAPTSLVAHSSNISQLISRR
LVPARDANPLRRGLTATRYFLEDNWKRVWVMALWLSINAGLFAWKFYAYRRHPTFD
VMGYCVCVAKGGAETTKFNMAVILLPVCRNTVTWLRSRTRLGAAVPFNDNINFHKVV
AGGVAVGVALHGVTHLTXXFPRLLHASDEAYEPMKRYFGQTRVPDYWWFVRGVEG
VTGVIMVVLMAVAYTLAHPRFRRSKLGAGNPLKRLSGFNMFWYTHHLFVVVYVALV

VHGVCLYINRTWYKQTTWMYLAVPVLLYAGERLLRALRSHGLTTVRIEKVAVYPGN
VIAIHMSKPHGFRYRSGQYIYVNCSEVSPFEWHPFTITSAPGDDYLSMHIRCRGDWTSR
FRAIFSQICRPPSAGQSGLLRADFTSMVEHNAKFPRLLIDGPYGAPAQDYRKYDVLLLIG
LGIGATPLISIVKDVLNNVHRREEHAGDEGFMTKRVYFYWCTREEGSFEWFRGVMNE
VAERDAAGEESVVELHNHCTSVYEEGDARSAMVVMLQALHHAKSGVXXXXXXXXX
XXXXXXXXXXXXXXXXXXXXXXXXXXXXXDQKLTPELRRLSHDFSHRTTTKFVFH
KENF
>A0A287RH49.1/164-230 Uncharacterized protein
{ECO:0000313|EnsemblPlants:HORVU5Hr1G062490.2}
MWTPSRGSNARRAGHRRIAEGLPDDQTTNTDTSDNESFTTAYGDEFFAAAAGGSGAG
GMLPAFLADQEDLVEVMLELDEESMVVRSVTPTRAALYSAAAMPHTPEAPGGALSRC
SSTSSRIRKKFAWLRSPSPAPSPSPRVPTPAELQREAAMAARERRRIQARVNRSRAGAK
RALKGLRFISRTTGSLEAAELWRRVEERFNALAHDGLLSRDNFGECIGTPSHTPTLFLSR
QQPPSVLLPSHPIV
>A0A287RH54.1/164-266 Uncharacterized protein
{ECO:0000313|EnsemblPlants:HORVU5Hr1G062490.7}
MWTPSRGSNARRAGHRRIAEGLPDDQTTNTDTSDNESFTTAYGDEFFAAAAGGSGAG
GMLPAFLADQEDLVEVMLELDEESMVVRSVTPTRAALYSAAAMPHTPEAPGGALSRC
SSTSSRIRKKFAWLRSPSPAPSPSPRVPTPAELQREAAMAARERRRIQARVNRSRAGAK
RALKGLRFISRTTGSLEAAELWRRVEERFNALAHDGLLSRDNFGECIGMVDSKEFAEGI
FDALARRRKQNLERINKEELYDFWLQISDQSFDARLQIFFDMVDTNVDGRITREEVQEL
IVLSASANKLAKLKEQAEEYASLIMEELDPENLGYIEVLWQLETLLLQRDTYMNYSRPL
STASGAQWSQNLGVGGGGAAAGGGGTAARVGPRREEGGVARAGGGGGELAARLGG
GAVGGGHGGALRVEVRAVPADGGVPGDGVLPADGQGRRRDAQAQHGPRPPPRLPQH
PHLAPLLLGPLLRPLRRQHHLPQDDRDGDRGGDHVARGEPSGVRLPAGDRVGAGGVP
AGGGRVRGHQADVRGAHLRGGGGDGHRHGGAHDRLLHPRHPPLPQGREGRLRLPPP
AAPQPPRRLQRVLVLPPPPRLRLPAPPPPRLLPLPRPPLVRENDMDVHLCPSGALCWRE
DAAGLAVERPPCPNPQGVASTWKCTDNKNVKALRISIQEWTIYLSSVSDYLSI
>A0A287RHB9.1/1-45 Uncharacterized protein
{ECO:0000313|EnsemblPlants:HORVU5Hr1G062490.9}
MVDSKEFAEGIFDALARRRKQNLERINKEELYDFWLQISDQSFDARLQIFFDMVDTNV
DGRITREEVQELIVLSASANKLAKLKEQAEEYASLIMEELDPENLGYIEVLWQLETLLL
QRDTYMNYSRPLSTASGAQWSQNLGVGGGGAAAGGGGSKGEEDPQTTWGGGMRER
RRGWGRGVKKAASHVRVAAEENWRRAWVVALWVAAMAALFVWKFVQYRRTAGF
QVMGYCLPTAKGAAETLKLNMALVLLPVCRNTLTWLRSSWARFFVPFDDNITFHKMI
ATAIVVGITLHAGNHLACDFPRVIASGPEEYRLVAGAFGATKPTYAGLISGVEGVTGIA
MVVLMTVSFTLATHPFRKGEKAASASRLPPPLNRLAGFNAFWYSHHLLAFVYLLLLLH
GYFLFLVRRWYEKTTWMYISVPLVLYVGERMLRALRSNAHPVQILKCPIISPFEWHPFS
ITSAPGDDYLTVHIRTNGDWTQELKRIFVENYFPPHLNRRTSFSELGAAEPRTSPPPKLL
VDGPYGAPAQDFRNYDVLLLVGLGIGATPFISILKDLLNNIKLADELMDLAMETTQTSR
SDDSANSFSVSTASSNRKRSYRTSRAHFYWVTREPMSFEWFKGVMDEVAEMDKKGVI
ELHNYLTSVYEERDARTTLLSMVQALNHAKHGVDIVSGTRVRTHFARPNWKEVFTKI
AAKQPNSTVGVFYCGAPTLAIELKNLSHEMSHKTSTRFHFHKEYF
>A0A287RHI3.1/164-266 Uncharacterized protein
{ECO:0000313|EnsemblPlants:HORVU5Hr1G062490.3}

MWTPSRGSNARRAGHRRIAEGLPDDQTTNTDTSDNESFTTAYGDEFFAAAAGGSGAG
GMLPAFLADQEDLVEVMLELDEESMVVRSVTPTRAALYSAAAMPHTPEAPGGALSRC
SSTSSRIRKKFAWLRSPSPAPSPSPRVPTPAELQREAAMAARERRRIQARVNRSRAGAK
RALKGLRFISRTTGSLEAAELWRRVEERFNALAHDGLLSRDNFGECIGMVDSKEFAEGI
FDALARRRKQNLERINKEELYDFWLQISDQSFDARLQIFFDMVDTNVDGRITREEVQEL
IVLSASANKLAKLKEQAEEYASLIMEELDPENLGYIELWQLETLLLQRDTYMNYSRPLS
TASGAQWSQNLGVGGGGAAAGGGGSKGEEDPQTTWGGGMRERRRGWGRGVKKAA
SHVRVAAEENWRRAWVVALWVAAMAALFVWKFVQYRRTAGFQVMGYCLPTAKGA
AETLKLNMALVLLPVCRNTLTWLRSSWARFFVPFDDNITFHKMIATAIVVGITLHAGN
HLACDFPRVIASGPEEYRLVAGAFGATKPTYAGLISGVEGVTGIAMVVLMTVSFTLAT
HPFRKGEKAASASRLPPPLNRLAGFNAFWYSHHLLAFVYLLLLLHGYFLFLVRRWYEK
TTWMYISVPLVLYVGERMLRALRSNAHPVQILKVCCFYLEVY
>A0A287RHI8.1/164-266 Uncharacterized protein
{ECO:0000313|EnsemblPlants:HORVU5Hr1G062490.8}
MWTPSRGSNARRAGHRRIAEGLPDDQTTNTDTSDNESFTTAYGDEFFAAAAGGSGAG
GMLPAFLADQEDLVEVMLELDEESMVVRSVTPTRAALYSAAAMPHTPEAPGGALSRC
SSTSSRIRKKFAWLRSPSPAPSPSPRVPTPAELQREAAMAARERRRIQARVNRSRAGAK
RALKGLRFISRTTGSLEAAELWRRVEERFNALAHDGLLSRDNFGECIGMVDSKEFAEGI
FDALARRRKQNLERINKEELYDFWLQISDQSFDARLQIFFDMVDTNVDGRITREEVQEL
IVLSASANKLAKLKEQAEEYASLIMEELDPENLGYIEVLWQLETLLLQRDTYMNYSRPL
STASGAQWSQNLGVGGGGAAAGGGGSKGEEDPQTTWGGGMRERRRGWGRGVKKA
ASHVRVAAEENWRRAWVVALWVAAMAALFVWKFVQYRRTAGFQVMGYCLPTAKG
AAETLKLNMALVLLPVCRNTLTWLRSSWARFFVPFDDNITFHKMIATAIVVGITLHAG
NHLACDFPRVIASGPEEYRLVAGAFGATKPTYAGLISGVEGVTGIAMVVLMTVSFTLA
THPFRKGEKAASASRLPPPLNRLAGFNAFWYSHHLLAFVYLLLLLHGYFLFLVRRWYE
KTTWMYISVPLVLYVGERMLRALRSNAHPVQILKVLLLPGSVLTIKMSKPYGFRYRSG
QYIFLQCPIISPFEWHPFSITSAPGDDYLTVHIRTNGDWTQELKRIFVENYFPPHLNRRTS
FSELGAAEPRTSPPPKLLVDGPYGAPAQDFRNYDVLLLVGLGIGATPFISILKDLLNNIK
LADELMDLAMETTQTSRSDDSANSFSVSTASSNRKRSYRTSRAHFYWVTREPMSFEWF
KGVMDEVAEMDKKGVIELHNYLTSVYEERDARTTLLSMVQALNHAKHGVDIVSGTR
VRTHFARPNWKEVFTKIAAKQPNSTVGVFYCGAPTLAIELKNLSHEMSHKTSTRFHFH
KEYF
>A0A287RHL4.1/164-266 Uncharacterized protein
{ECO:0000313|EnsemblPlants:HORVU5Hr1G062490.6}
MWTPSRGSNARRAGHRRIAEGLPDDQTTNTDTSDNESFTTAYGDEFFAAAAGGSGAG
GMLPAFLADQEDLVEVMLELDEESMVVRSVTPTRAALYSAAAMPHTPEAPGGALSRC
SSTSSRIRKKFAWLRSPSPAPSPSPRVPTPAELQREAAMAARERRRIQARVNRSRAGAK
RALKGLRFISRTTGSLEAAELWRRVEERFNALAHDGLLSRDNFGECIGMVDSKEFAEGI
FDALARRRKQNLERINKEELYDFWLQISDQSFDARLQIFFDMVDTNVDGRITREEVQEL
IVLSASANKLAKLKEQAEEYASLIMEELDPENLGYIEVLWQLETLLLQRDTYMNYSRPL
STASGAQWSQNLGVGGGGAAAGGGGSKGEEDPQTTWGGGMRERRRGWGRGVKKA
ASHVRVAAEENWRRAWVVALWVAAMAALFVWKFVQYRRTAGFQVMGYCLPTAKG
AAETLKLNMALVLLPVCRNTLTWLRSSWARFFVPFDDNITFHKMIATAIVVGITLHAG
NHLACDFPRVIASGPEEYRLVAGAFGATKPTYAGLISGVEGVTGIAMVVLMTVSFTLA
THPFRKGEKAASASRLPPPLNRLAGFNAFWYSHHLLAFVYLLLLLHGYFLFLVRRWYE
KTTWMYISVPLVLYVGERMLRALRSNAHPVQILKVLLLPGSVLTIKMSKPYGFRYRSG

QYIFLQCPIISPFEWHPFSITSAPGDDYLTVHIRTNGDWTQELKRIFVENYFPPHLNRRTS
FSELGAAEPRTSPPPKLLVDGPYGAPAQDFRNYDVLLLVGLGIGATPFISILKDLLNNIK
LADELMDLAMETTQTSRSDDSANSFSVSTASSNRKRSYRTSRAHFYWVTREPMSFEWF
KGVMDEVAEMDKKGVIELHNYLTSVYEERDARTTLLSMVQALNHAKHGVDIVSGTR
VRTHFARPNWKEVFTKIAAKQPNSTVGMVFRSNR
>A0A287RXM8.1/1-44 Uncharacterized protein
{ECO:0000313|EnsemblPlants:HORVU5Hr1G078630.2}
MKENAFAGELFDALARRRDISGDSISKAELLEFWDQISDTSFDSRLQTFFDMVDKDAD
GRITEAEVGEIIRLSAAANDLKKITERIEEYARLIMEELDPDNLGYIELYNLETLLLQAPP
TQPSRGGTTSSRNLSQMLSQHLKPTTEPNPLRRWYRRASYFLEDNWRRCWVIILWFSIC
AGLFAWKFVQYRRRAVFEVMGYCVCVAKGGAETLKFNMALVLLPVCRNTITWLRNR
TAAGRVVPFDDNLNFHKVIAAGITVGAGMHIISHLACDFPRLLHATEEEYEPMKPFFGD
VQPPNYWWFVKGTEGWTGLVMLALMAVAFTLATPWFRRGRVRLPGPLSRLTGFNAF
WYTHHLFIIVYALLIVHGHFLYLTKKWQKKSTWMYVAAPMVLYACERLARALRSSVR
PVKILKVAVYPGNVLSLRFSKPQGFRCKSGQYIFVNCAAVSPFQWHPFSITSAPHDDYIS
VHIRTLGDWTRELKSVFSKVCRPPTDGKSERAAPRGVRGRRRRHAQPEQLPNGADRR
AVRRAGAGLQAVRRGAAGGAGHRGHAHDLHHQGHHQQHEAARRRHRVRQPQRQER
VGGVVPDPAGLLLLGDAGGRLLRLVPRRHGRGGRERQEGHHRAPQLLHQRVRGRGRP
VGAHRHAPVPQPRQARRRRRRLRHPREDALRPPELAQGVQGHRPEARRAASRSVLLRR
AGADQGAAPACPRFLEEDEHQIRVPQGEF
>A0A287RXQ7.1/158-253 Uncharacterized protein
{ECO:0000313|EnsemblPlants:HORVU5Hr1G078630.1}
MHNYGGGGGGGGGAGDIVEAGGERVVPHSGPLVGKRSATRKXXXKSARFADSVSAP
LRAGAGGRGNHHQDHDDDDDYVEVTLDVRDDSVAVHSVKPAAGGEEDPDVTLLAR
ALETRSSSYGGRAGGHGGGVLRNASTRIKQVSQELRRIASVKRRPSRIDRSKSAAAHAL
KGLKFISKPDGWPAVEKRFDELAENGGLLHRSKFGKCIGMKENAFAGELFDALARRRD
ISGDSISKAELLEFWDQISDTSFDSRLQTFFDMVDKDADGRITEAEVGEIIRLSAAANDL
KKITERIEEYARLIMEELDPDNLGYIELYNLETLLLQAPPTQPSRGGTTSSRNLSQMLSQ
HLKPTTEPNPLRRWYRRASYFLEDNWRRCWVIILWFSICAGLFAWKFVQYRRRAVFEV
MGYCVCVAKGGAETLKFNMALVLLPVCRNTITWLRNRTAAGRVVPFDDNLNFHKVI
AAGITVGAGMHIISHLACDFPRLLHATEEEYEPMKPFFGDVQPPNYWWFVKGTEGWT
GLVMLALMAVAFTLATPWFRRGRVRLPGPLSRLTGFNAFWYTHHLFIIVYALLIVHGH
FLYLTKKWQKKSTWMYVAAPMVLYACERLARALRSSVRPVKILKVAVYPGNVLSLR
FSKPQGFRCKSGQYIFVNCAAVSPFQWHPFSITSAPHDDYISVHIRTLGDWTRELKSVFS
KVCRPPTDGKSERAAPRGVRGRRRRHAQPEQLPNGADRRAVRRAGAGLQAVRRGAA
GGAGHRGHAHDLHHQGHHQQHEAARRRHRVRQPQRQERVGGVVPDPAGLLLLGDA
GGRLLRLVPRRHGRGGRERQEGHHRAPQLLHQRVRGRGRPVGAHRHAPVPQPRQAR
RRRRLRHPREDALRPPELAQGVQGHRPEARRAASRSVLLRRAGADQGAAPACPRFLEE
DEHQIRVPQGEFLIFQAGASLVTRSFSLFVTRRRSSSFCEEKLCLIY
>A0A287TX50.1/155-257 Uncharacterized protein
{ECO:0000313|EnsemblPlants:HORVU6Hr1G035970.1}
MWTPSRGPGSGRRAGLRRIADYIGDDHTDASDNESFITSHSDELLSSTSAAAGAGGSVG
MLPAFLADQSDLVEVMLELDEESMVVRSVTPTAGPAAPAGAGTHTPGSGRNLSRSSST
SSKIRRTFAWLRSPAASAPEQPREAAMASRERRRVQARLDRSLSGARRALKGLRFISRA
TGSAEATALWGAVEERFDALSRDGLLARDDFGDCIGMVDSKEFAVGIFDALARRRRQ
TLQRVTKEELHDFWLQISDQSFDARLQIFFDMYVQYRTIMLFPCFLRSAQVAHAFLAAI

AKNKFQGGHQRRREDHEGGSAGADRSERVGEQALEAQGAGRGVRAAHHGGARPGG
PRLHRAVAAGGAAPATRRLHELQPAAQQRQRQRGAVEPGHRRAGEAGDGGGAGTGR
VAVEPAARGGEGARGGGGELAARVGARAVGRGDGGAVRVEVRAVPAVGGVRGDGA
LPADGQGRRRDAQAQHGAGPPPRLPHHAHAPPLLVGALRRALGRLHRLPQGGGHGDR
GGDLPARREPSGVRLPAADRVEPGGVPAADRILRGGEADIQEPAVRGGGRDRGGDGG
AHGRLLHPGRPAVAEGVHPPDPAAVPAGPPRRVQRLLVLPPPARRRLPPAARARLVHV
PRQQVVPEDDMDVHSRALCSTRR
>A0A287TXB8.1/22-90 Uncharacterized protein
{ECO:0000313|EnsemblPlants:HORVU6Hr1G035970.6}
LARDDFGDCIGTPCSVHACHFGSTRFTARSRAGIGDELFDGRFAGMVDSKEFAVGIFDA
LARRRRQTLQRVTKEELHDFWLQISDQSFDARLQIFFDMVDTNVDGRITREEVQELIVL
SASANKLSRLKEQAEEYALLIMEELDPEGLGYIELWQLEALLLQRDAYMSYSRPLSSGS
GSAAQWSQDIGAQEKPATAAAPGRGGWRWSPRRAAGRARVAAEENWRRAWVLAL
WVAAMAALFAWRFAQYRRSVAFEGMGHCLPTAKGAAETLKLNMALVLLPVCRITLT
RLRSSWARFVVPLDDCIAFHKVVAMAIAAGICLHAGNHLACDFPRLIASSPAEYRPLTG
FFGEEKPTYRSLLSGVVGVTGVVMVALMAVSFTLAARPLRRASTRRTRLPSPLGHLAG
FNAFWYSHHLLVVVYLLLLVHGWFMFLVSKWYQRTTWMYIAVPFALHVGERTLRAL
RSKAYAAKILKVCLLPGNVLTITMSKPYGFRYRSGQYVFLQCPTISPFEWHPFSITSAPG
DDYISVHIQTRGDWTQELKHIFVENYMSPCLPGRASFGDLGMAEQKSPPRLLVDGPYG
APAQDFRNYDVLLLVGLGIGATPFISILRDLLNNIKLADELMDLSMETSRSEDSSNTTFS
VSTASSNKRRAYRTSRAHFYWVTREPGSFEWFKSVMDEVAEMDKKGVIELHNYLTSV
YEERDARTTLLSMVQALNHAKNGIDIVSGTRVRTHFARPNWKEEFTRISAKHPGSTVG
VFYCGKPTLAKELKKLSLEMSHKTTTRFHFHKEYF
>A0A287TXD0.1/224-291 Uncharacterized protein
{ECO:0000313|EnsemblPlants:HORVU6Hr1G035970.2}
MWTPSRGPGSGRRAGLRRIADYIGDDHTDASDNESFITSHSDELLSSTSAAAGAGGSVG
MLPAFLADQSDLVEVMLELDEESMVVRSVTPTAGPAAPAGAGTHTPGSGRNLSRSSST
SSKIRRTFAWLRSPAASAPEQPREAAMASRERRRVQARLDRSLSGARRALKGLRFISRA
TGSAEATALWGAVEERFDALSRDGLLARDDFGDCIGTPCSVHACHFGSTRFTARSRAG
IGDELFDGRFAGMVDSKEFAVGIFDALARRRRQTLQRVTKEELHDFWLQISDQSFDAR
LQIFFDMVDTNVDGRITREEVQELIVLSASANKLSRLKEQAEEYALLIMEELDPEGLGYI
ELWQLEALLLQRDAYMSYSRPLSSGSGSAAQWSQDIGAQEKPATAAAPGRGGWRWS
PRRAAGRARVAAEENWRRAWVLALWVAAMAALFAWRFAQYRRSVAFEGMGHCLP
TAKGAAETLKLNMALVLLPVCRITLTRLRSSWARFVVPLDDCIAFHKVVAMAIAAGIC
LHAGNHLACDFPRLIASSPAEYRPLTGFFGEEKPTYRSLLSGVVGVTGVVMVALMAVS
FTLAARPLRRASTRRTRLPSPLGHLAGFNAFWYSHHLLVVVYLLLLVHGWFMFLVSK
WYQRTTWMYIAVPFALHVGERTLRALRSKAYAAKILKCPTISPFEWHPFSITSAPGDDY
ISVHIQTRGDWTQELKHIFVENYMSPCLPGRASFGDLGMAEQKSPPRLLVDGPYGAPA
QDFRNYDVLLLVGLGIGATPFISILRDLLNNIKLADELMDLSMETSRSEDSSNTTFSVST
ASSNKRRAYRTSRAHFYWVTREPGSFEWFKSVMDEVAEMDKKGVIELHNYLTSVYEE
RDARTTLLSMVQALNHAKNGIDIVSGTRVRTHFARPNWKEEFTRISAKHPGSTVGVFY
CGKPTLAKELKKLSLEMSHKTTTRFHFHKEYF
>A0A287TXD0.1/155-234 Uncharacterized protein
{ECO:0000313|EnsemblPlants:HORVU6Hr1G035970.2}
MWTPSRGPGSGRRAGLRRIADYIGDDHTDASDNESFITSHSDELLSSTSAAAGAGGSVG
MLPAFLADQSDLVEVMLELDEESMVVRSVTPTAGPAAPAGAGTHTPGSGRNLSRSSST

SSKIRRTFAWLRSPAASAPEQPREAAMASRERRRVQARLDRSLSGARRALKGLRFISRA
TGSAEATALWGAVEERFDALSRDGLLARDDFGDCIGTPCSVHACHFGSTRFTARSRAG
IGDELFDGRFAGMVDSKEFAVGIFDALARRRRQTLQRVTKEELHDFWLQISDQSFDAR
LQIFFDMVDTNVDGRITREEVQELIVLSASANKLSRLKEQAEEYALLIMEELDPEGLGYI
ELWQLEALLLQRDAYMSYSRPLSSGSGSAAQWSQDIGAQEKPATAAAPGRGGWRWS
PRRAAGRARVAAEENWRRAWVLALWVAAMAALFAWRFAQYRRSVAFEGMGHCLP
TAKGAAETLKLNMALVLLPVCRITLTRLRSSWARFVVPLDDCIAFHKVVAMAIAAGIC
LHAGNHLACDFPRLIASSPAEYRPLTGFFGEEKPTYRSLLSGVVGVTGVVMVALMAVS
FTLAARPLRRASTRRTRLPSPLGHLAGFNAFWYSHHLLVVVYLLLLVHGWFMFLVSK
WYQRTTWMYIAVPFALHVGERTLRALRSKAYAAKILKCPTISPFEWHPFSITSAPGDDY
ISVHIQTRGDWTQELKHIFVENYMSPCLPGRASFGDLGMAEQKSPPRLLVDGPYGAPA
QDFRNYDVLLLVGLGIGATPFISILRDLLNNIKLADELMDLSMETSRSEDSSNTTFSVST
ASSNKRRAYRTSRAHFYWVTREPGSFEWFKSVMDEVAEMDKKGVIELHNYLTSVYEE
RDARTTLLSMVQALNHAKNGIDIVSGTRVRTHFARPNWKEEFTRISAKHPGSTVGVFY
CGKPTLAKELKKLSLEMSHKTTTRFHFHKEYF
>A0A287TXE1.1/155-233 Uncharacterized protein
{ECO:0000313|EnsemblPlants:HORVU6Hr1G035970.3}
MWTPSRGPGSGRRAGLRRIADYIGDDHTDASDNESFITSHSDELLSSTSAAAGAGGSVG
MLPAFLADQSDLVEVMLELDEESMVVRSVTPTAGPAAPAGAGTHTPGSGRNLSRSSST
SSKIRRTFAWLRSPAASAPEQPREAAMASRERRRVQARLDRSLSGARRALKGLRFISRA
TGSAEATALWGAVEERFDALSRDGLLARDDFGDCIGTPCSVHACHFGSTRFTARSRAG
IGDELFDGRFAGMVDSKEFAVGIFDALARRRRQTLQRVTKEELHDFWLIVLSASANKL
SRLKEQAEEYALLIMEELDPEGLGYIELWQLEALLLQRDAYMSYSRPLSSGSGSAAQW
SQDIGAQEKPATAAAPGRGGWRWSPRRAAGRARVAAEENWRRAWVLALWVAAMA
ALFAWRFAQYRRSVAFEGMGHCLPTAKGAAETLKLNMALVLLPVCRITLTRLRSSWA
RFVVPLDDCIAFHKVVAMAIAAGICLHAGNHLACDFPRLIASSPAEYRPLTGFFGEEKP
TYRSLLSGVVGVTGVVMVALMAVSFTLAARPLRRASTRRTRLPSPLGHLAGFNAFWY
SHHLLVVVYLLLLVHGWFMFLVSKWYQRTLEEREEKRKGKRALS
>A0A287TXE1.1/223-290 Uncharacterized protein
{ECO:0000313|EnsemblPlants:HORVU6Hr1G035970.3}
MWTPSRGPGSGRRAGLRRIADYIGDDHTDASDNESFITSHSDELLSSTSAAAGAGGSVG
MLPAFLADQSDLVEVMLELDEESMVVRSVTPTAGPAAPAGAGTHTPGSGRNLSRSSST
SSKIRRTFAWLRSPAASAPEQPREAAMASRERRRVQARLDRSLSGARRALKGLRFISRA
TGSAEATALWGAVEERFDALSRDGLLARDDFGDCIGTPCSVHACHFGSTRFTARSRAG
IGDELFDGRFAGMVDSKEFAVGIFDALARRRRQTLQRVTKEELHDFWLIVLSASANKL
SRLKEQAEEYALLIMEELDPEGLGYIELWQLEALLLQRDAYMSYSRPLSSGSGSAAQW
SQDIGAQEKPATAAAPGRGGWRWSPRRAAGRARVAAEENWRRAWVLALWVAAMA
ALFAWRFAQYRRSVAFEGMGHCLPTAKGAAETLKLNMALVLLPVCRITLTRLRSSWA
RFVVPLDDCIAFHKVVAMAIAAGICLHAGNHLACDFPRLIASSPAEYRPLTGFFGEEKP
TYRSLLSGVVGVTGVVMVALMAVSFTLAARPLRRASTRRTRLPSPLGHLAGFNAFWY
SHHLLVVVYLLLLVHGWFMFLVSKWYQRTLEEREEKRKGKRALS
>A0A287TY09.1/155-257 Uncharacterized protein
{ECO:0000313|EnsemblPlants:HORVU6Hr1G035970.5}
MWTPSRGPGSGRRAGLRRIADYIGDDHTDASDNESFITSHSDELLSSTSAAAGAGGSVG
MLPAFLADQSDLVEVMLELDEESMVVRSVTPTAGPAAPAGAGTHTPGSGRNLSRSSST
SSKIRRTFAWLRSPAASAPEQPREAAMASRERRRVQARLDRSLSGARRALKGLRFISRA

TGSAEATALWGAVEERFDALSRDGLLARDDFGDCIGMVDSKEFAVGIFDALARRRRQ
TLQRVTKEELHDFWLQISDQSFDARLQIFFDMVDTNVDGRITREEVQELIVLSASANKL
SRLKEQAEEYALLIMEELDPEGLGYIELWQLEALLLQRDAYMSYSRPLSSGSGSAAQW
SQDIGAQEKPATAAAPGRGGWRWSPRRAAGRARVAAEENWRRAWVLALWVAAMA
ALFAWRFAQYRRSVAFEGMGHCLPTAKGAAETLKLNMALVLLPVCRITLTRLRSSWA
RFVVPLDDCIAFHKVVAMAIAAGICLHAGNHLACDFPRLIASSPAEYRPLTGFFGEEKP
TYRSLLSGVVGVTGVVMVALMAVSFTLAARPLRRASTRRTRLPSPLGHLAGFNAFWY
SHHLLVVVYLLLLVHGWFMFLVSKWYQRTTWMYIAVPFALHVGERTLRALRSKAYA
AKILKVCLLPGNVLTITMSKPYGFRYRSGQYVFLQCPTISPFEWHPFSITSAPGDDYISV
HIQTRGDWTQELKHIFVENYMSPCLPGRASFGDLGMAEQKSPPRLLVDGPYGAPAQDF
RNYDVLLLVGLGIGATPFISILRDLLNNIKLADELMDLSMETSRSEDSSNTTFSVSTASS
NKRRAYRTSRAHFYWVTREPGSFEWFKSVMDEVAEMDKKGVIELHNYLTSVYEERD
ARTTLLSMVQALNHAKNGIDIVSGTRVRTHFARPNWKEEFTRISAKHPGSTVGVFYCG
KPTLAKELKKLSLEMSHKTTTRFHFHKEYF
>B2D0N5.1/75-173 Respiratory burst oxidase-like protein B1
{ECO:0000313|EMBL:ACB56481.1}
MADIEAGKPATESDTATLIPNSGSLEGSSRATKTTRFKDDDEVVEITLDVQRDAVSVQG
VRPVAAEAAVAAARKRYDRSKSTAAVALKGLQFVTAKVGGDGWAAVEKRFNHLQV
DGVLLRSRFGKCIGMDGSDEFAVQMFDSLARKRGIVKEVLTKAELKEFWEQLSDQGF
DNRLQTFIDMVDKNADGRITSEEVKEIIALSASANKLSKIKERADEYTALIMEELDPDNL
GYIELENLEALLLQPPSEAVASTITHSSKLSKALSMRLAPSKGMSPLHRYWQEFSFFVEE
NWKRIWVAALWLSICIALFVWKFIQYRNRAVFHIMGYCVATAKGAAETLKFNMALVL
LPVCRNTITWIRSKTKIGAVVPFNDNINFHKVIAAGVAVGVVLHAGAHLTCDFPLLLHA
SDAKYEPMKPFFGEKRPPNYWWFVKGTAGWTGVVMVVLMSISFVLAQPWFRRNKLK
PTNPLKKMTGFNAFWFTHHLFAIVYALLIVHGTSLYLTKEWYKKTTWMYIAYPVFLYL
CERIVRLFRSNDAVKIQKVAVYPGNVLALYMTKPPGFRYRSGQYIFINCGAVSPYEWH
PFSVTSAPGDNYLSVHIRTRGDWTSRLRTVFSEACRPPTEGESGLLRADLSVGITDSNAR
FPKLMIDGPYGAPAQDYREYDVLLLIGLGIGATPLISIVKDVLNHIQRGESVGGTEPDGS
GKAKKKPFMTKRAYSYWVTREEGSFEWFRGVMNEVAEKDKDGVIELHNHCSSVYQE
GDARSALIVMLQELNHAKKGVDILSGTSVKTHFARPNWRSVFKRIAVNHENQRVGVF
YCGEPVLVAQLRQLSADFTHNTNTKFEFHKENF
>F2D3P5.1/164-266 Predicted protein {ECO:0000313|EMBL:BAJ89716.1}=
HORVU5Hr1G062490.5
MWTPSRGSNARRAGHRRIAEGLPDDQTTNTDTSDNESFTTAYGDEFFAAAAGGSGAG
GMLPAFLADQEDLVEVMLELDEESMVVRSVTPTRAALYSAAAMPHTPEAPGGALSRC
SSTSSRIRKKFAWLRSPSPAPSPSPRVPTPAELQREAAMAARERRRIQARVNRSRAGAK
RALKGLRFISRTTGSLEAAELWRRVEERFNALAHDGLLSRDNFGECIGMVDSKEFAEGI
FDALARRRKQNLERINKEELYDFWLQISDQSFDARLQIFFDMVDTNVDGRITREEVQEL
IVLSASANKLAKLKEQAEEYASLIMEELDPENLGYIELWQLETLLLQRDTYMNYSRPLS
TASGAQWSQNLGVGGGGAAAGGGGSKGEEDPQTTWGGGMRERRRGWGRGVKKAA
SHVRVAAEENWRRAWVVALWVAAMAALFVWKFVQYRRTAGFQVMGYCLPTAKGA
AETLKLNMALVLLPVCRNTLTWLRSSWARFFVPFDDNITFHKMIATAIVVGITLHAGN
HLACDFPRVIASGPEEYRLVAGAFGATKPTYAGLISGVEGVTGIAMVVLMTVSFTLAT
HPFRKGEKAASASRLPPPLNRLAGFNAFWYSHHLLAFVYLLLLLHGYFLFLVRRWYEK
TTWMYISVPLVLYVGERMLRALRSNAHPVQILKVLLLPGSVLTIKMSKPYGFRYRSGQ
YIFLQCPIISPFEWHPFSITSAPGDDYLTVHIRTNGDWTQELKRIFVENYFPPHLNRRTSFS

ELGAAEPRTSPPPKLLVDGPYGAPAQDFRNYDVLLLVGLGIGATPFISILKDLLNNIKLA
DELMDLAMETTQTSRSDDSANSFSVSTASSNRKRSYRTSRAHFYWVTREPMSFEWFK
GVMDEVAEMDKKGVIELHNYLTSVYEERDARTTLLSMVQALNHAKHGVDIVSGTRV
RTHFARPNWKEVFTKIAAKQPNSTVGVFYCGAPTLAIELKNLSHEMSHKTSTRFHFHK
EYF
>F2DKD4.1/158-256 Predicted protein {ECO:0000313|EMBL:BAJ95555.1,
ECO:0000313|EnsemblPlants:HORVU3Hr1G069780.1}
MRGGASAGQPRWGSAGTTPRSLSTGSSPRGSEPCSEDGEELVEVTLDLQDDDTIVLRSV
EPAAASASGPGAPRPPADGASSSSTSSRSPSMRRTSSYRLLQLSQELMAGARHLSHDLT
KRFSRSHSHSRDDAHHHQHQPPSGIESALAARAARRQRAQLDRTRSGAHRALRGLRFI
SSNKASNAWREVQANFDRLARDGHLSRSDFAECIGMTESKEFALELFDTLSRRRQMKL
DTISKEELREIWQQITDNSFDSRLQIFFDMVDKNADGRIGEAEVKEIIMLSASANKLSRL
KEQAEEYAALIMEELDPEELGYIELWQLETLLLQKDTYVNYSQALSYTSQALSQNLAL
RKRGSIRKIGNSLIYYLEDNWKRLWVLALWIGIMAGLFTWKFIQYRERYVFSVMGYCV
TTAKGAAETLKLNMAIILLPVCRNTITWLRNTRAARVLPFDDNINFHKTIAAAIVVGVIL
HAGNHLVCDFPRLIRSSEETYAPLGIYFGETKPTYLALIKGVEGITGIIMVVCMIIAFTLA
TRWFRRSLVKLPKPFDKLTGFNAFWYSHHLFAIVYVALIVHGQCVYLIRVWYRKSTW
MYLAVPVCLYLGERILRFFRSGSYAVRLLKVAIYPGNVLTLQMTKPATFRYKSGQYMF
VQCPAVSPFEWHPFSITSAPGDEYLSIHVRQLGDWTRELKRVFSAACEPPVSGKSGLLR
ADETTKKTLPKLLIDGPYGSPAQDYGKYDVLLLVGLGIGATPFISILKDLLNNIIKMEEE
EDTSTDLYPPVGRNKPHVDLGTLMRVTTRPKKVLKTTNAYFYWVTREQGSFDWFKG
VMNEIAEMDQRNIIEMHNYLTSVYEEGDARSALITMLQALNHAKNGVDVVSGTKVRT
HFARPNWKKVLAKIASKHPYAKIGVFYCGAPVLAQELAKLCHEFNGKCTTKFEFHKE
YF
>F2DNI1.1/71-172 Predicted protein {ECO:0000313|EMBL:BAJ96652.1}
HORVU3Hr1G087210.8
MADRPAPPLDGITVDGGGRTPPAGPGLPRPPGFRGLMQQPSRLASGVRQFASRVSMKV
PEVVPGIRPGGGRMTRMQSSAQMGLKGLRFLDKTSGSKEGWKAVERRFDEMSKASGR
LPKESFGKCIGMGDSKEFAGELFVTLSRRRSIEPEQGITKEQLREFWTEMTDQNFDSRLR
IFFDMCDKNGDGMLTEDEVKEVIILSASANKLAKLKSHAATYSSLIMEELDPDDRGYIE
IWQLETLLRGMVSAQAPEVKLKRTTSSLARTMIPMRYRSPLKRHVTRTMDFIHENWKR
IWLVTLWLAANLALFVYKFEQYKHRSSFQVMGNCVCIAKGAAETLKLNMALILLPVC
RNTLTTLRSTALSHVIPFDDNINFHKVLAGAIAVGTVVHTLAHVTCDFPRLISCPSDKFM
ALLGPNFGFRQPTYPDLLASAPGVTGILMIIIMSFSFTLAMHTFRRSVVKLPSPLHHLAG
FNAFWYAHHLLLLVYVLLVVHSYFIFLTRVWYKKTTWMFLIVPVLFYACERIIRKVRE
NNYHVNILKAAIYPGNVLSLHMKKPPGFKYKSGMYLFVKCPDVSPFEWHPFSITSAPG
DDYLSVHIRTLGDWTSELRNLFGKCCEAQVTSKKATLSRLETTVVADSTTEDTRFPKV
FIDGPYGAPAQNYKKYDILLLIGLGIGATPFISILKDLLNNIKSNDEVESIHGSEIGSFKNS
GPGRAYFYWVTREQGSFDWFKGVMNEVADNDHSNVIEMHNYLTSVYEEGDARSALI
AMVQSLQHAKNGVDIVSGSKIRTHFARPNWRKVFSDLANAHKNSRIGVFYCGSPTLTK
QLKDLSKEFSQTTTTRFHFHKENF
>M0WQU0.1/128-225 Uncharacterized protein
{ECO:0000313|EnsemblPlants:HORVU1Hr1G072160.5}
MPNRVGADSGGGGGVGDPVEASGGVHERAPRPGKSARFADPVSAPRGGGGGNDDVE
ITLDVREDSVVVRSVKPVAAGGGEDSGVTPENRSSSSYGHGVLRIASTRDKQVSRELRP
VASFRRRGGGPSRIDRFKPVATHALEGLKFISGTDGAAGWTAAESFFDKKAKNGRLPR

SKFGGCIGMKEAAFAGELFDALARRRNIAGDSINKAELREFWDQISDTSFDSRLQTFLD
MVDKDADGKISEQEVKQIITLSVSANKLTMAPHQCEEYARLIMEALDPHGLRYIELYN
LKMLLLEAPGESTTNNRKLNKLLSERLRPTVDPSLVWRLYRHAKCFLEDNWRRCWV
MLLWLSICVGLFAWKFVQYRHHDVFGVMGYCVCVAKGGAETLKFNMALTLLPVCRN
TITWLRSHTGAGRYVPFNDNLSFHKAIAVGITVGVGLHAISHLACDFPRLLHVMDDEY
GPMKPFFGDNKPPNYWWFVRGTEGWTGLVMLVLMVVAFTFATGPLRKGKLQLPKVK
RLESLDSHPKPIELDRLAMLINTSRSRLTWLVNTSLKCFTGYNAFWYTHHLFLIVYAFLI
VHGHFLYLTKKWQKKSTWMYLAVPMVVYASERLTRALRSSVRSVKKMKVAVHPHP
ATLLSLHLSKPQGFRYKSGQYIFVKCPDVSPSQWHPFSITSAPEDDHVSVHIKAAGDWT
NQLRNAFLKVCSTPTEGKTEILRAEYSRDDVNSNPSFPKVLIDGPYGAPAQDYKEYDIV
LLVGLGIGATPMISIIKDIINNAKRLGGVDVESGNGNGNGNGNASTFRTRRAYFYWVTR
EQGSLEWFRGVMDEVAEADEKRIIELHNHCTSVYGKGDARSALIAMLQSLYYAKNGV
DVVSGSSRVMTHFGRPDWDQVYRRIADENEGKRVGVFYCGEPVLTNKLRRLAKDFSR
NTTTKFKFHSENF
>M0WQU1.1/128-225 Uncharacterized protein
{ECO:0000313|EnsemblPlants:HORVU1Hr1G072160.4}
MPNRVGADSGGGGGVGDPVEASGGVHERAPRPGKSARFADPVSAPRGGGGGNDDVE
ITLDVREDSVVVRSVKPVAAGGGEDSGVTPENRSSSSYGHGVLRIASTRDKQVSRELRP
VASFRRRGGGPSRIDRFKPVATHALEGLKFISGTDGAAGWTAAESFFDKKAKNGRLPR
SKFGGCIGMKEAAFAGELFDALARRRNIAGDSINKAELREFWDQISDTSFDSRLQTFLD
MVDKDADGKISEQEVKQIITLSVSANKLTMAPHQCEEYARLIMEALDPHGLRYIELYN
LKMLLLEAPGESTTNNRKLNKLLSERLRPTVDPSLVWRLYRHAKCFLEDNWRRCWV
MLLWLSICVGLFAWKFVQYRHHDVFGVMGYCVCVAKGGAETLKFNMALTLLPVCRN
TITWLRSHTGAGRYVPFNDNLSFHKAIAVGITVGVGLHAISHLACDFPRLLHVMDDEY
GPMKPFFGDNKPPNYWWFVRGTEGWTGLVMLVLMVVAFTFATGPLRKGKLQLPKVK
RLESLDSHPKPIELDRLAMLINTSRSRLTWLVNTSLKCFTGYNAFWYTHHLFLIVYAFLI
VHGHFLYLTKKWQKKSTWMYLAVPMVVYASERLTRALRSSVRSVKKMKVAVHPHP
ATLLSLHLSKPQGFRYKSGQYIFVKCPDVSPSQWHPFSITSAPEDDHVSVHIKAAGDWT
NQLRNAFLKVFARRRPRGRPRFFGQSTAATTSIPTQASRRC
>M0WRQ5.1/1-45 Predicted protein
{ECO:0000313|EnsemblPlants:HORVU3Hr1G069780.19}
MTESKEFALELFDTLSRRRQMKLDTISKEELREIWQQITDNSFDSRLQIFFDMVDKNAD
GRIGEAEVKEIIMLSASANKLSRLKEQAEEYAALIMEELDPEELGYIELWQLETLLLQK
DTYVNYSQALSYTSQALSQNLALRKRGSIRKIGNSLIYYLEDNWKRLWVLALWIGIMA
GLFTWKFIQYRERYVFSVMGYCVTTAKGAAETLKLNMAIILLPVCRNTITWLRNTRAA
RVLPFDDNINFHKTIAAAIVVGVILHAGNHLVCDFPRLIRSSEETYAPLGIYFGETKPTYL
ALIKGVEGITGIIMVVCMIIAFTLATRWFRRSLVKLPKPFDKLTGFNAFWYSHHLFAIVY
VALIVHGQCVYLIRVWYRKSTWMYLAVPVCLYLGERILRFFRSGSYAVRLLKVAIYPG
NVLTLQMTKPATFRYKSGQYMFVQCPAVSPFEWHPFSITSAPGDEYLSIHVRQLGDWT
RELKRVFSAACEPPVSGKSGLLRADETTKKTLPKLLIDGPYGSPAQDYGKYDVLLLVG
LGIGATPFISILKDLLNNIIKMEEEEDTSTDLYPPVGRNKPHVDLGTLMRVTTRPKKVLK
TTNAYFYWVTREQGSFDWFKGVMNEIAEMDQRNIIEMHNYLTSVYEEGDARSALITM
LQALNHAKNGVDVVSGTKVRTHFARPNWKKVLAKIASKHPYAKIGVFYCGAPVLAQ
ELAKLCHEFNGKCTTKFEFHKEYF
